# Inhibition of Shiga toxin-converting bacteriophage development by novel antioxidant compounds

**DOI:** 10.1080/14756366.2018.1444610

**Published:** 2018-03-14

**Authors:** Sylwia Bloch, Bożena Nejman-Faleńczyk, Karolina Pierzynowska, Ewa Piotrowska, Alicja Węgrzyn, Christelle Marminon, Zouhair Bouaziz, Pascal Nebois, Joachim Jose, Marc Le Borgne, Luciano Saso, Grzegorz Węgrzyn

**Affiliations:** a Department of Molecular Biology, Faculty of Biology, University of Gdansk, Gdansk, Poland;; b Laboratory of Molecular Biology, Institute of Biochemistry and Biophysics, Polish Academy of Sciences, Gdańsk, Poland;; c Université de Lyon, Université Claude Bernard Lyon 1, Faculté de Pharmacie - ISPB, EA 4446 Bioactive Molecules and Medicinal Chemistry, SFR Santé Lyon-Est CNRS UMS3453-INSERM US7, Lyon, France;; d Institut für Pharmazeutische und Medizinische Chemie, PharmaCampus, Westfälische Wilhelms-Universität Münster, Münster, Germany;; e Department of Physiology and Pharmacology “Vittorio Erspamer”, Sapienza University, Rome, Italy

**Keywords:** Shiga toxin-producing *Escherichia coli*, Shiga toxin-converting bacteriophage, oxidative stress, antioxidants, heterocyclic compounds

## Abstract

Oxidative stress may be the major cause of induction of Shiga toxin-converting (Stx) prophages from chromosomes of Shiga toxin-producing *Escherichia coli* (STEC) in human intestine. Thus, we aimed to test a series of novel antioxidant compounds for their activities against prophage induction, thus, preventing pathogenicity of STEC. Forty-six compounds (derivatives of carbazole, indazole, triazole, quinolone, ninhydrine, and indenoindole) were tested. Fifteen of them gave promising results and were further characterized. Eleven compounds had acceptable profiles in cytotoxicity tests with human HEK-293 and HDFa cell lines. Three of them (selected for molecular studies) prevent the prophage induction at the level of expression of specific phage genes. In bacterial cells treated with hydrogen peroxide, expression of genes involved in the oxidative stress response was significantly less efficient in the presence of the tested compounds. Therefore, they apparently reduce the oxidative stress, which prevents induction of Stx prophage in *E. coli.*

## Introduction

The significance of Shiga toxin-producing *Escherichia coli* (STEC) as a public health problem was recognized in 1982 in USA, during an investigation of the outbreak of hemorrhagic colitis associated with consumption of contaminated hamburgers^1^. Since then, STEC strains have been implicated in many outbreaks of diarrhea worldwide. Quite recently (2011), the Shiga toxin-producing *E. coli* serotype O104:H4 was responsible for a serious epidemic outbreak in Germany^2^^,^^3^. STEC pathogens can cause serious food poisoning with bloody diarrhea in humans^4^. Their main virulence factors are Shiga toxins, encoded by *stx* genes located in genomes of bacteriophages, which occur in bacteria as prophages^5^. These bacteriophages are called Shiga toxin-converting or shortly Stx phages and belong to the lambdoid family of phages^6^. All phages within this group indicate high similarities in the lifecycle and genomic organization to bacteriophage λ, the most reviewed member of this family^7^. In the prophage state, most of phage genes, including *stx* genes, are not transcribed due to inhibition caused by the phage cI repressor. Consequently, Shiga toxins are not produced under such conditions. Expression of *stx* as well as other phage genes occurs effectively only after prophage induction. In most cases, this process requires activation of the RecA-dependent bacterial S.O.S. response, which is provoked by factors causing appearance of single-stranded DNA fragments. Activated RecA protein stimulates cleavage of both the SOS regulon repressor, the LexA protein, and the cI phage repressor. Prophage induction and subsequent phage lytic development lead to production of progeny phage particles and Shiga toxins, and then to their release from the lysed cell^8^.

Induction of lambdoid prophages is easy and can be provoked by different inducers like treatment with targeted DNA antibiotics (e.g. mitomycin C), UV-treatment^7^, EDTA^9^, ^60^Co irradiation^10^, high hydrostatic pressure^11^, and many others. Such conditions do not naturally occur in the human gut, the place where the STEC infection initiates. Therefore, the oxidative stress conditions, which appear in human intestine as a response to STEC infection, has attracted attention of researchers. Indeed, it has been shown that hydrogen peroxide (which is excreted by neutrophils during infection) increases efficiency of Stx prophage induction and stimulates production of Shiga toxins^12–14^. In bacteria, H_2_O_2_ acts as a DNA-damaging agent that interacts with free cellular iron and form hydroxyl radicals OH**^•^** in Fenton reaction. DNA subjected to attack by OH**^•^** generates a wide range of base and sugar modifications and strand breaks, which in turn activate the RecA protein and SOS response^15^. According to hypothesis proposed by two different research groups^16^^,^^17^, STEC strains may obtain advantage due to H_2_O_2_-mediated production of Shiga toxins. As demonstrated, these toxins are produced in a small fraction of STEC cells (usually less than 1%) which are sacrificed for the good of the whole bacterial population. Released toxins kill H_2_O_2_-producing protozoan predator, so allowing the rest of bacteria to survive its attack. It is suspected that STEC response to attack of neutrophils in human intestine might be analogous^16^^,^^17^.

The OxyR and SoxR regulators mainly control *E. coli* defense against oxidative stress. Interestingly, H_2_O_2_ activate genes of both regulons: OxyR (about 30 genes) and SoxRS (about 17 genes)^18^. It was demonstrated that nitric oxide and its derivatives (NOs) as well as *S*-nitrosothiols (SNOs) may be involved in signaling in bacteria and may change properties of several bacterial proteins including OxyR, SoxR, NorR and Fur in *Escherichia coli*^19–22^, as well as ResDE in *Bacillus subtilis*^23^. As reported previously, certain SNOs are able to provoke a nitrosative stress in *E. coli* bacteria, evidenced by lowering of intracellular thiol and the transcriptional activation of OxyR by S-nitrosylation^24^. OxyR is a thiol-containing transcriptional activator of antioxidant genes that are involved in H_2_O_2_ detoxification. The activation of *E. coli* antioxidant enzymes by OxyR in response to H_2_O_2_ has been widely described. The genes controlled by OxyR include catalase/hydroperoxidase (*katG*), glutathione reductase (*gorA*), and alkyl hydroperoxide reductase (*ahpCF*). Their expression is essential to ensure resistance to H_2_O_2_^24–26^. As demonstrated previously, S-nitrosylation, S-hydroxylation, and S-glutathionylation of wild-type OxyR activate this protein and appear to serve as the functional switches in the activation of OxyR-regulated genes^24^^,^^27^. On the other hand, nitric oxide (NO) donors are able to activate SoxRS regulon and induce the expression of enzymes involved in the oxidative stress response of *E. coli* bacteria, such as the superoxide dismutase (*sodA*)^28^^,^^29^.

It was previously proposed that OxyR may be responsible for the lambdoid prophage maintenance under conditions of the oxidative stress^8^. Other studies indicated that under such conditions, activated OxyR is able to compete effectively with the cI protein (the main repressor of phage lytic development) for the binding to the *o*_R3_ operator of the *p*_M_-*p*_R_ promoter region^30^. These results allowed to suggest that OxyR might enhance repression of *p*_R_ (the major promoter for expression of genes of phage lytic lifecycle) and enhance activation, and at the same time also downregulate repression, of the transcription of the *c*I gene from the *p*_M_ promoter. This would lead to higher (relative to normal growth conditions) activity of *p*_M_, increased production of cI repressor and lower efficiency of prophage induction under the oxidative stress.

In the light of the importance of the oxidative stress in induction of Stx prophages and Shiga toxin production (summarized above), we analyzed in this study impact of the different antioxidant compounds, on viability of STEC and development of the Stx phage Φ24_B_. Since virulence of STEC depends on the prophage induction, and in the light of the fact that most of antibiotics should not be used for treatment of STEC-infected patients, development of novel drugs that would impair survival of STEC (without causing prophage induction) or prophage induction/development or both would be highly desirable. Although development of novel compounds which might be potentially used to combat *E. coli* infections, exemplified by those based on furanyl-derived sulfonamides, has been reported^31^, and sophisticated techniques become available to either engineer *E. coli* surface^32^ or to use nanoparticles as carriers of compounds^33^, the anti-bacterial activity of these molecules were moderate^31^. Therefore, we have tested a series of novel compounds that are derivatives of carbazole, indazole, triazole, quinolone, ninhydrin, and indenoindole.

## Materials and methods

### Chemistry

Chemical structures of all compounds tested in this report are presented in the supporting information (Supplementary Figures 1–4) with the corresponding references. Seven indenoindole derivatives are described in this paper (CM3159A, MF4, THN10, CM3072B, CM3159B, MF5, MF1). Melting points were determined on an Electrothermal 9200 capillary apparatus. The IR spectra were recorded on a PerkinElmer Spectrum Two IR spectrometer. The ^1^H and ^13^C NMR spectra were recorded on a Bruker DRX 400 spectrometer. Chemical shifts are expressed in ppm (δ) downfield from internal tetramethylsilane, and coupling constants *J* are reported in hertz (Hz). The following abbreviations are used: s, singlet; bs, broad singlet; d, doublet; t, triplet; dd, doubled doublet; q, quartet; sept, septuplet; m, multiplet; Cquat, quaternary carbons. The mass spectra were performed by direct ionization (EI or CI) on a ThermoFinnigan MAT 95 XL apparatus. Chromatographic separations were performed on silica gel columns by column chromatography (Kieselgel 300–400 mesh). All reactions were monitored by TLC on GF254 plates that were visualized under a UV lamp (254 nm). Evaporation of solvent was performed in vacuum with rotating evaporator. The purity of the final compounds (greater than 95%) was determined by uHPLC/MS on an Agilent 1290 system using a Agilent 1290 Infinity ZORBAX Eclipse Plus C18 column (2.1 × 50 mm, 1.8 μm particle size) with a gradient mobile phase of H_2_O/CH_3_CN (90:10, *v/v*) with 0.1% of formic acid to H_2_O/CH_3_CN (10:90, *v/v*) with 0.1% of formic acid at a flow rate of 0.5 ml/min, with UV monitoring at the wavelength of 254 nm. A run time of 10 min was applied. LC/HRMS data of all new indenoindole derivatives are presented in the supporting information.

### General procedure for the synthesis of compound 3

A solution containing equimolar amounts of enaminone **2** and ninhydrin **1** dissolved in MeOH was stirred at room temperature for 19 h. Generally, a precipitate of compound **3** was formed. The solvent was evaporated, and the product triturated, filtrated and washed with EtOAc. A second quantity could be obtained from the filtrate by purification by silica gel column chromatography with CH_2_Cl_2_/acetone (96:4, *v/v*) as the eluent.

*Mixture of regioisomers 4 b,9 b-Dihydroxy-5-isopropyl-1-methoxy-4 b,5,6,7,8,9 b-hexahydroindeno[1,2-b]indole-9,10-dione (major, MAJ) and 4 b,9 b-Dihydroxy-5-isopropyl-4-methoxy-4 b,5,6,7,8,9 b-hexahydroindeno[1,2-b]indole-9,10-dione (minor, min)****CM3072A****:* beige solid. Yield 77%. IR (υ cm^−1^): 3368, 1714, 1595, 1532, 1491 cm^−1^. ^1^H NMR (300 MHz, DMSO-d_6_) δ: 7.73 (t, 1H, *J* = 8.1 Hz, H-2 MAJ), 7.54 (t, 1H, *J* = 7.9 Hz, H-3 min), 7.44 (d, 1H, *J* = 7.5 Hz, H-4 MAJ), 7.38 (d, 1H, *J* = 7.5 Hz, H-3 min), 7.26 (d, 1H, *J* = 7.3 Hz, H-2 min), 7.12 (d, 1H, *J* = 8.3 Hz, H-4 MAJ), 6.66 (s, 1H, OH MAJ), 6.32 (s, 1H, OH min), 5.73 (s, 1H, OH min), 5.54 (s, 1H, OH MAJ), 4.85 (sept, 1H, *J* = 7.1 Hz, CH(CH_3_)_2 _min), 4.54 (sept, 1H, *J* = 7.1 Hz, CH(CH_3_)_2_ MAJ), 3.95 (s, 3H, OCH_3_ min), 3.88 (s, 3H, OCH_3_ MAJ), 2.62–2.82 (m, 4H, H-6 MAJ + min), 2.06–2.13 (m, 4H, H-8 MAJ + min), 1.79–1.91 (m, 4H, H-7 MAJ + min), 1.45 (d, 3H, *J* = 6.8 Hz, CH_3_ MAJ), 1.39 (d, 3H, *J* = 7.2 Hz, CH_3_ min), 1.33 (d, 3H, *J* = 7.0 Hz, CH_3_ min), 1.25 (d, 3H, *J* = 7.1 Hz, CH_3_ MAJ).

*4 b,9 b-Dihydroxy-5-isopropyl-1,4-dimethoxy-4 b,5,6,7,8,9 b-hexahydroindeno[1,2-b]indole-9,10-dione****CM3159A****:* beige solid. Yield 66%. Mp 216 °C. IR (υ cm^−1^): 3347, 1717, 1497, 1270, 999. ^1^H NMR (400 MHz, DMSO-d_6_) δ: 7.30 (d, 1H, *J* = 8.9 Hz, H-3), 7.05 (d, 1H, *J* = 8.9 Hz, H-2), 6.27 (s, 1H, OH), 5.65 (s, 1H, OH), 4.82 (sept, 1H, *J* = 7.1 Hz, CH(CH_3_)_2_), 3.88 (s, 3H, OCH_3_), 3.80 (s, 3H, OCH_3_), 2.70–2.79 (m, 1H, CH_2_), 2.40–2.50 (m, 1H, CH_2_), 2.06 (t, 2H, *J* = 6.3 Hz, CH_2_), 1.75–1.84 (m, 2H, CH_2_), 1.36 (d, 3H, *J* = 7.1 Hz, CH_3_), 1.31 (d, 3H, *J* = 7.1 Hz, CH_3_). ^13 ^C NMR + DEPT (100 MHz, DMSO-d_6_) δ: 194.7 (C = O), 188.7 (C = O), 165.5 (Cquat), 150.6 (Cquat), 149.5 (Cquat), 136.1 (Cquat), 124.3 (Cquat), 118.4 (CH), 112.9 (CH), 106.2 (Cquat), 95.8 (Cquat), 82.9 (Cquat), 55.7 (2 OCH_3_), 45.1 (CH), 36.7 (CH_2_), 24.5 (CH_2_), 22.4 (CH_3_), 22.2 (CH_3_), 21.8 (CH_2_). HRMS calcd for C_20_H_24_NO_6_ [M + H]^+^ 374.1598 found, 374.1599.

*4 b,9 b-Dihydroxy-5-isopropyl-2,3-dimethoxy-4 b,5,6,7,8,9 b-hexahydroindeno[1,2-b]indole-9,10-dione****MF4****:* white solid. Yield 87%. Mp 206 °C. IR (υ cm^−1^): 3071, 1727, 1712, 1488, 1284, 1174, 954. ^1^H NMR (400 MHz, DMSO-d_6_) δ: 7.39 (s, 1H, H-1), 7.09 (s, 1H, H-4), 6.60 (s, 1H, OH), 5.50 (s, 1H, OH), 4.64 (sept, 1H, *J* = 7.1 Hz, CH(CH_3_)_2_), 3.92 (s, 3H, OCH_3_), 3.83 (s, 3H, OCH_3_), 2.44–2.70 (m, 2H, CH_2_), 2.05 (t, 2H, *J* = 6.3 Hz, CH_2_), 1.72–1.85 (m, 2H, CH_2_), 1.43 (d, 3H, *J* = 7.1 Hz, CH_3_), 1.24 (d, 3H, *J* = 7.1 Hz, CH_3_). ^13 ^C NMR + DEPT (75 MHz, DMSO-d_6_) δ: 196.2 (C = O), 188.8 (C = O), 164.2 (Cquat), 155.5 (Cquat), 151.0 (Cquat), 142.9 (Cquat), 127.9 (Cquat), 105.7 (CH), 105.5 (Cquat), 103.6 (CH), 95.4 (Cquat), 82.8 (Cquat), 56.3 (OCH_3_), 55.7 (OCH_3_), 44.7 (CH), 36.7 (CH_2_), 24.3 (CH_2_), 22.6 (CH_3_), 22.1 (CH_3_), 22.0 (CH_2_). HRMS calcd for C_20_H_24_NO_6_ [M + H]^+^ 374.1598, found 374.1603.

*4 b,9 b-Dihydroxy-5-isopropyl-1-nitro-4 b,5,6,7,8,9 b-hexahydroindeno[1,2-b]indole-9,10-dione****AM10A****:* yellow solid. Yield 92%. Mp 227 °C. IR (υ cm^−1^): 3553, 1746, 1589, 1536, 1491. ^1^H NMR (500 MHz, DMSO-*d_6_*) δ: 8.28 (d, 1H, *J* = 7.6 Hz, H-4), 8.04 (d, 1H, *J* = 7.6 Hz, H-2), 7.99 (t, 1H, *J* = 7.6 Hz, H-3), 7.04 (s, 1H, OH), 5.87 (s, 1H, OH), 4.61 (sept, 1H, *J* = 7.2 Hz, CH(CH_3_)_2_), 2.41–2.77 (m, 2H, CH_2_), 2.10 (t, 2H, *J* = 6.0 Hz, CH_2_), 1.84 (m, 2H, CH_2_), 1.45 (d, 3H, *J* = 6.9 Hz, CH_3_), 1.27 (d, 3H, *J* = 6.9 Hz, CH_3_). ^13 ^C NMR + DEPT (125 MHz, DMSO-d_6_) δ: 192.8 (C = O), 188.7 (C = O), 164.9 (Cquat), 148.8 (Cquat), 145.1 (Cquat), 136.1 (CH), 128.9 (CH), 126.5 (Cquat), 124.8 (CH), 104.5 (Cquat), 94.6 (Cquat), 83.3 (Cquat), 44.9 (CH(CH_3_)_2_), 36.7 (CH_2_), 24.2 (CH_2_), 22.5 (CH_3_), 22.4 (CH_3_), 21.9 (CH_2_). HRMS calcd for C_18_H_19_N_2_O_6_ [M + H]^+^, 359.1238 found: 359.1240.

### General procedure for the synthesis of compound 4

A solution containing 5.92 mmol of **3** and 11.5 mmol (2 equiv) of TETA dissolved in 18 ml of DMF and 3.5 ml of AcOH was stirred at room temperature for 22 h. The solution was then poured into 50 ml of ice and water and stirred for 30 min. The resulting precipitate was filtered, washed with water and dried to give a first quantity of **4**. EtOAc was added to the filtrate, and the organic product extracted. The organic phase was washed with water, dried over anhydrous Na_2_SO_4_ and evaporated in vacuum to give a second quantity of **4** which was purified by silica gel column chromatography with CH_2_Cl_2_/acetone (95:5, *v/v*) as the eluent.

*5-Isopropyl-1-methoxy-5,6,7,8-tetrahydroindeno[1,2-b]indole-9,10-dione****THN10***: brown solid. Yield 50%. Mp 186 °C. IR (υ cm^−1^): 1694, 1661, 1590, 1261, 1023, 803. ^1^H NMR (400 MHz, DMSO-d_6_) δ: 7.38 (dd, 1H, *J_1_* = 8.6 Hz, *J_2_* = 7.5 Hz, H-3), 7.04 (d, 1H, *J* = 7.3 Hz, H-4), 6.98 (d, 1H, *J* = 8.8 Hz, H-2), 4.77 (sept, 1H, *J* = 6.9 Hz, CH(CH_3_)_2_), 3.86 (s, 3H, OCH_3_), 2.93 (m, 2H, CH_2_), 2.37 (m, 2H, CH_2_), 2.08 (m, 2H, CH_2_), 1.57 (d, 6H, *J* = 7.1 Hz, CH_3_). ^13 ^C NMR + DEPT (100 MHz, DMSO-d_6_) δ: 191.2 (C = O), 182.2 (C = O), 156.8 (2 Cquat), 150.1 (2 Cquat), 136.8 (2 Cquat), 135.1 (CH), 121.2 (Cquat), 114.7 (CH), 112.8 (CH), 55.6 (OCH_3_), 49.5 (CH(CH_3_)_2_), 37.7 (CH_2_), 22.8 (2 CH_2_), 21.3 (2 CH_3_). HRMS calcd for C_19_H_20_NO_3_ [M + H]^+^ 310.1438, found: 310.1438.

*5-Isopropyl-4-methoxy-5,6,7,8-tetrahydroindeno[1,2-b]indole-9,10-dione****CM3072B***: red solid. Yield 24%. Mp 224 °C. IR (υ cm^−1^): 3436, 1704, 1660, 1600, 1488, 1270. ^1^H NMR (400 MHz, DMSO-*d_6_*) δ: 7.19–7.28 (m, 2H, H-1 and H-2), 7.02 (d, 1H, *J* = 8.8 Hz, H-3), 5.67 (m, 1H, CH(CH_3_)_2_), 2.94 (s, 3H, OCH_3_), 3.00 (m, 2H, CH_2_), 2.38 (t, 2H, *J* = 6.3 Hz, CH_2_), 2.07 (t, 2H, *J* = 6.0 Hz, CH_2_), 1.56 (d, 6H, *J* = 7.0 Hz, CH_3_). ^13 ^C NMR + DEPT (100 MHz, DMSO-d_6_) δ: 191.4 (C = O), 183.1 (C = O), 150.2 (Cquat), 149.8 (2 Cquat), 139.6 (2 Cquat), 130.6 (CH), 121.2 (2 Cquat), 119.2 (CH), 116.3 (CH), 56.3 (OCH_3_), 51.0 (CH(CH_3_)_2_), 37.7 (CH_2_), 24.5 (CH_2_), 23.4 (CH_2_), 21.6 (2 CH_3_). HRMS calcd for C_19_H_20_NO_3_ [M + H]^+^ 310.1438, found: 310.1435.

*1,4-Dimethoxy-5-isopropyl-5,6,7,8-tetrahydroindeno[1,2-b]indole-9,10-dione****CM3159B***: orange-red solid. Yield 75%. Mp 247 °C. IR (υ cm^−1^): 1693, 1661, 1494, 1262. ^1^H NMR (400 MHz, DMSO-d_6_) δ: 7.19 (d, 1H, *J* = 9.3 Hz, H-3), 6.99 (d, 1H, *J* = 9.3 Hz, H-2), 5.71 (m, 1H, CH(CH_3_)_2_), 3.90 (s, 3H, OCH_3_), 3.81 (s, 3H, OCH_3_), 2.99 (m, 2H, CH_2_), 2.36 (m, 2H, CH_2_), 2.06 (m, 2H, CH_2_), 1.54 (d, 6H, *J* = 7.1 Hz, CH_3_). ^13 ^C NMR + DEPT (75 MHz, DMSO-d_6_) δ: 191.2 (C = O), 181.7 (C = O), 151.9 (2 Cquat), 149.9 (Cquat), 144.3 (2 Cquat), 122.8 (Cquat), 122.2 (Cquat), 121.5 (CH), 117.1 (CH), 56.0 (2 OCH_3_), 50.7 (CH(CH_3_)_2_), 37.7 (2 CH_2_), 23.4 (CH_2_), 21.6 (CH_3_). HRMS calcd for C_20_H_21_NNaO_4_ [M + Na]^+^ 362.1363, found: 362.1364.

*2,3-Dimethoxy-5-isopropyl-5,6,7,8-tetrahydroindeno[1,2-b]indole-9,10-dione****MF5***: red solid. Yield 74%. Mp 245 °C. IR (υ cm^−1^): 1696, 1659, 1268. ^1^H NMR (400 MHz, DMSO-d_6_) δ: 7.04 (s, 1H, H-1), 6.97 (s, 1H, H-4), 4.77 (sept, 1H, *J* = 6.9 Hz, CH(CH_3_)_2_), 3.93 (s, 3H, OCH_3_), 3.84 (s, 3H, OCH_3_), 2.94 (m, 2H, CH_2_), 2.38 (t, 2H, *J* = 6.0 Hz, CH_2_), 2.10 (t, 2H, *J* = 5.9 Hz, CH_2_), 1.61 (d, 6H, *J* = 6.5 Hz, CH_3_). ^13 ^C NMR + DEPT (100 MHz, DMSO-d_6_) δ: 190.6 (C = O), 182.6 (C = O), 151.4 (Cquat), 150.6 (Cquat), 148.8 (Cquat), 148.1 (Cquat), 130.8 (Cquat), 128.6 (Cquat), 118.6 (Cquat), 116.9 (Cquat), 109.2 (CH), 106.0 (CH), 56.3 (OCH_3_), 55.9 (OCH_3_), 48.9 (CH(CH_3_)_2_), 37.3 (CH_2_), 22.6 (CH_2_), 22.5 (CH_2_), 21.2 (2 CH_3_). HRMS calcd for C_20_H_21_NNaO_4_ [M + Na]^+^ 362.1363, found: 362.1366.

*5-Isopropyl-1-nitro-5,6,7,8-tetrahydroindeno[1,2-b]indole-9,10-dione****AM12B***: red solid. Yield 80%. Mp 244 °C. IR (υ cm^−1^): 1707, 1666, 1605, 1536. ^1^H NMR (400 MHz, DMSO-d_6_) δ: 7.63 (d, 1H, *J* = 7.5 Hz, H-2), 7.59 (t, 1H, *J* = 7.5 Hz, H-3), 7.49 (d, 1H, *J* = 7.0 Hz, H-4), 4.80 (sept, 1H, *J* = 7.0 Hz, CH(CH_3_)_2_), 2.95 (m, 2H, CH_2_), 2.37 (t, 2H, *J* = 6.0 Hz, CH_2_), 2.06 (m, 2H, CH_2_), 1.56 (d, 6H, *J* = 7.0 Hz, CH_3_). ^13 ^C NMR + DEPT (100 MHz, DMSO-d_6_) δ: 190.8 (C = O), 177.8 (C = O), 155.7 (2 Cquat), 149.7 (Cquat), 145.5 (Cquat), 136.0 (2 Cquat), 134.9 (CH), 127.4 (Cquat), 121.6 (CH), 121.0 (CH), 49.5 (CH(CH_3_)_2_), 37.3 (CH_2_), 22.8 (CH_2_), 22.5 (CH_2_), 21.2 (2 CH_3_). HRMS calcd for C_18_H_16_N_2_NaO_4_ [M + Na]^+^, 347.1002 found: 347.0989.

*1-Amino-5-isopropyl-5,6,7,8-tetrahydroindeno[1,2-b]indole-9,10-dione****MF1***: To a stirred solution of nitro derivative **AM12B** (485 mg, 1.50 mmol) in 4.9 ml of glacial acetic and 3.4 ml of water at 90 ***°***C was added iron (686 mg, 12.2 mol) in portions. The resulting suspension was stirred at the same temperature for 45 min. Then, the reaction mixture was cooled to room temperature and basified with saturated NaHCO_3_ aqueous solution. The organic phase was extracted with dichloromethane washed with brine, dried with Na_2_SO_4_, filtered and evaporated to dryness. The residue was purified by silica gel column chromatography with CH_2_Cl_2_/acetone (1:1, *v/v*) as the eluent to afford 254 mg of **MF1**: yellow solid. Yield 58%. Mp 238 °C. IR (υ cm^−1^): 3448, 3351, 1673, 1625, 1463. ^1^H NMR (400 MHz, DMSO-d_6_) δ: 7.07 (t, 1H, *J* = 7.7 Hz, H-3), 6.64 (d, 1H, *J* = 7.1 Hz, H-2), 6.57 (d, 1H, *J* = 8.6 Hz, H-4), 6.34 (bs, 2H, NH_2_), 4.72 (sept, 1H, *J* = 7.0 Hz, CH(CH_3_)_2_), 2.93 (t, 2H, *J* = 6.0 Hz, CH_2_), 2.38 (t, 2H, *J* = 6.0 Hz, CH_2_), 2.07–2.13 (m, 2H, CH_2_), 1.57 (d, 6H, *J* = 6.8 Hz, CH_3_). ^13 ^C NMR + DEPT (100 MHz, DMSO-d_6_) δ: 191.0 (C = O), 186.4 (C = O), 149.1 (Cquat), 148.3 (Cquat), 146.8 (Cquat), 134.8 (Cquat), 133.3 (CH + Cquat), 118.8 (CH), 116.3 (Cquat), 114.0 (Cquat), 109.0 (CH), 48.7 (CH(CH_3_)_2_), 37.5 (CH_2_), 22.7 (2 CH_2_), 20.9 (2 CH_3_). HRMS calcd for C_18_H_18_N_2_NaO_2_ [M + Na]^+^ 317.1260, found: 317.1259.

### Prophage induction experiment

*E. coli* MG1655 strain^34^ lysogenic for phage Φ24_B_ Δ*stx2*::*cat*^35^ was cultured in LB medium at 37 °C with one of the analyzed compounds (Supplementary Figures S1–S4), in the presence or absence of the prophage inductor: 0.2 µg/ml; 0.5 µg/ml mitomycin C or 1 mM H_2_O_2_. In control experiments, DMSO (a solvent used for preparation of stock solutions) was added instead of the tested compound. Each analyzed compound was added at the beginning of the culture (time 0), at three different concentrations (50, 100 and 200 μM), and selected prophage inductor was added after 3 h. Measurements of the bacterial cultures optical density at 600 nm (A_600_) were performed at indicated times (0, 3, 5, 7, and 9 h). Six hours after an induction (at the ninth hour of the experiment), 50 μl samples were withdrawn, mixed with 10 µl of chloroform and centrifuged (4,500 rpm, 5 min). Serial dilutions were prepared in TM buffer (10 mM Tris–HCl, 10 mM MgSO_4_; pH 7.2). Phage titer (number of phages per ml) was determined by spotting 2.5 μl of each dilution of the phage lysate on a freshly prepared LB agar (1.5%) with 2.5 μg/ml chloramphenicol, to obtain visible plaques formed on bacterial lawn (according to a procedure described previously^36^), with a poured mixture of 1 ml indicator *E. coli* MG1655 strain culture and 2 ml of 0.7% nutrient agar (prewarmed to 45 °C), supplemented with MgSO_4_ and CaCl_2_ (to a final concentration of 10 mM each). Plates were incubated at 37 °C overnight. Each experiment was repeated three times. The relative phage titer (PFU/ml) representing ratios of phage titers in induced and non-induced cultures was calculated and presented as percentage of relative phage titer obtained in control experiments, with DMSO added instead of the analyzed compound.

***MTT cell viability assay***: 3 × 10^3^ HEK-293 (Human Embryonic Kidney) or HDFa (Human Dermal Fibroblast, adult) cells were passaged in each well of 96-well plate, and allowed to attached overnight. Cells were then treated with DMSO (control cells) or 50, 100 or 200 µM of tested compound at 37 °C. After 48 h incubation, 25 µl of MTT solution (4 mg/ml) was added to each well. Following 3 h incubation at 37 °C, formazan crystals, formed in living cells, were dissolved in 100 µl of DMSO. Absorbance was measured at 570 and 620 nm (reference wavelength) in a Victor^3^ microplate reader.

***Bacterial RNA preparation***: For the extraction of total RNA from *E. coli* strain MG1655 lysogenic for Φ24_B_ bacteriophage, the procedure described in previous subsection was employed with slight modification. Three selected compounds: **CM092**, **CM032D,** and **CM3186** were added to the bacterial culture at time 0 to a final concentration 200 µM. The control experiment was treated with DMSO solution, which allows estimation of impact of tested compounds on prophage induction. After 3 hours of bacterial cultivation at 37 °C, phage lytic development was provoked by addition of 1 mM H_2_O_2._ At indicated time after prophage induction, 1 × 10^9^ bacterial cells were treated with 10 mM NaN_3_ (Sigma-Aldrich), harvested and frozen in a liquid nitrogen. The RNA samples were prepared using the High Pure RNA Isolation Kit (Roche Applied Science) and TURBO DNA-free^TM^ Kit (Life Technologies) according to the manufacturer’s guidelines. The amount and quality of RNA were evaluated by using NanoDrop spectrophotometer (Eppendorf). In addition, the band patterns of total RNA was visualized by agarose gel electrophoresis. Moreover, the absence of DNA was verified by PCR amplification, and by quantitative real-time reverse transcription-PCR (qRT-PCR).

***cDNA synthesis via reverse transcription***: Each cDNA was prepared using Transcriptor Reverse Transcriptase and random hexamer primers (Roche Applied Science) following the protocols supplied from the provider. 1.25 µg of the total RNA sample was used as a template. The resulting cDNA mixture was diluted 10-fold and analyzed in qRT-PCR.

***Real-time PCR assay***: qRT-PCR was performed with the LightCycler 480 Real-Time PCR System (Roche Applied Science) according to the procedure described previously^14^^,^^37^^,^^38^. Reactions were prepared in Roche 96-well plates containing: 10 µl 2 × SYBR Green I Master Mix (Roche Applied Science), 6.25 µl/ml cDNA, and 200 nM specific primers (Table 1). Real-time PCR amplification were carried out for 55 cycles and cycling conditions were as follows: an initial incubation at 95 °C for 5 min, followed by denaturation at 95 °C for 10 s, annealing at 60 °C for 15 s, and extension at 72 °C for 5 s. No-template controls and a melting curve analysis were performed for each reaction. The 16S rRNA gene was used as reference gene that showed no changes in expression levels in the presence of H_2_O_2_ and tested compounds. Each experiment was repeated three times.

**Table 1.** Primers used in the real-time PCR assay.

Primer name	Sequence (5′→3′)
pF_OxyR pR_OxyR	GCAGGTAGCGGGATCACTTT GCACGGCAGATAAACAACCC
pF_KatG pR_KatG	GCTCTGCCTGTTCTGGAGAA CACACCAGCCAGCACTATGA
pF_AhpC pR_AhpC	GCTGGAGCGTCTTCTTCTTCT TAGTGGTCAGCAACGTCACC
pF_CysD pR_CysD	ATTTGCCCGTTGTAGTTGTGC TACTCTTTCCGTGACCGCTTC
pF_SodA pR_SodA	CTGCCAGAATTTGCCAACCTG GTACGGTTTTCTTGTCTGCTGG
pF_SoxR pR_SoxR	AAACAGCTTTCGTCCCAATGG TACATCCGTCCAGTTCGTCAC
pF_SoxS pR_SoxS	GCTGGGAGTGCGATCAAACT GCAATGGACCTGGGTTATGTGT
pF_16SrRNA pR_16SrRNA	CCTTACGACCAGGGCTACAC TTATGAGGTCCGCTTGCTCT
pF_Φ24_B__N pR_ Φ24_B__N	AGGCGTTTCGTGAGTACCTT TTACACCGCCCTACTCTAAGC
pF_Φ24_B__cI pR_Φ24_B__cI	TGCTGTCTCCTTTCACACGA GCGATGGGTGGCTCAAAATT
pF_Φ24_B__cII pR_Φ24_B__cII	TGATCGCGCAGAAACTGATTTAC GACAGCCAATCATCTTTGCCA
pF_Φ24_B__Q pR_Φ24_B__Q	GGGAGTGAGGCTTGAGATGG TACAGAGGTTCTCCCTCCCG

***Real-time PCR analysis***: For precise relative quantification of changes in the gene expression, the E-Method with efficiency correction was chosen. This method has been used and described in detail by us previously^37–39^. The sample before addition of the induction agent (3 h after inoculation) was a calibrator. The final results were calculated using the following formula: Normalized relative ratio = *Et*^CT(*t*) calibrator − CT(*t*) sample^/*Er*^CT(*r*) calibrator − CT(*r*) sample^, where *E* is efficiency, *t* is target and *r* is reference. The raw run data for *E. coli* strain MG1655 and Φ24_B_ genes were transferred to the LC480Conversion software and the efficiency and PCR efficiency was calculated for each gene by LinRegPCR program.

## Results

We have analyzed effects of 46 compounds (Supplementary Figures S1–S4) on growth of *E. coli* MG1655 bacteria lysogenic with phage Φ24_B_ (a Shiga-toxin converting phage). Density of bacterial culture was measured at indicated time after prophage induction with 0.2 µg/ml mitomycin C in the presence and absence of analyzed compounds. The results are presented in Table 2.

**Table 2.** Differences in OD values resulting from comparison of bacterial growth (*E. coli* strain MG1655 lysogenic for Φ24_B_) in control culture (DMSO) and cultures carried out with the analyzed compounds. Each experiment was conducted during 9 h, in the presence or absence of mitomycin C (MITC).

		Effects on the lysogenic strain growth^a^					
		50 µM		100 µM		200 µM	
No	Compound	–MITC	+ MITC	–MITC	+ MITC	–MITC	+ MITC
1	BZ23	–12	9	–12	5	–22	12
2	BZ26	7	52	–1	48	–2	>100
3	BZ102	–1	12	–22	2	–25	8
4	BZ105	–23	–16	–20	–29	30	–44
5	BZ106	–2	–11	–7	–68	–97	–96
6	BZ86	1	29	–2	24	–18	15
7	BZ70	0	41	–3	54	–7	40
8	BZ83	12	12	7	–2	–7	–18
9	BZ64	0	26	0	12	–18	11
10	BZ96	1	10	–1	15	–7	–2
11	BZ89	–2	8	2	–6	2	–36
12	IA011C	14	83	18	>100	16	>100
13	CM092	6	>100	6	>100	–11	>100
14	CM022G	1	48	4	48	–1	68
15	CM032D	3	17	6	49	0	65
16	CM2071F	4	>100	4	>100	–7	>100
17	CM3186B	2	65	9	72	7	>100
18	CM3141B	9	23	12	57	8	>100
19	BZA15	8	>100	17	>100	15	>100
20	AR02	–1	4	–3	20	–8	41
21	AR09	1	10	2	17	–9	45
22	BZA23	4	8	3	7	–5	76
23	CM3116A	0	18	–1	22	–9	20
24	CM3159A	4	27	5	26	–1	42
25	CM3146B	–3	4	–4	11	–14	40
26	CM3129A	0	21	5	28	–30	3
27	MF1	2	15	0	41	–1	>100
28	MF27A	–2	9	–2	25	–6	68
29	THN10	3	21	–1	32	–2	>100
30	THN6C	11	20	9	51	16	>100
31	CM3072B	3	10	3	3	2	76
32	CM3116C	5	1	3	3	–4	77
33	MF5	–1	44	–1	97	–7	>100
34	CM3159B	–1	30	1	68	0	>100
35	AR27	0	–4	–4	–1	–14	69
36	MQ4	3	–23	–2	–18	–15	45
37	MQ8	7	–8	–9	–10	–11	18
38	BZA37	10	–6	9	10	2	75
39	CM4017A	0	3	–6	19	–11	38
40	CM3130B	–6	23	–17	22	–26	29
41	CM4016A	–1	6	–4	18	–11	58
42	CM3112B	0	40	–15	1	–26	12
43	SiA5	3	25	–14	25	–17	29
44	CM032E	–2	19	–4	13	–12	20
45	MF4	–2	4	–2	41	–10	91
46	MF6	–2	1	–14	13	–13	92

^a^Presented results are expressed as percent values above (numbers) or under (numbers with minus sign) the control value which is assumed as 100%. SD was below 20% for each point, and it is not shown for clarity of presentation. Compounds selected for further analyses are underlined.

Based on the analysis of growth inhibition of bacterial cultures, 15 compounds were selected to further studies. Most of them were able to increase OD value of the mitomycin C-treated bacterial culture, above 100% in at least one analyzed concentration.

**Figure 1.**
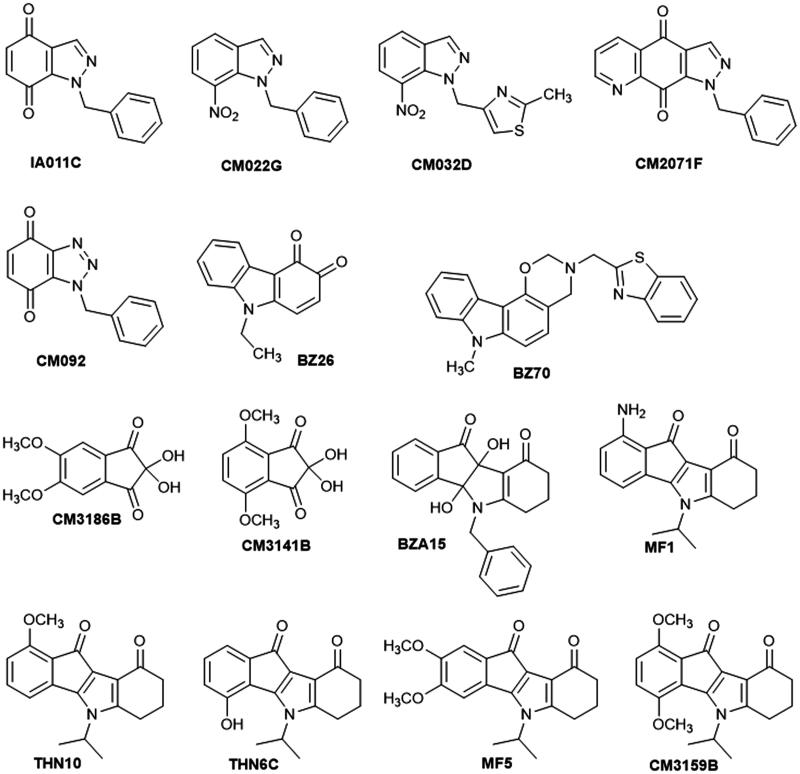
Structures of the selected 15 compounds.

The 15 compounds selected for further biological exploration are presented in Figure 1. Indazole-4,7-dione **IA011C** was synthesized in two steps from **CM022G**: reduction of the nitro group by hydrogenation in acidic medium followed by oxidation by PIFA^40^. Nitroindazoles **CM022G**^40^ and **CM032D**^41^ were prepared by *N*-alkylation of the commercially available 7-nitro-*1H*-indazole with respectively benzyl bromide or 4-(chloromethyl)-2-methylthiazole as alkylating agent. Pyrazolo[4,5-*g*]quinoline-4,9-dione **CM2071F** was obtained by a Diels-Alder reaction between *N,N*-dimethylhydrazone and indazole-4,7-dione **IA011C**^42^. Benzotriazole-4,7-dione **CM092** was obtained by a 1,3-dipolar cycloaddition of benzyl azide^43^ on commercial *para*-benzoquinone^40^. Carbazole-3,4-quinone **BZ26** was obtained in two steps from the commercially available 9-ethylcarbazol-3-amine. The formation of the corresponding diazonium salt at 0 °C followed by heating the latter in highly acidic aqueous solution gave 9-ethylcarbazol-3-ol, which was finally oxidized with Frémy’s salt^44^.

**Figure 2.**
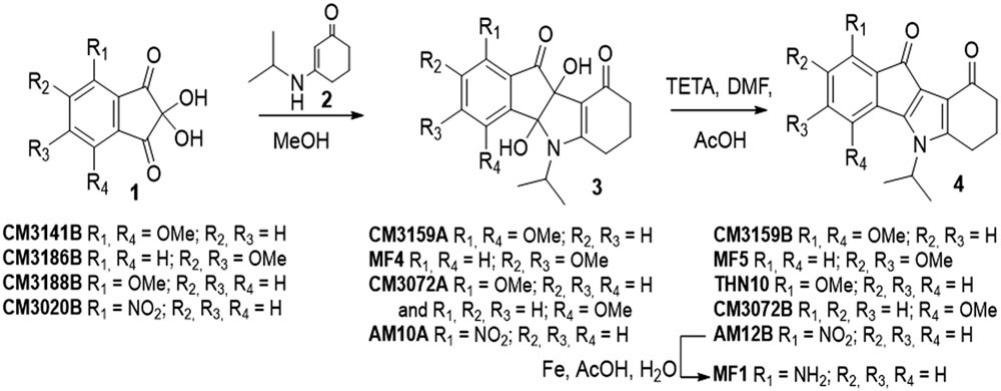
Chemical procedure for the synthesis of targeted indenoindoles.

Oxazinocarbazole **BZ70** was prepared by a Mannich type condensation of benzo[*d*]thiazol-2-ylmethanamine and formaldehyde with 9-methylcarbazol-4-ol. This latter was obtained by a chemoselective N-alkylation of the commercially available carbazol-4-ol which was achieved by generating the N,O-dianion with NaH in a DMF/THF mixture under argon atmosphere at room temperature and subsequent treatment with methyl iodide^45^. Dimethoxynin-hydrins (2,2-dihydroxydimethoxyindane-2,3-diones) **CM3186B** and **CM3141B** were prepared in a single step from the corresponding commercial substituted indan-1-ones by microwave-assisted selenium oxidation^46^. Dihydroxyindeno[1,2-*b*]indole-9,10-dione **BZA15**^47^ was prepared by condensation of ninhydrin and 3-(benzylamino)cyclohex-2-en-1-one. 5-Isopropylindeno[1,2-*b*]indole-9,10-dione derivatives **MF1**^48^, **THN10**, **THN6C**^49^, **MF5**, and **CM3159B**, were synthesized in two steps according to the method previously described^47^: first, condensation between 3-(isopropylamino)cyclohex-2-en-1-one **2**^47^ and the corresponding substituted ninhydrin, then deoxygenation using tetraethylthionylamide (TETA) (Figure 2).

Use of dimethoxyninhydrins **CM3141B** and **CM3186B** afforded the dihydroxydimethoxyindeno[1,2-*b*]indole-9,10-diones **CM3159A** and **MF4**, respectively, which were easily deoxygenated to give the expected dimethoxyindenoindoles **CM3159B** and **MF5**.

**Figure 3.**
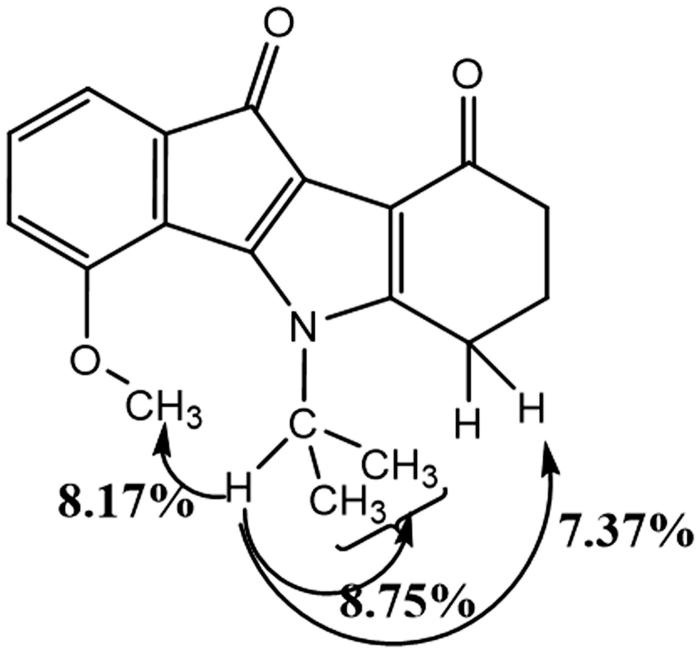
Correlations observed from NOE experiments for **CM3072B**.

Condensation of enaminone **2** with 4-methoxyninhydrin **CM3188B**^40^ led to a mixture of the two regiosiomers 1- and 4-methoxy (ratio 69/31) which could not be separated under classical conditions. After deoxygenation of the mixture, the two regioisomers 1- and 4-methoxyindenoindoles were easily separated by column chromatography and their assignment established by NMR experiments. NOE experiment for the regioisomer **CM3072B** (Figure 3) showed a correlation between the CH of the isopropyl group and the methoxy group.

**Figure 4.**
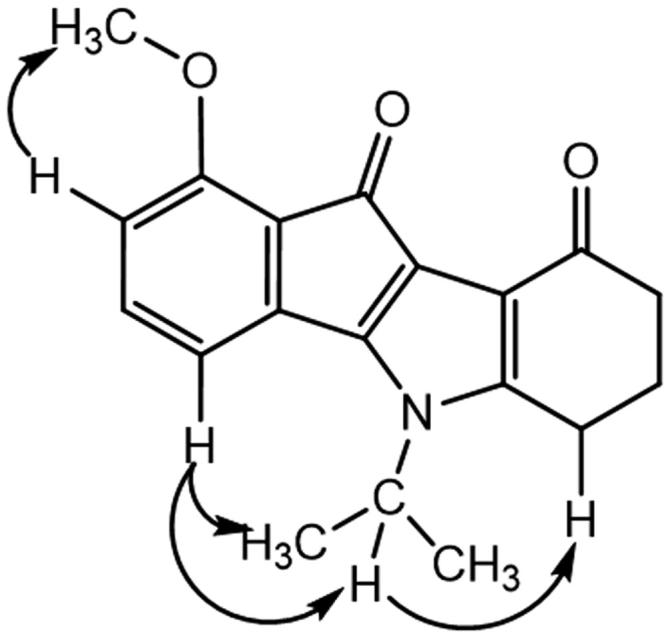
Correlations observed from NOESY experiment for **THN10**.

This correlation was not observed for the other regioisomer, compound **THN10**. NOESY experiment for this latter showed correlations (Figure 4) between the CH of the isopropyl group and an aromatic proton (H-4), confirming unambiguously the assignment.

**Figure 5.**
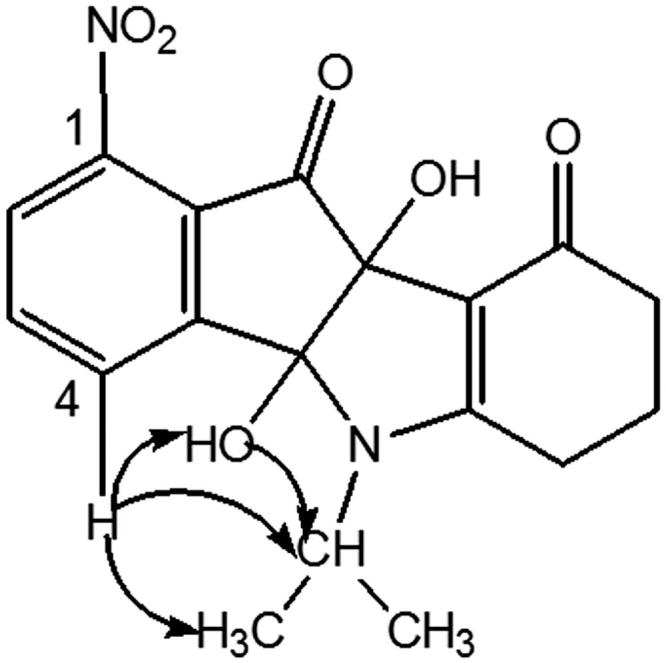
NOESY interactions for **AM10A**.

Amino derivative **MF1** was prepared from 4-nitroninhydrin **CM3020B**^40^. The first coupling step led to the single 1-nitro regioisomer **AM10A**. Assignment was established by NMR experiments: 2 D NMR HSQC and ^1^H-^13 ^C HMBC correlations, using the characteristic long-range ^3^*J* and ^4^*J* couplings and NOESY. All of the cross couplings observed are reported in the Table 3, and the decisive correlations for the structural assignment are summarized in Figure 5.

**Table 3.** 2 D ^1^H–^13 ^C HMBC correlations for AM10A (DMSO, 500.13 MHz).


			HMBC [*J*(C,H)]			
Atom	^13^C	^1^H	^1^*J*	^2^*J*	^3^*J*	^4^*J*
**1**	145.1			2-H,	3-H	4-H
**2**	124.8	8.04	2-H			
**3**	136.1	7.99	3-H			
**4**	128.9	8.28	4-H			
**4a**	148.8			4-H	3-H, 4b-OH	2-H
**4b**	94.6				4-H, 4b-OH, 1’-H,	3-H, 9b-OH
**5a**	164.9				7-H,1’-H	8-H
**6**	36.7	2.10	6-H	7-H	8-H	
**7**	21.9	1.84	7-H	6-H, 8-H		
**8**	24.2	2.41-2.77	8-H	7-H	6-H	
**9**	188.7				7-H	6-H
**9a**	104.5				6-H, 8-H, 9b-OH	
**9b**	83.3			9b-OH	4b-OH	
**10**	192.8				9b-OH	2-H
**10a**	126.4				2-H, 4-H	3-H
1’	44.9	4.61	1’-H	CH_3_		

The carbonyls C-9 and C-10 were firstly attributed due to the ^3^*J* and ^4^*J* couplings between the protons 6-H and 7-H with C-9 on the one hand, and OH-9 b and 2-H with C-10 on the other hand. Then assignment of the C-4 b and C-9 b was allowed. In fact, only the OH-9 b proton correlated with C-10 (^3^*J*_H,C_). Next, the cross couplings found in the NOESY between the protons 4-H with OH-4 b and the CH of isopropyl group allowed us to identify unambiguously the proposed regiochemistry (Figure 5). This derivative **AM10A** was then dedihydroxylated with TETA to afford **AM12B**. Finally, the nitro group was reduced into amine **MF1** using iron in acidic conditions^50^.

**Figure 6.**
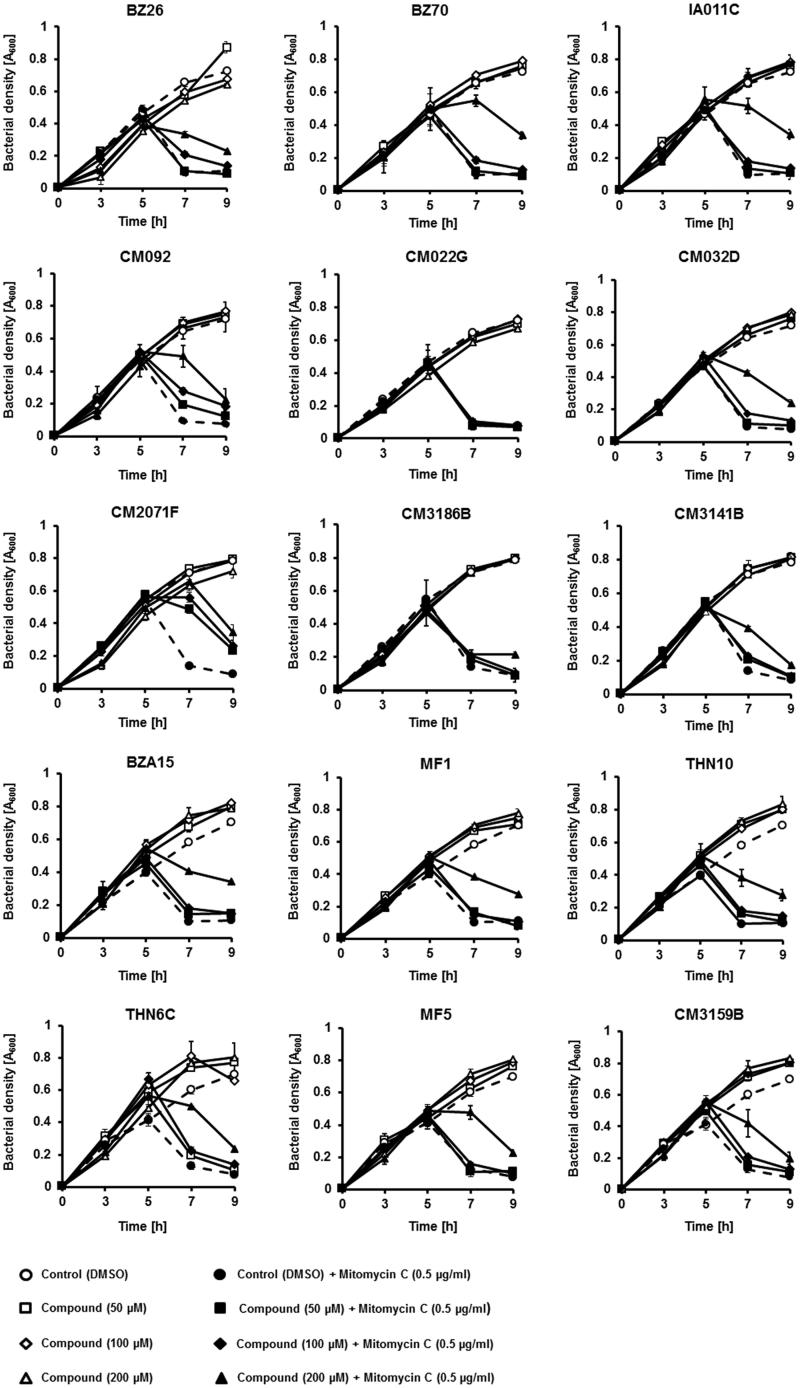
Growth of *E. coli* MG1655 lysogenic with Φ24_B_Δ*stx2*::*cat* at 37 °C in LB medium after induction with 0.5 µg/ml mitomycin C (added to the culture at time 3 h) in the absence or presence of tested compounds at indicated concentrations (added to the culture at time 0). Bacterial growth was monitored by measurement of A_600_ at indicated times. Presented results are mean values from three experiments with SD indicated as error bars.

Detailed analysis of effects of the tested 15 compounds on bacterial growth revealed various activities of different substances. Induction of the lysogenic bacterial culture with mitomycin C caused prophage induction and subsequent lysis of the host cells, seen in Figure 6 as a decrease in the cell culture optical density.

**Figure 7.**
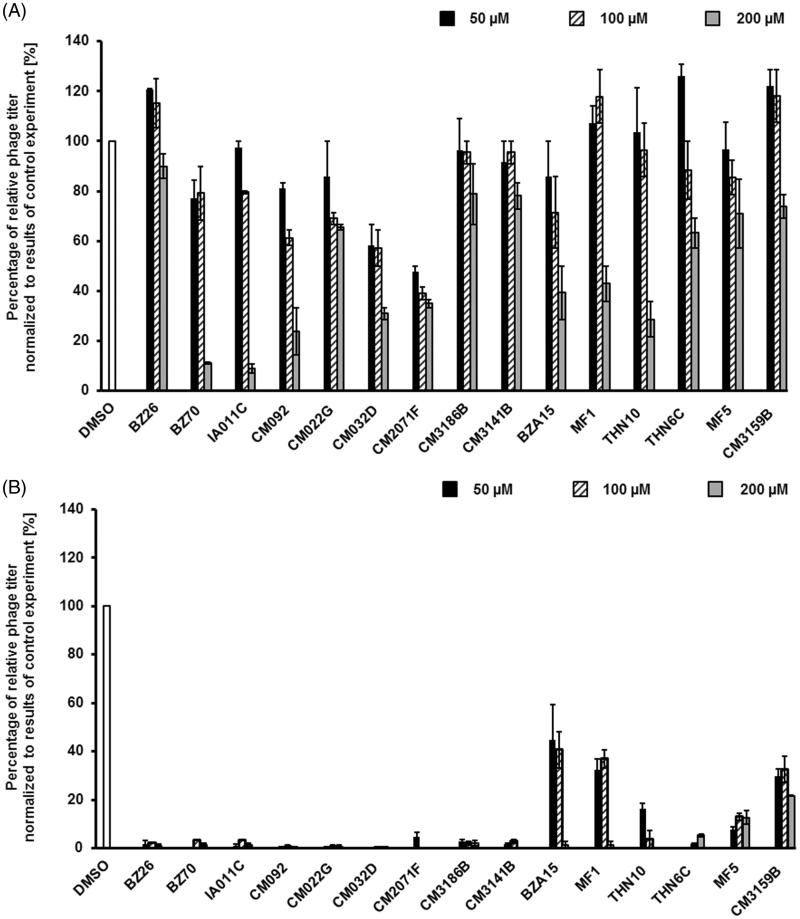
Relative phage titer in cultures of *E. coli* MG1655 lysogenic with Φ24_B_Δ*stx2*::*cat* treated with 0.5 µg/ml mitomycin C (A) or 1 mM H_2_O_2_ (B) (inducers were added to the culture at time 3 h) in the absence (control experiments) or presence of tested compounds at indicted concentrations (added to the culture at time 0). Presented results are mean values from three experiments with SD indicated as error bars.

However, majority of the selected compounds (BZ26, BZ70, IA011C, CM092, CM032D, CM2071F, CM3141B, BZA15, MF1, THN10, THN6C, MF5, CM3159B) ameliorated this effect, preventing bacterial cell lysis after treatment with mitomycin C, in at least one used concentration (Figure 6). These results suggested that such compounds might prevent either effective prophage induction or phage lytic development. Hence, we have measured titers of bacteriophages in cultures treated with mitomycin C with and without the presence of tested compounds. In addition, hydrogen peroxide was used as an inducing agent, as this substance was found previously as a prophage inducer occurring naturally in human gut. Contrary to mitomycin C, this inducer did not cause detectable changes in the density of bacterial culture (data not shown). In mitomycin C-treated lysogenic *E. coli* cell culture, among the 15 selected compounds, the presence of most of them in the culture caused a significant decrease in the titer of bacteriophage (Figure 7(A)). Importantly, in hydrogen peroxide-treated cells, the effects of these compounds on phage titer were dramatic (Figure 7(B)).

These results indicate that in the presence of compounds **BZ26**, **BZ70**, **IA011C**, **CM092**, **CM022G**, **CM032D**, **CM2071F**, **CM3186B**, **CM3141B**, **THN6C**, Φ24_B_ prophage induction and/or phage lytic development is/are impaired. This suggest that at least some of them might be considered as potential anti-STEC drugs.

**Figure 8.**
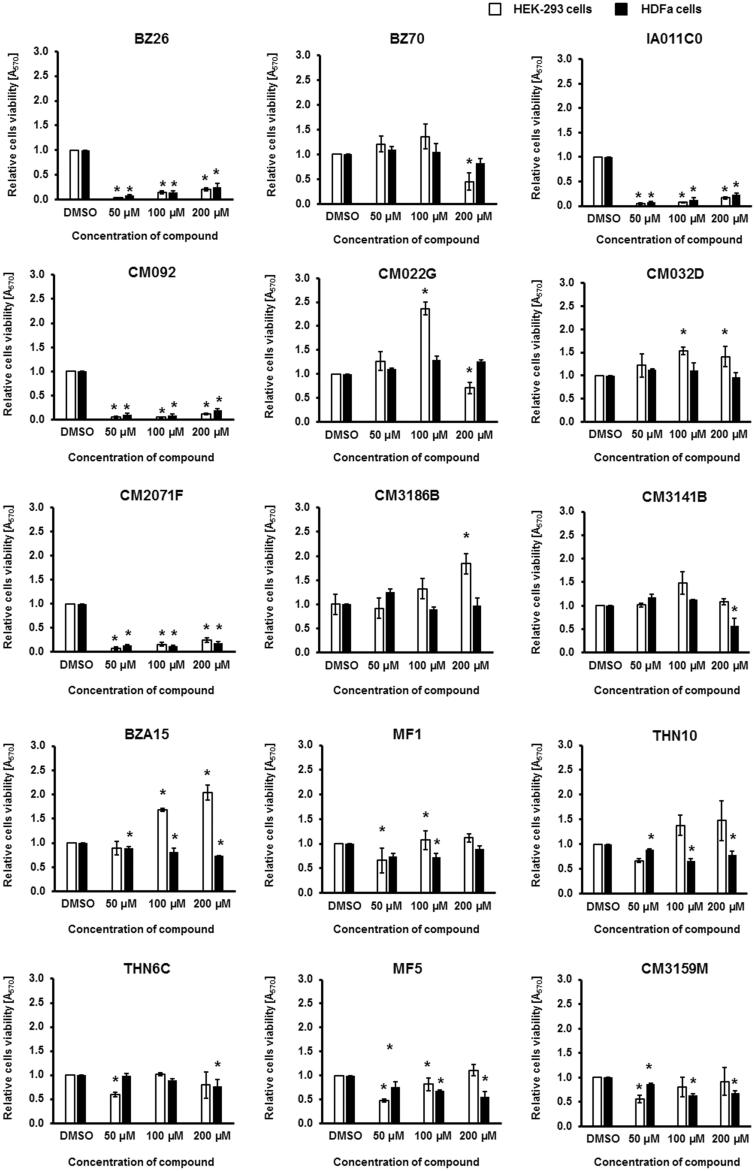
Viability of human HEK-293 and HDFa cells in cultures treated with tested compounds at indicated concentrations for 48 h. Cell viability was tested in the MTT test. Presented results are mean values from three experiments with SD indicated as error bars. The significance of differences between control and cells treated with tested compounds was assessed by the ANOVA test. Differences were marked by asterisks (*) and considered significant when the *p* value was <0.05.

Since any potential therapeutic must be safe for humans, we have tested the selected 15 compounds for toxicity to human cells. Model HEK-293 and HDFa cells were treated with these compounds for 48 h in cultures, and their viability was assessed in the MTT test (Figure 8).

Compounds **BZ26**, **IA011C**, **CM092**, and **CM2071F** were evidently toxic to human cells which precludes their use as therapeutics (Figure 8). Among the rest, there were substances neutral for human cells’ viability or those even stimulating their growth (compounds: **BZ70**, **CM022G**, **CM032D**, **CM3186B**, **CM3141B**, **BZA15**, **MF1**, **THN10**, **THN6C**, **MF5**, and **CM3159B**) (Figure 8).

**Figure 9.**
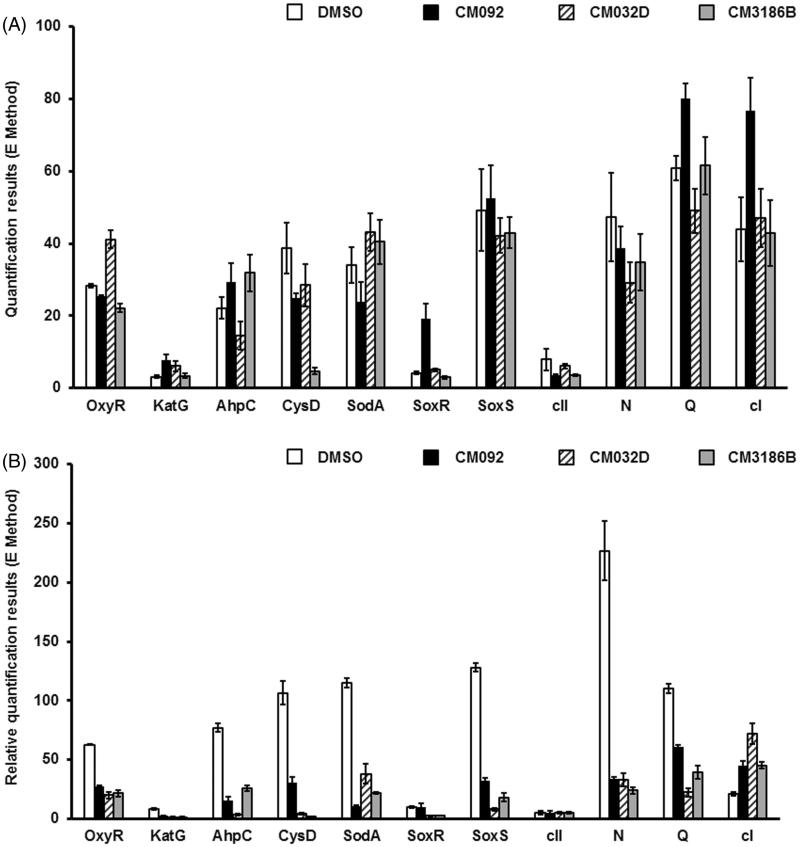
Expression of bacterial genes coding for proteins involved in the oxidative stress response and of selected bacteriophage genes in *E. coli* MG1655 lysogenic with Φ24_B_Δ*stx2*::*cat* either non-treated (A) or after induction with 1 mM H_2_O_2_ (B) in the absence (control experiments) or presence of tested compounds added to final concentration of 0.2 mM. Levels of mRNAs were determined by RT-qPCR. Presented results are mean values from three experiments with SD indicated as error bars.

To learn about molecular mechanisms of impairment of prophage induction and/or phage lytic development, expression of selected phage and host genes was monitored quantitatively by using qRT-PCR. Three compounds belonging to different groups of human cell growth inhibitors (**CM092**), and neutral or weakly stimulating agents (**CM032D**, **CM3186B**), were chosen for further investigations (Figure 9).

In the absence of the prophage inducing agent, the influence of compounds **CM092**, **CM032D**, and **CM3186B** on expression of bacterial genes involved in the oxidative stress response, as well as of bacteriophage genes crucial for either lytic (*N, Q*) or lysogenic (*c*I, *c*II) development was negligible (Figure 9(A)). However, addition of hydrogen peroxide caused activation of the host genes coding for the oxidative stress proteins, and phage genes coding for antitermination proteins (as expected), while having little effects on phage lysogenic proteins (also as expected) (Figure 9(B)). Importantly, simultaneous presence of hydrogen peroxide and any of the tested compounds caused a significant decrease of the efficiency of expression of the oxidative stress genes as well as phage genes (*N, Q*) supporting the lytic development (Figure 9(B)). In contrast to these genes, expression of the gene coding for the cI repressor was enhanced under these conditions. These results suggest that compounds **CM092**, **CM032D**, and **CM3186B** impair expression of bacterial genes involved in the oxidative stress response, or ameliorate the effects of oxidative stress. This leads to prevention of the prophage induction by hydrogen peroxide.

## Discussion and conclusions

Infections by STEC strains are particularly dangerous due to both production of strong toxins (Shiga toxins) by these pathogens and unavailability of the use of antibiotic therapy because these antibacterial compounds may induce Stx prophage leading to enhanced toxin synthesis and release^2^^,^^3^. Stx prophage induction is required for effective expression of *stx* genes and production of Shiga toxins^2^^,^^17^. Therefore, compounds which interfere with prophage induction might be potential anti-STEC agents. Since it was previously demonstrated that oxidative stress conditions are likely responsible for Stx prophage induction in human intestine infected with STEC (summarized in^8^), we asked whether compounds revealing antioxidant features might be effective in inhibition of Stx prophage induction, thus, diminishing pathogenicity of STEC strains.

We have tested 46 compounds, derivatives of carbazole, indazole, triazole, quinolone, ninhydrine, and indenoindole, and after preliminary experiments, 15 of them were chosen for more detailed analysis. These chemicals prevented or alleviated induction of Stx prophages by hydrogen peroxide, thus, revealing an anti-STEC-pathogenicity potential. Importantly, out of 15 tested compounds, eleven had acceptable profiles in the cytotoxicity MTT test, i.e. they did not cause a decrease in viability of two human cell lines (HEK-293 and HDFa). These might be considered for further studies as potential anti-STEC drugs.

Studies on the mechanism of action of the selected compounds, **CM092**, **CM032D**, and **CM3186B**, indicated that in *E. coli* lysogenic with an Stx phage they had generally no significant effects on expression of tested host and phage genes under standard growth conditions. However, after addition of hydrogen peroxide to the bacterial culture, all three tested compounds caused drastic decrease in efficiency of expression of phage genes involved in the lytic development and an increase in expression of the *c*I gene, coding for the phage repressor, responsible for prophage maintenance. These results demonstrate that **CM092**, **CM032D**, and **CM3186B** prevent the prophage induction at the level of expression of specific phage genes. This may arise from interference with the oxidative stress, as in cells treated with hydrogen peroxide, expression of genes involved in the oxidative stress response was significantly less efficient in the presence of the tested compounds. Therefore, the tested compounds apparently reduce the oxidative stress in the presence of hydrogen peroxide, which prevents induction of Stx prophage in *E. coli.*

In conclusion, several compounds tested in this study, particularly **BZ70**, **CM022G**, **CM032D**, **CM3186B**, **CM3141B**, **BZA15**, **MF1**, **THN10**, **THN6C**, **MF5**, **CM3159B**, might be considered as potential anti-STEC drugs, and qualified for further studies. At least some of them, **CM092**, **CM032D**, and **CM3186B**, act by reduction of the oxidative stress, changes in gene expression and prevention of Stx prophage induction.

## Supplementary Material

IENZ_1444610_Supplementary_Material.pdf
