# Supplementary material for: Inhibition of Shiga toxin-converting bacteriophage development by novel antioxidant compounds
Source: J Enzyme Inhib Med Chem. 2018 Mar 14;33(1):639–50. doi: 10.1080/14756366.2018.1444610 (PMC6009899; doi:10.1080/14756366.2018.1444610)
Supplement: IENZ_1444610_Supplementary_Material.pdf [file IENZ_A_1444610_SM5979.pdf]

Supplementary Information

Inhibition of Shiga toxin-converting bacteriophage development by novel antioxidant compounds

Sylwia Bloch[a,#], Bożena Nejman-Falenczyk[a,#], Karolina Pierzynowska[a], Ewa Piotrowska[a], Alicja Węgrzyn[b], Christelle Marminon[c], Zouhair Bouaziz[c], Pascal Nebois[c], Joachim Jose[d], Marc Le Borgne[c], Luciano Saso[e], Grzegorz Węgrzyn[a,*]

[a] *Department of Molecular Biology, University of Gdansk, Wita Stwosza 59, 80-308 Gdansk, Poland*

[b] *Institute of Biochemistry and Biophysics, Polish Academy of Sciences, Pawińskiego 5A, 02-106 Warsaw, Poland*

[c] *Université de Lyon, Université Claude Bernard Lyon 1, Faculté de Pharmacie - ISPB, EA 4446 Bioactive Molecules and Medicinal Chemistry, SFR Santé Lyon-Est CNRS UMS3453-INSERM US7, 8 avenue Rockefeller, F-69373, Lyon Cedex 8, France*

[d] *Institut für Pharmazeutische und Medizinische Chemie, PharmaCampus, Westfälische Wilhelms-Universität Münster, Corrensstraße 48, 48149 Münster, Germany*

[e] *Sapienza University, Department of Physiology and Pharmacology "Vittorio Erspamer", P.le Aldo Moro 5, 00185, Rome, Italy*

# These authors contributed equally to this work.

* Corresponding author: Dr. Grzegorz Węgrzyn, Department of Molecular Biology, University of Gdansk, Wita Stwosza 59, 80-308 Gdansk, Poland.
Tel. +48 58 523 6024, Fax: +48 58 523 5001, e-mail: grzegorz.wegrzyn@biol.ug.edu.pl

Chemical structures of compounds tested in this report

Forty-six compounds were tested to determine their effects on the lysogenic strain growth. The best fifteen compounds were selected for further biological exploration.

The structures of all compounds tested are presented in Figures S1-S4.

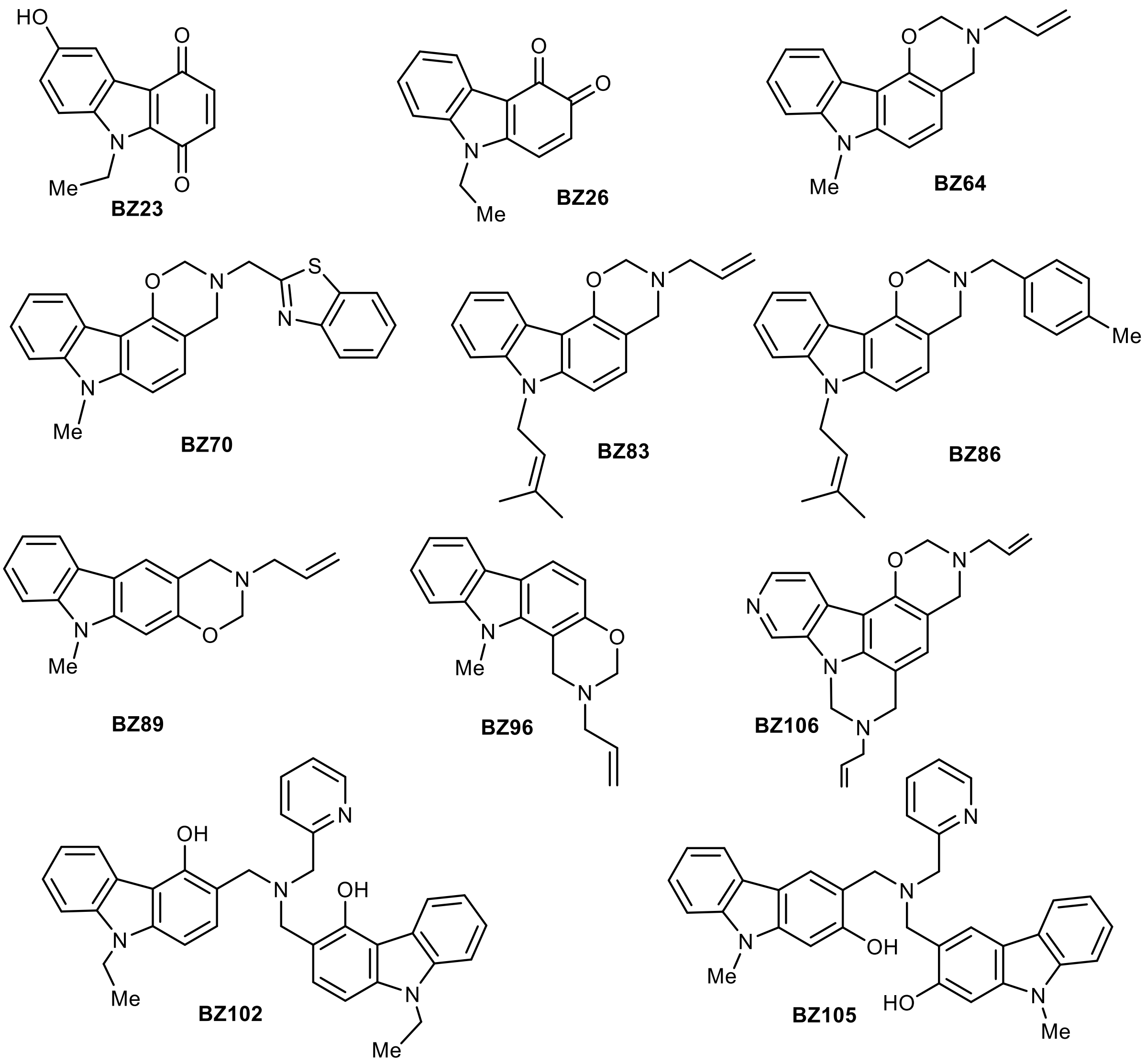

Figure S1. Structure of investigated carbazole derivatives (44,45).

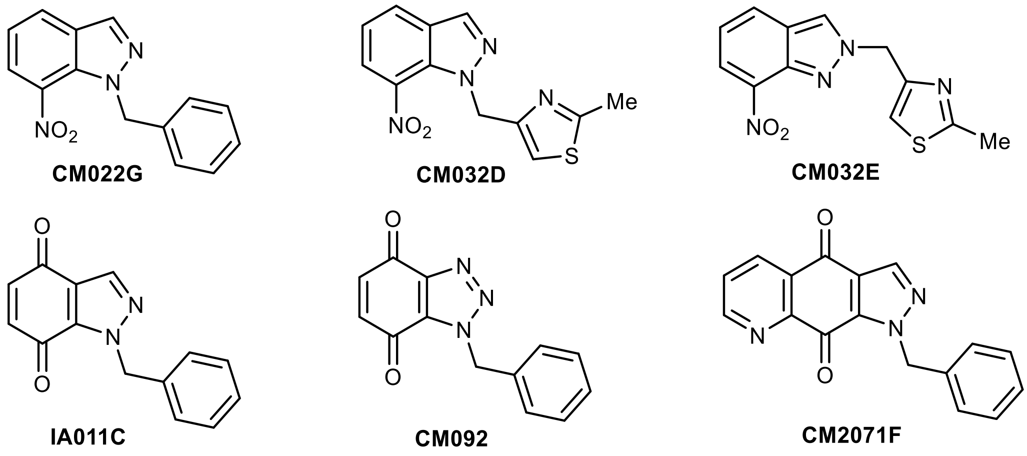

Figure S2. Structure of investigated indazole, triazole and quinoline derivatives (40-42).

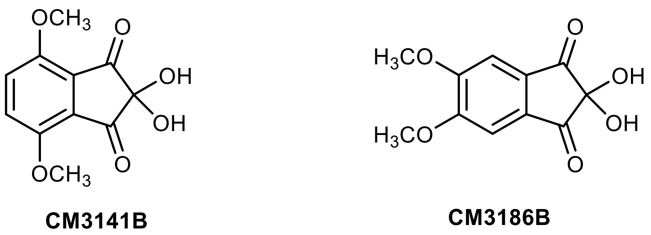

Figure S3. Structure of investigated ninhydrine derivatives (47).

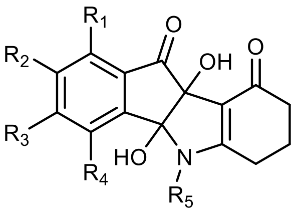

R_1, R_2, R_3, R_4 = H; R_5 = CH_2CH_2OMe: **AR09**
R_1, R_2, R_3, R_4 = H; R_5 = CH_2CH_2COOH: **BZA23**
R_1, R_2, R_3, R_4 = H; R_5 = $CH(CH_3)_2$: **AR02**
R_1 = Br; R_2, R_3, R_4 = H; R_5 = $CH(CH_3)_2$: **CM3116A**
R_1, R_4 = OMe; R_1, R_3 = H; R_5 = $CH(CH_3)_2$: **CM3159A**
R_1, R_4 = H; R_2, R_3 = OMe; R_5 = $CH(CH_3)_2$: **MF4**
R_1, R_2, R_3, R_4 = H; R_5 = CH_2Ph: **BZA15**

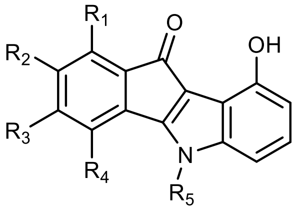

R_1, R_2, R_3, R_4 = H; R_5 = CH_2CH_2OMe: **AR27**
R_1, R_2, R_3, R_4 = H; R_5 = CH_2CH_2(*ortho*-OMe)Ph: **MQ4**
R_1, R_2, R_3, R_4 = H; R_5 = CH_2CH_2(*meta*-OMe)Ph: **BZA37**
R_1, R_2, R_3, R_4 = H; R_5 = CH_2CH_2(*para*-OMe)Ph: **MQ8**
R_1 = OH; R_2, R_3, R_4 = H; R_5 = $CH(CH_3)_2$: **CM4017A**
R_1 = Br; R_2, R_3, R_4 = H; R_5 = $CH(CH_3)_2$: **CM3130B**
R_1, R_4 = OMe; R_2, R_3 = H; R_5 = $CH(CH_3)_2$: **CM4016A**
R_1, R_4 = H; R_2, R_3 = OMe; R_5 = $CH(CH_3)_2$: **MF6**

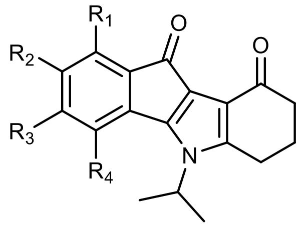

R_1 = OH; R_2, R_3, R_4 = H: **MF27A**
R_1 = Me; R_2, R_3 , R_4 = H: **CM3146B**
R_1 = Br; R_2, R_3 , R_4 = H: **CM3116C**
R_1 = CN; R_2, R_3 , R_4 = H: **CM3129A**
R_1, R_2, R_3 = H; R_4 = OMe: **CM3072B**
R_1 = OMe; R_2, R_3, R_4 = H: **THN10**
R_1, R_2, R_3 = H; R_4 = OH: **THN6C**
R_1 = NH_2; R_2, R_3, R_4 = H: **MF1**
R_1, R_4 = H; R_2, R_3 = OMe: **MF5**
R_1, R_4 = OMe; R_2, R_3 = H: **CM3159B**

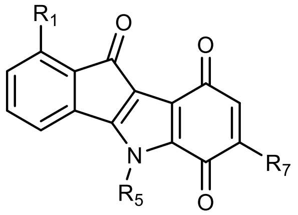

R_1 = H; R_5 = CH_2CH_2Ph; R_7 = Me: **SiA3**
R_1 = NO_2; R_5 = $CH(CH_3)_2$; R_7 = H: **CM3112B**

Figure S4. Structures of the investigated indenoindole derivatives (47-49,51).

Complementary reference for the access of compounds **MQ4**, **BZA37**, **MQ8**, **SiA3**:

51. Gozzi GJ, Bouaziz Z, Winter E, Daflon-Yunes N, Honorat M, Guragossian N, Marminon C, Valdameri G, Bollacke A, Guillon J, Pinaud N, Marchivie M, Cadena SM, Jose J, Le Borgne M, Di Pietro A. Phenolic indeno[1,2-*b*]indoles as ABCG2-selective potent and nontoxic inhibitors stimulating basal ATPase activity. Drug Des Devel Ther 2015;9:3481-95.

LC/HRMS data of the new indenoindole derivatives

Twenty-seven indenoindole derivatives were used in our biological investigation. Eight indenoindole derivatives are already published (**AR02**, **BZA15**, **MF27A**, **THN6C**, **MQ4**, **BZA37**, **MQ8**, **SiA3**) (47-49,51). Twelve indenoindole derivatives are currently evaluated on different targets and will be published shortly (**AR09**, **BZA23**, **CM3116A**, **CM3146B**, **CM3116C**, **CM3129A**, **AR27**, **CM4017A**, **CM3130B**, **CM4016A**, **MF6**, **CM3112B**). The last seven indenoindole derivatives are described in this paper (**CM3159A**, **MF4**, **THN10**, **CM3072B**, **CM3159B**, **MF5**, **MF1**).

The purity of the indenoindole derivatives was determined by uHPLC/MS on an Agilent 1290 system using a Agilent 1290 Infinity ZORBAX Eclipse Plus C18 column (2.1 mm × 50 mm, 1.8 µm particle size) with a gradient mobile phase of H_2O/CH_3CN (90:10, v/v) with 0.1% of formic acid to H_2O/CH_3CN (10:90, v/v) with 0.1% of formic acid, at a flow rate of 0.5 mL/min, with UV monitoring at the wavelength of 254 nm. A run time of 10 min was applied.

High Resolution Mass Spectroscopy (HRMS) spectra were recorded on a Bruker MicrOTOFQ II 10231 Spectrometer at the "Centre Commun de Spectrométrie de Masse" of the University Lyon 1. The acquisition parameters are described for each record spectrum.

On pages S6-S43, all LC/HRMS data of the nineteen new indenoindole derivatives are given.

Twelve indenoindole derivatives are currently evaluated on different targets and will be published shortly (**AR09**, **BZA23**, **CM3116A**, **CM3146B**, **CM3116C**, **CM3129A**, **AR27**, **CM4017A**, **CM3130B**, **CM4016A**, **MF6**, **CM3112B**). All LC/HRMS data are given.

AR09, MW=329.35

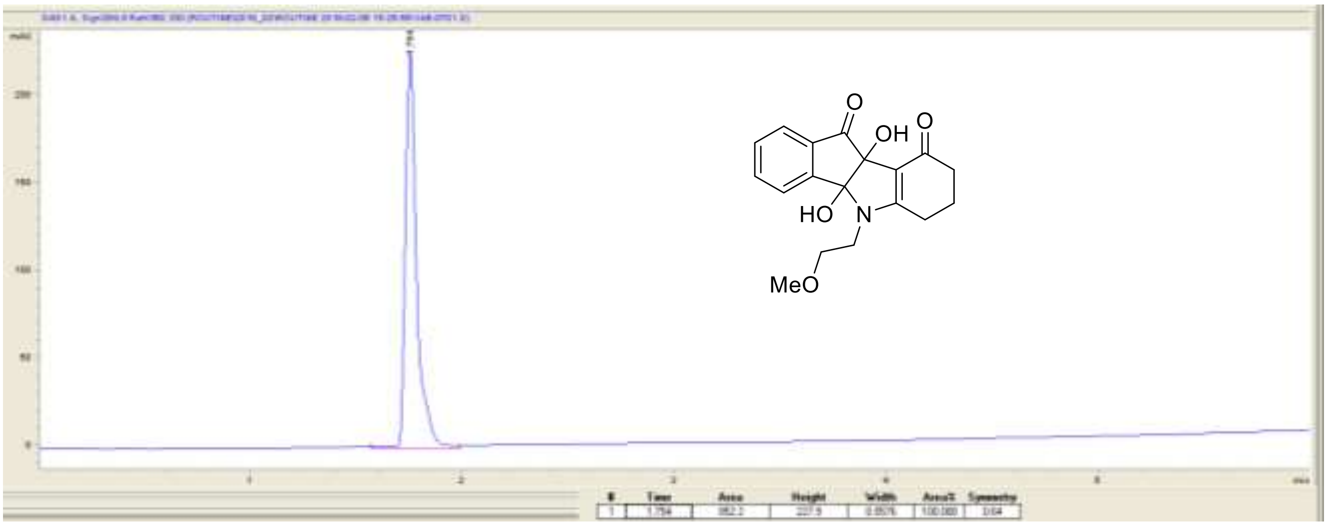

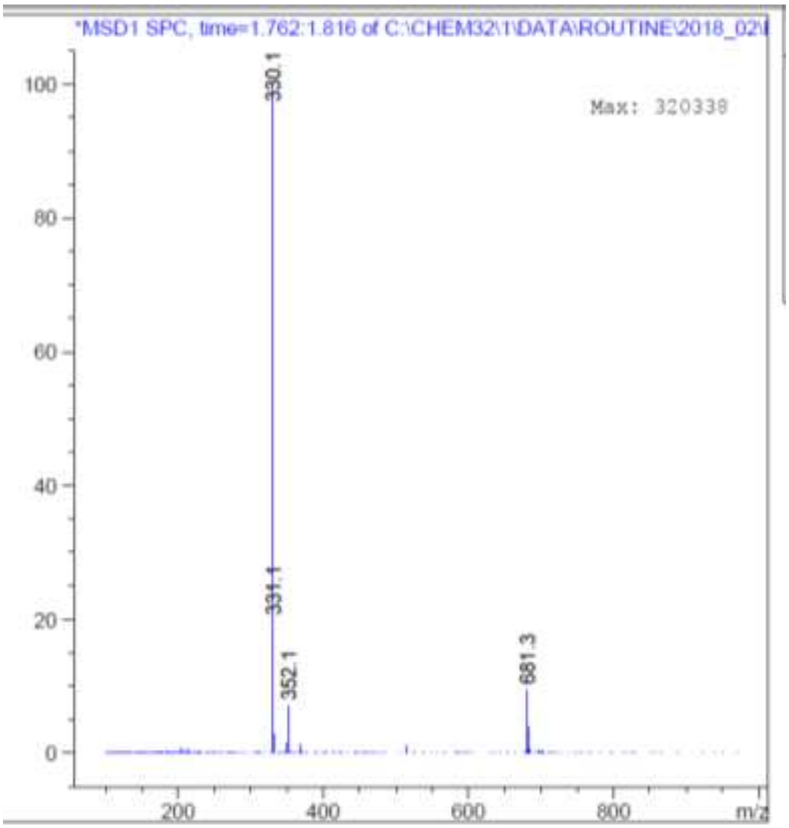

Analysis Info

Analysis Name	QTOF_100503_05_AR09.d		
Method	ms_fia_tl_50_1000_pos_150ul.min_ccsm.m	Acquisition Date	5/3/2010 11:34:02 AM
Comment		Instrument / Ser#	micrOTOF-Q II 10231

Acquisition Parameter

Source Type	ESI	Ion Polarity	Positive	Set Nebulizer	24.7 psi
Focus	Not active	Set Capillary	4500 V	Set Dry Heater	200 °C
Scan Begin	50 m/z	Set End Plate Offset	-500 V	Set Dry Gas	7.0 l/min
Scan End	1000 m/z	Set Collision Cell RF	100.0 Vpp	Set Divert Valve	Waste

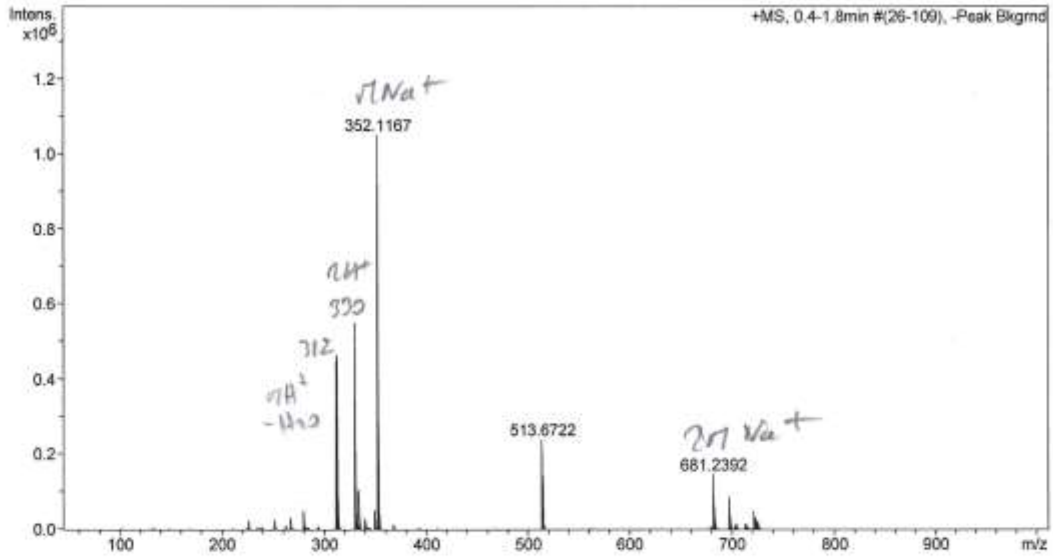

Meas. m/z	Formula	m/z	err [ppm]	mSigma
330.1334	C 18 H 20 N O 5	330.1336	0.6	9.2
352.1167	C 18 H 19 N Na O 5	352.1155	-3.2	43.9

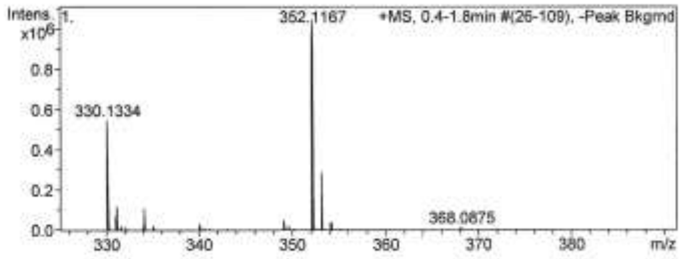

BZA23, MW=343.11

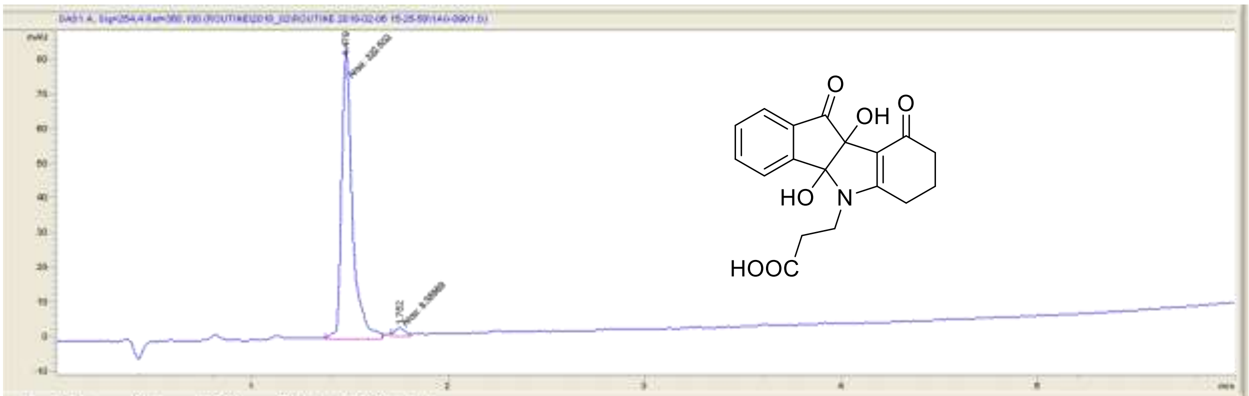

#	Time	Area	Height	Width	Area%	Symmetry
1	1.478	322.7	85.1	0.0632	97.466	0.689
2	1.752	8.4	2.4	0.0694	2.534	0.834

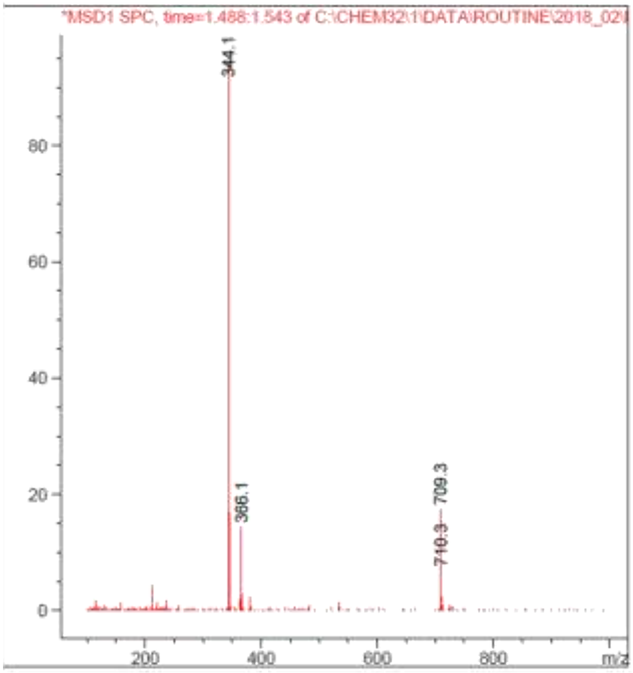

Analysis Info

Analysis Name	QTOF_120712_04_BZA23.d		
Method	MS_inf_TL_50_1000_Pos_CCSM_2.m	Acquisition Date	7/12/2012 3:38:25 PM
Comment		Instrument / Ser#	micrOTOF-Q II 10231

Acquisition Parameter

Source Type	ESI	Ion Polarity	Positive	Set Nebulizer	0.4 Bar
Focus	Active	Set Capillary	3500 V	Set Dry Heater	200 °C
Scan Begin	50 m/z	Set End Plate Offset	-500 V	Set Dry Gas	4.0 l/min
Scan End	1000 m/z	Set Collision Cell RF	50.0 Vpp	Set Divert Valve	Waste

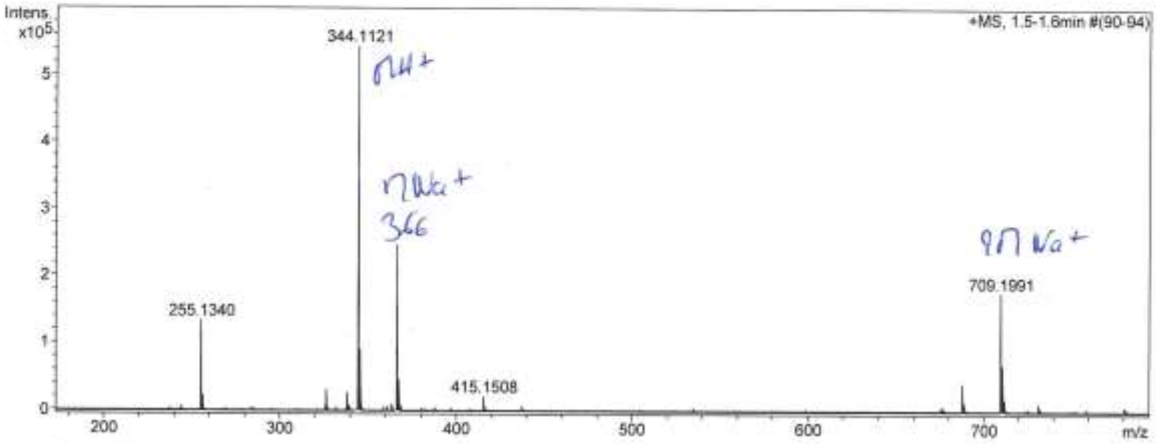

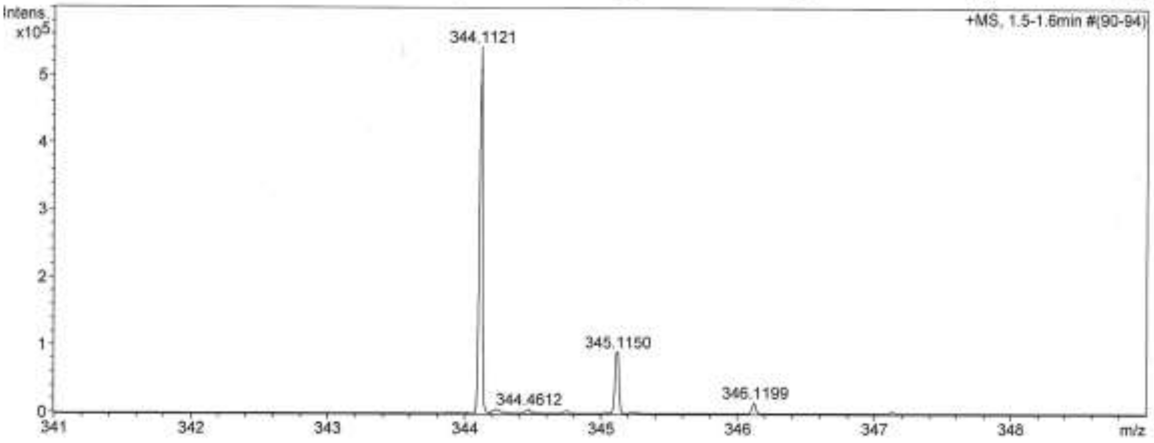

Meas. m/z	Formula	m/z	err [ppm]	mSigma
344.1121	C 18 H 18 N O 6	344.1129	2.3	17.5

CM3116A, MW=391.04

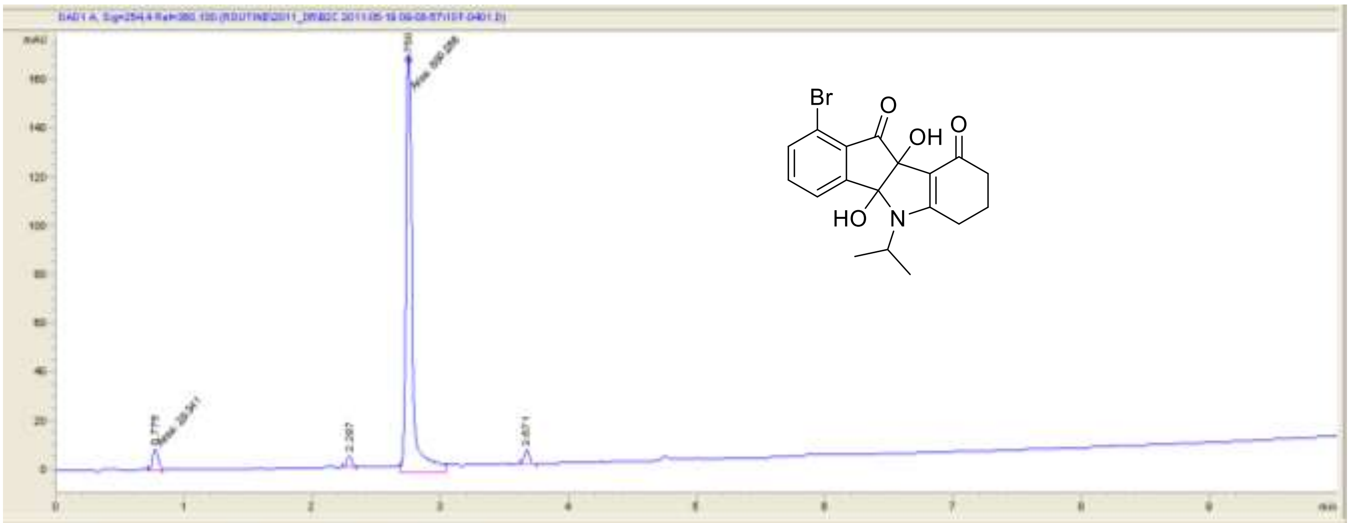

#	Time	Area	Height	Width	Area%	Symmetry
1	0.776	29.3	9	0.0544	4.455	0.905
2	2.287	12.7	4.5	0.0448	1.931	0.946
3	2.75	600.3	174.8	0.0572	91.139	0.702
4	3.671	16.3	5.6	0.046	2.475	0.938

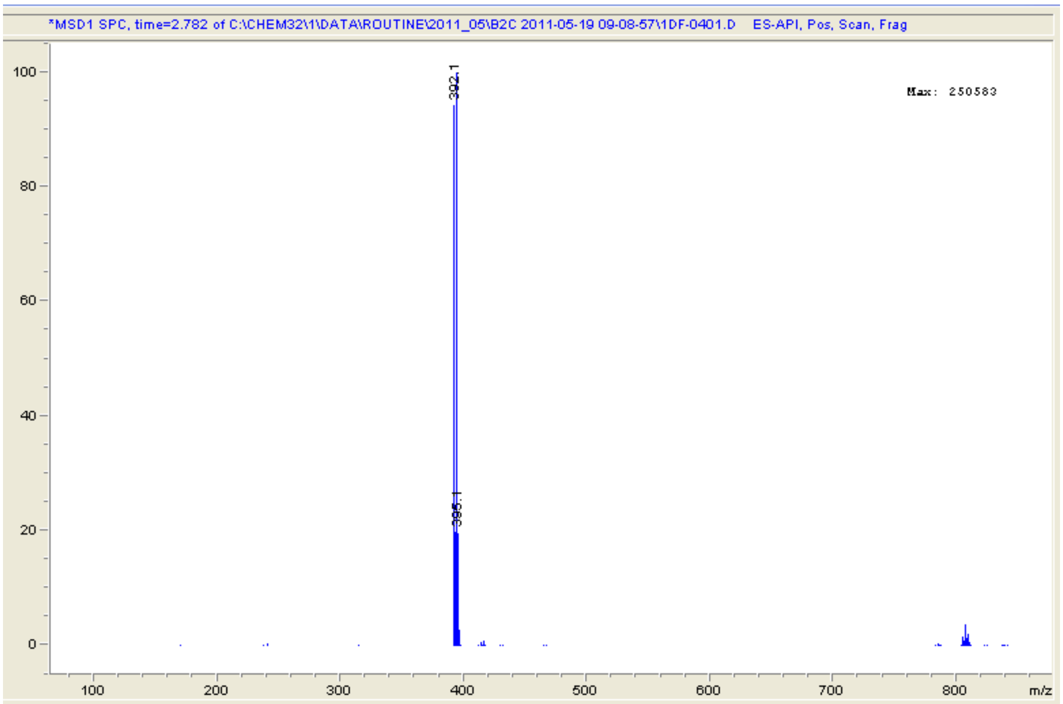

Analysis Info

Analysis Name	QTOF_140418_15_CM3116A.d		
Method	MS_inf_TL_50_1000_Pos_CCSM_2.m	Acquisition Date	4/18/2014 2:26:19 PM
Comment		Instrument / Ser#	micrOTOF-Q II 10231

Acquisition Parameter

Source Type	ESI	Ion Polarity	Positive	Set Nebulizer	0.6 Bar
Focus	Not active	Set Capillary	1500 V	Set Dry Heater	200 °C
Scan Begin	50 m/z	Set End Plate Offset	-500 V	Set Dry Gas	4.0 l/min
Scan End	1000 m/z	Set Collision Cell RF	10.0 Vpp	Set Divert Valve	Waste

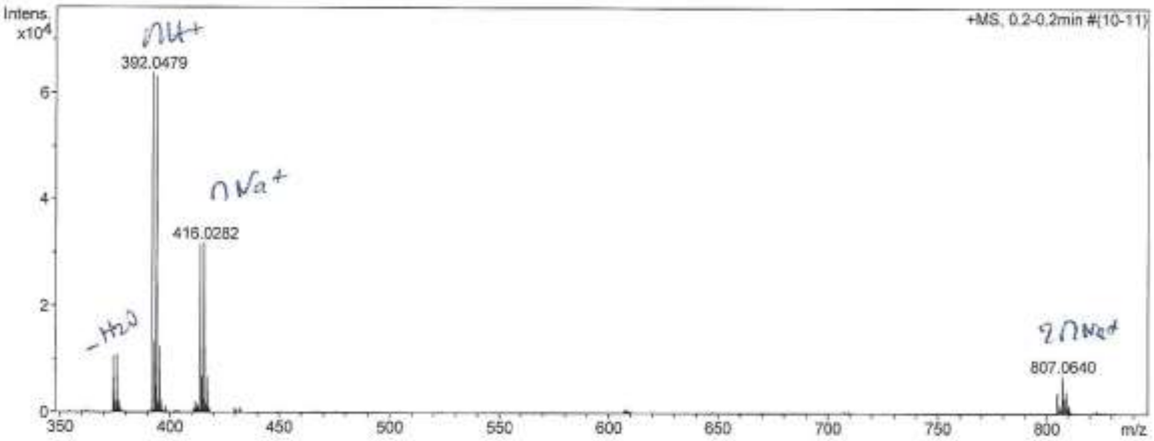

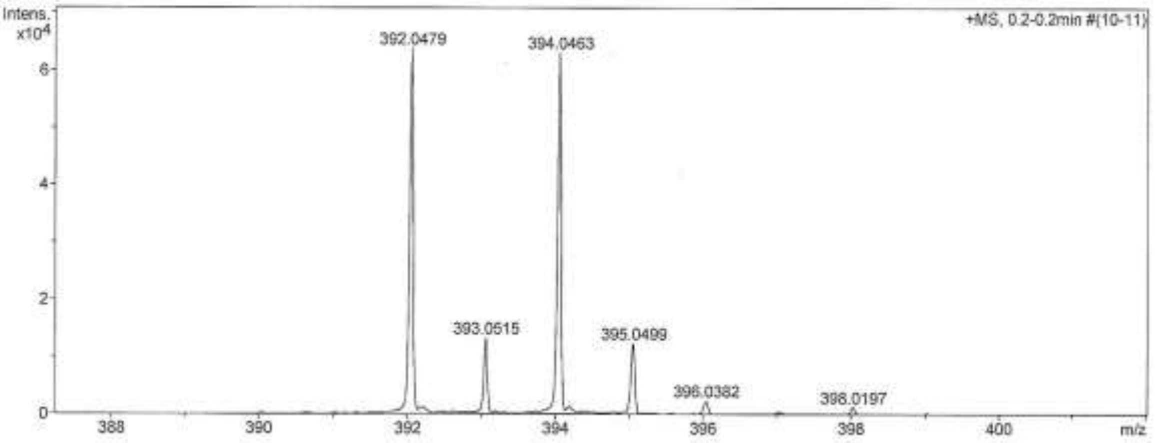

Meas. m/z	Formula	m/z	err [ppm]	mSigma
392.0479	C 18 H 19 Br N O 4	392.0492	3.2	7.8

CM3146B, MW=293.14

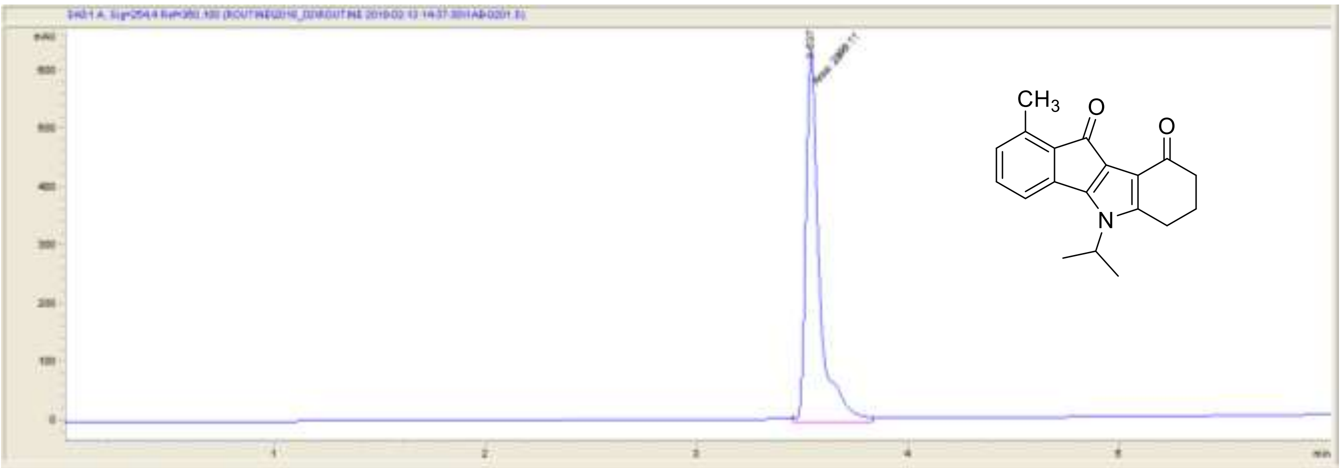

#	Time	Area	Height	Width	Area%	Symmetry
1	3.537	2899.1	644.3	0.075	100.000	0.57

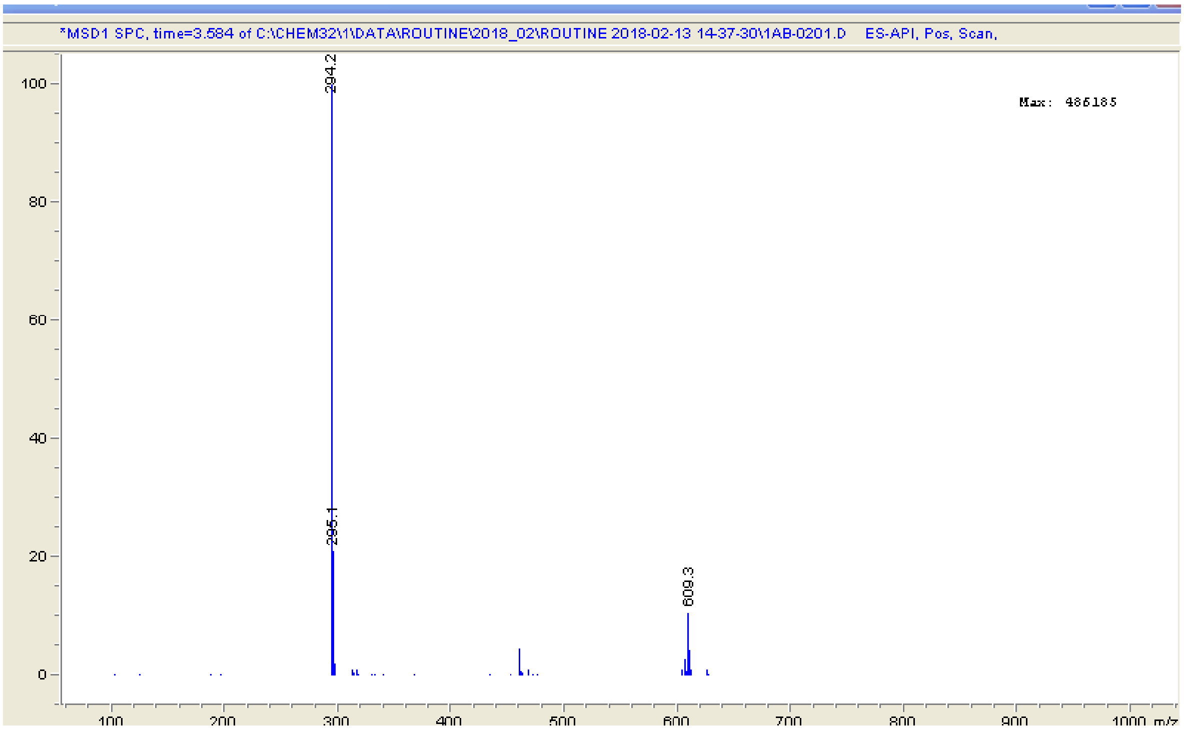

Analysis Info

Analysis Name	QTOF_140418_14_CM3146B.d		
Method	MS_inf_TL_50_1000_Pos_CCSM_2.m	Acquisition Date	4/18/2014 2:22:43 PM
Comment		Instrument / Ser#	micrOTOF-Q II 10231

Acquisition Parameter

Source Type	ESI	Ion Polarity	Positive	Set Nebulizer	0.6 Bar
Focus	Not active	Set Capillary	1500 V	Set Dry Heater	200 °C
Scan Begin	50 m/z	Set End Plate Offset	-500 V	Set Dry Gas	4.0 l/min
Scan End	1000 m/z	Set Collision Cell RF	10.0 Vpp	Set Divert Valve	Waste

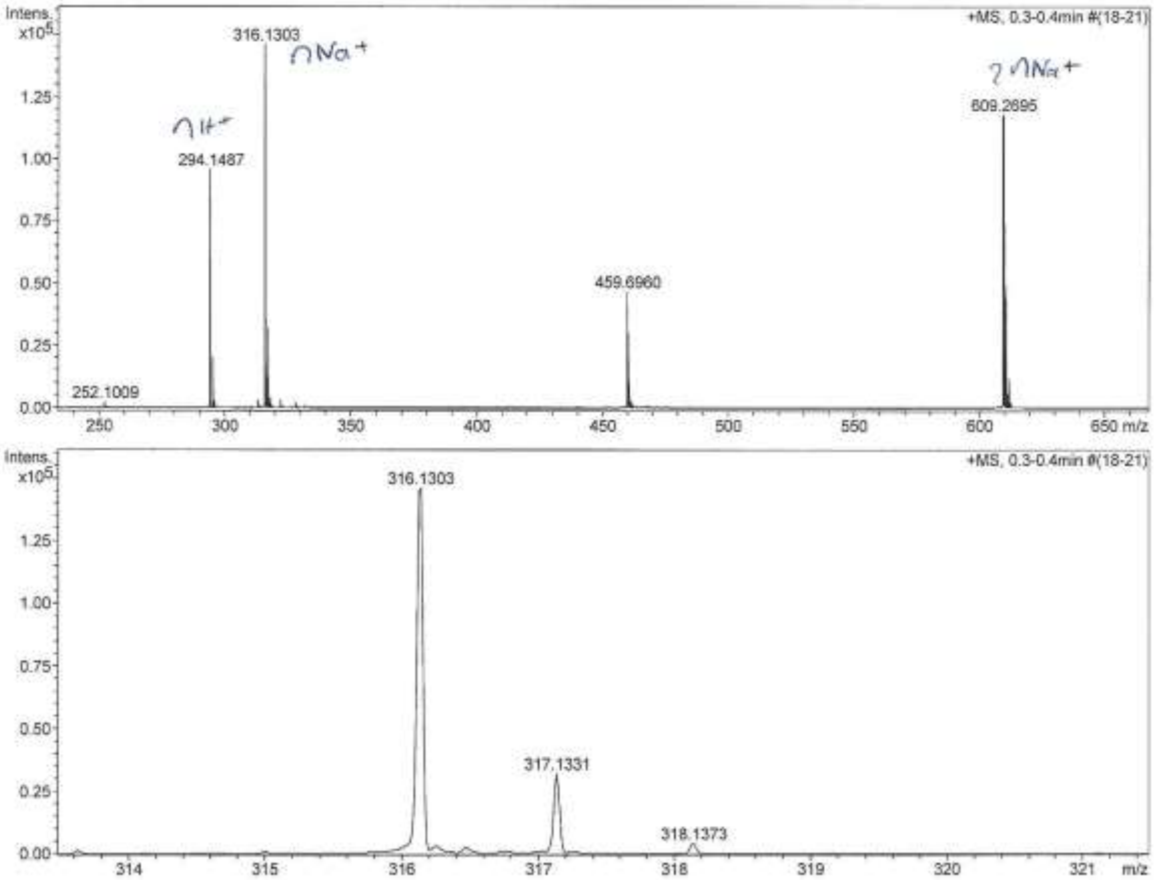

Meas. m/z	Formula	m/z	err [ppm]	mSigma
316.1303	C 19 H 19 N Na O 2	316.1308	1.5	6.7

CM3116C, MW=357.04

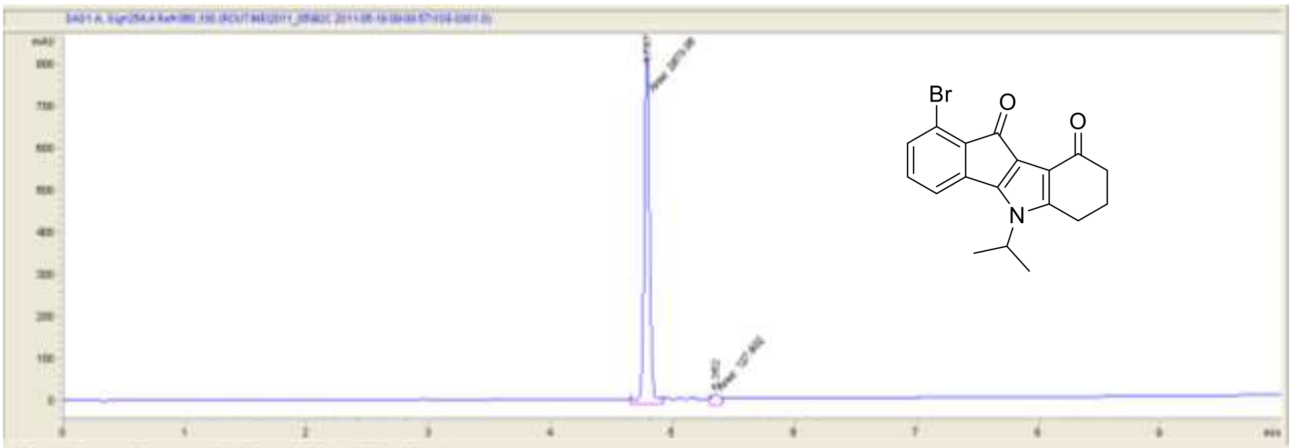

#	Time	Area	Height	Width	Area%	Symmetry
1	4.787	2675.1	842.3	0.0529	95.440	0.959
2	5.352	127.8	26.9	0.0792	4.560	0.875

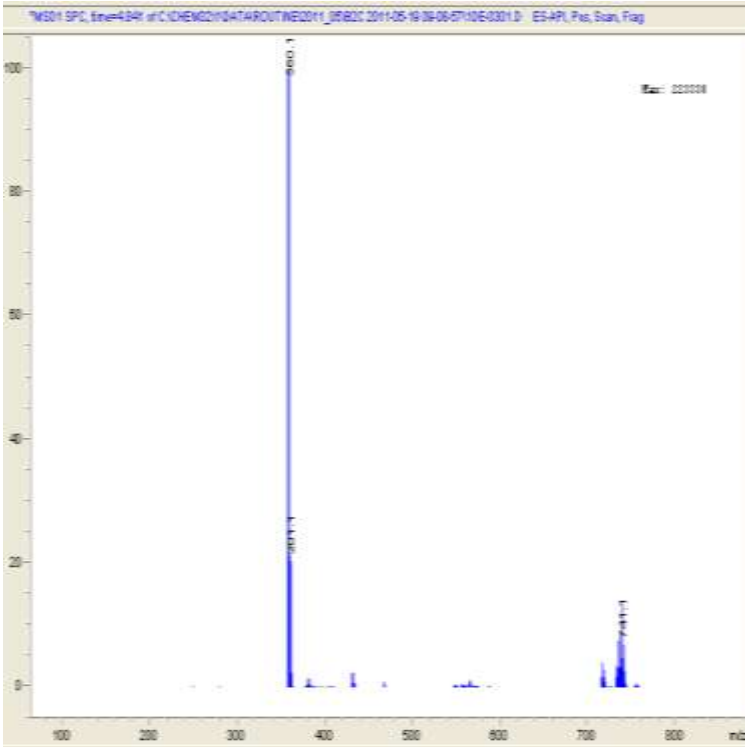

Analysis Info

Analysis Name	QTOF_140418_16_CM3116C.d		
Method	MS_inf_TL_50_1000_Pos_CCSM_2.m	Acquisition Date	4/18/2014 2:32:06 PM
Comment		Instrument / Ser#	micrOTOF-Q II 10231

Acquisition Parameter

Source Type	ESI	Ion Polarity	Positive	Set Nebulizer	0.6 Bar
Focus	Not active	Set Capillary	1500 V	Set Dry Heater	200 °C
Scan Begin	50 m/z	Set End Plate Offset	-500 V	Set Dry Gas	4.0 l/min
Scan End	1000 m/z	Set Collision Cell RF	10.0 Vpp	Set Divert Valve	Waste

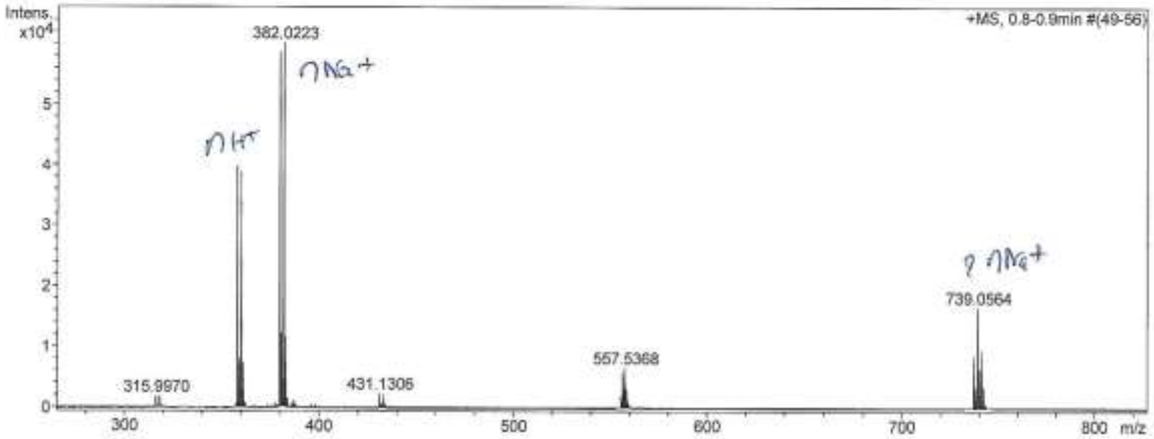

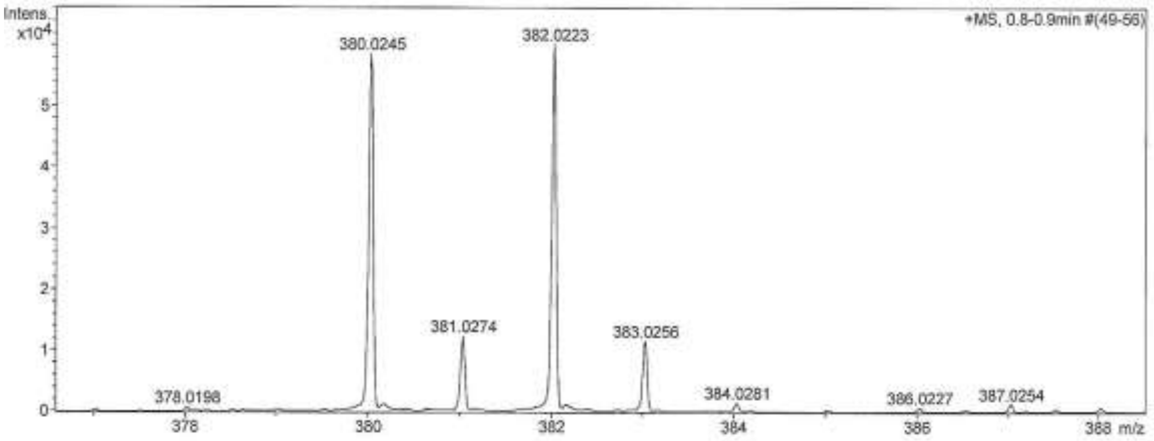

Meas. m/z	Formula	m/z	err [ppm]	mSigma
380.0245	C 18 H 16 Br N Na O 2	380.0257	3.2	12.5

CM3129A, MW=304.12

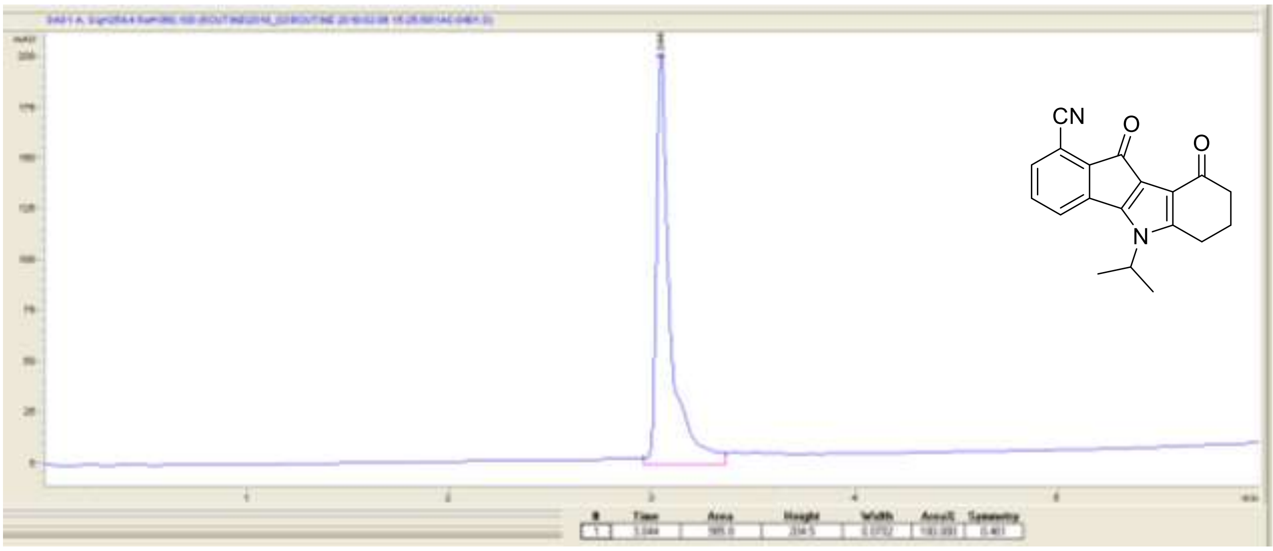

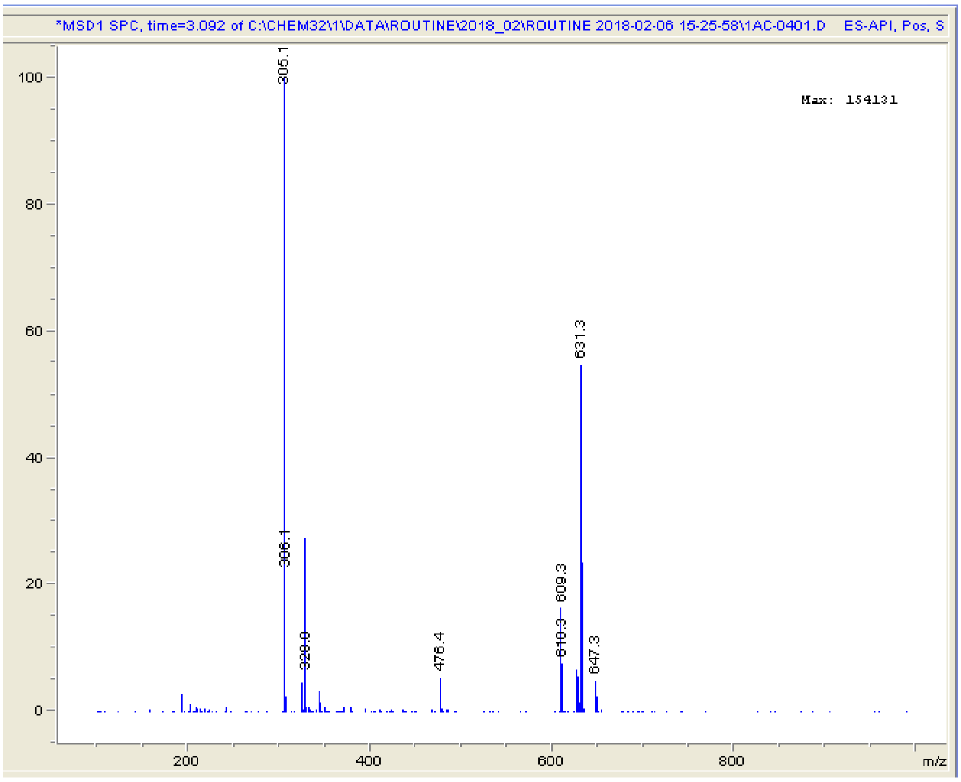

Analysis Info

Analysis Name	QTOF180213_07_CM3129A.d		
Method	2016_03_17_Infusion_50-1000_pos.m	Acquisition Date	2/13/2018 9:57:31 AM
Comment		Instrument / Ser#	micrOTOF-Q 228888.10231

Acquisition Parameter

Source Type	ESI	Ion Polarity	Positive	Set Nebulizer	0.4 Bar
Focus	Active	Set Capillary	1000 V	Set Dry Heater	200 °C
Scan Begin	50 m/z	Set End Plate Offset	-500 V	Set Dry Gas	4.0 l/min
Scan End	1000 m/z	Set Collision Cell RF	400.0 Vpp	Set Divert Valve	Waste

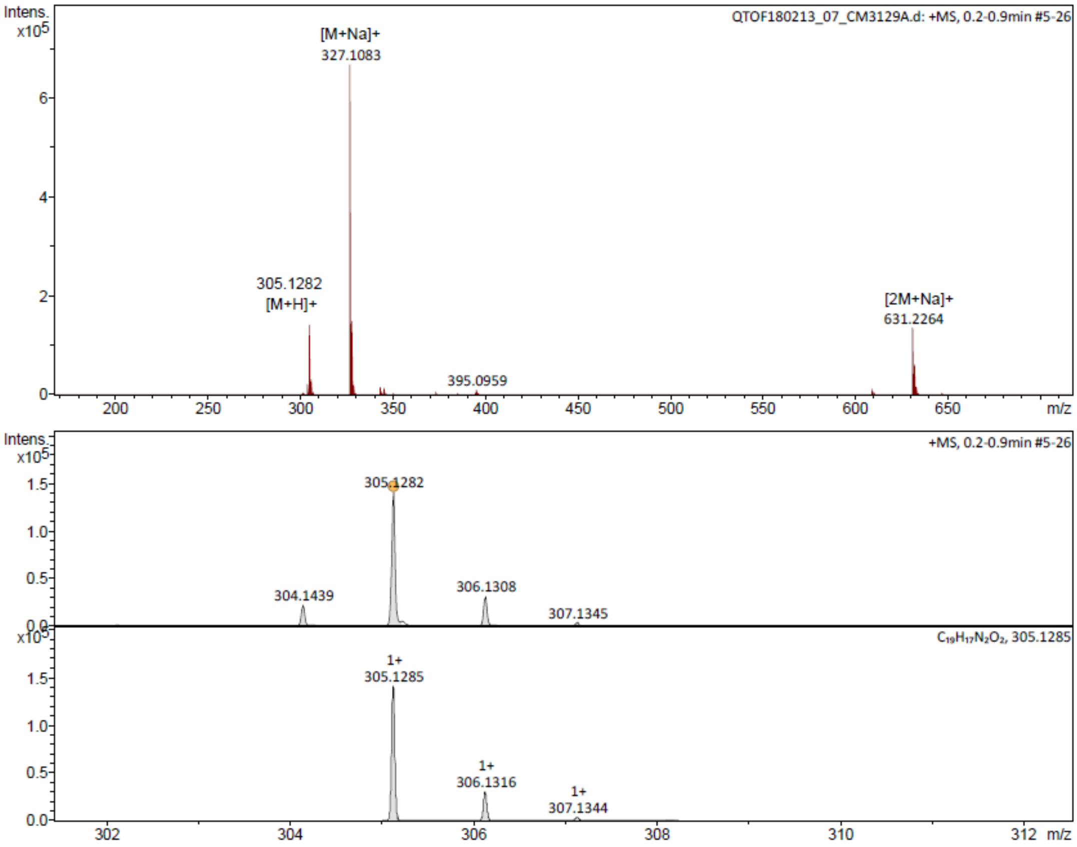

Meas. m/z	Ion Formula	m/z	err [ppm]	mSigma
305.1282	C19H17N2O2	305.1285	0.9	4.2

AR27, MW=293.32

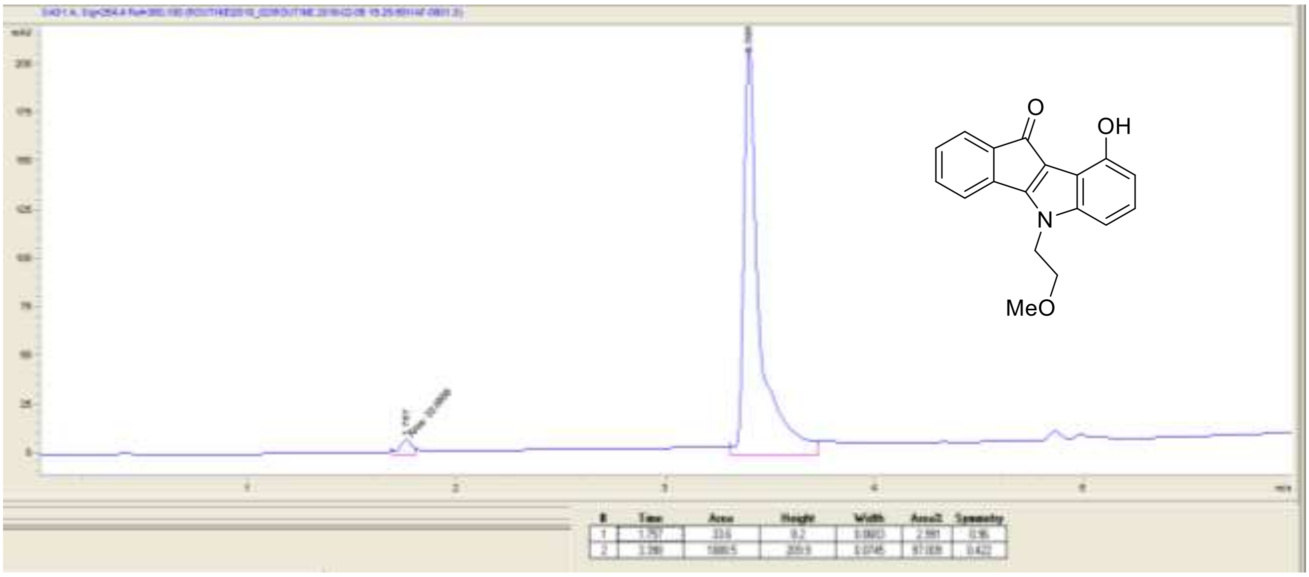

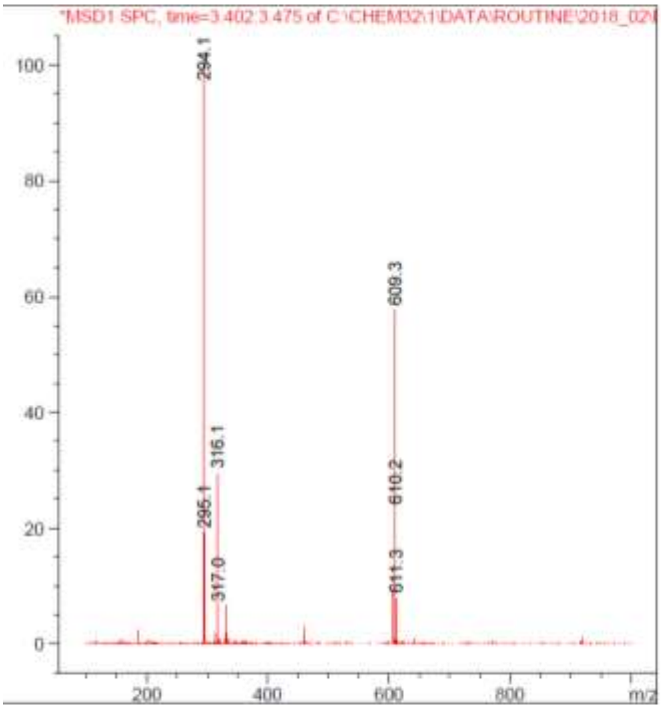

Analysis Info

Analysis Name	QTOF_100609_02_AR27.d		
Method	ms_fia_tl_50_1000_pos_150ul.min_ccsm.m	Acquisition Date	6/9/2010 9:57:54 AM
Comment		Instrument / Ser#	micrOTOF-Q II 10231

Acquisition Parameter

Source Type	ESI	Ion Polarity	Positive	Set Nebulizer	1.7 Bar
Focus	Not active	Set Capillary	4500 V	Set Dry Heater	200 °C
Scan Begin	50 m/z	Set End Plate Offset	-500 V	Set Dry Gas	7.0 l/min
Scan End	1000 m/z	Set Collision Cell RF	100.0 Vpp	Set Divert Valve	Waste

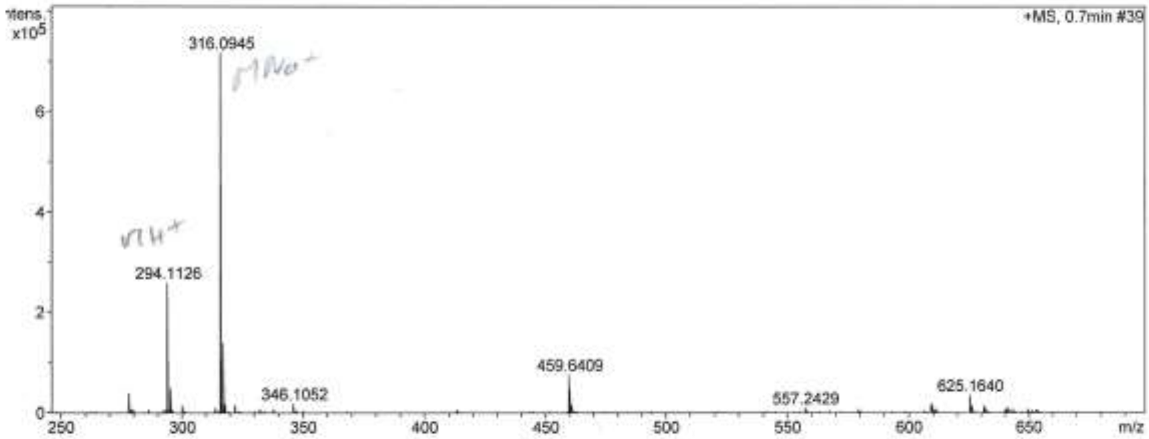

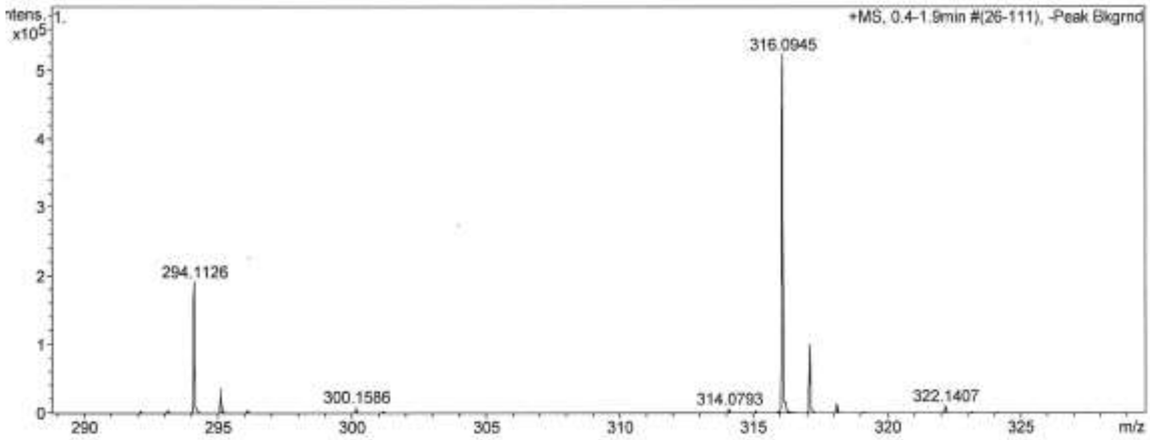

Meas. m/z	Formula	m/z	err [ppm]	mSigma
294.1126	C 18 H 16 N O 3	294.1125	-0.4	4.3
316.0945	C 18 H 15 N Na O 3	316.0944	-0.1	2.9

CM4017A, MW=293.11

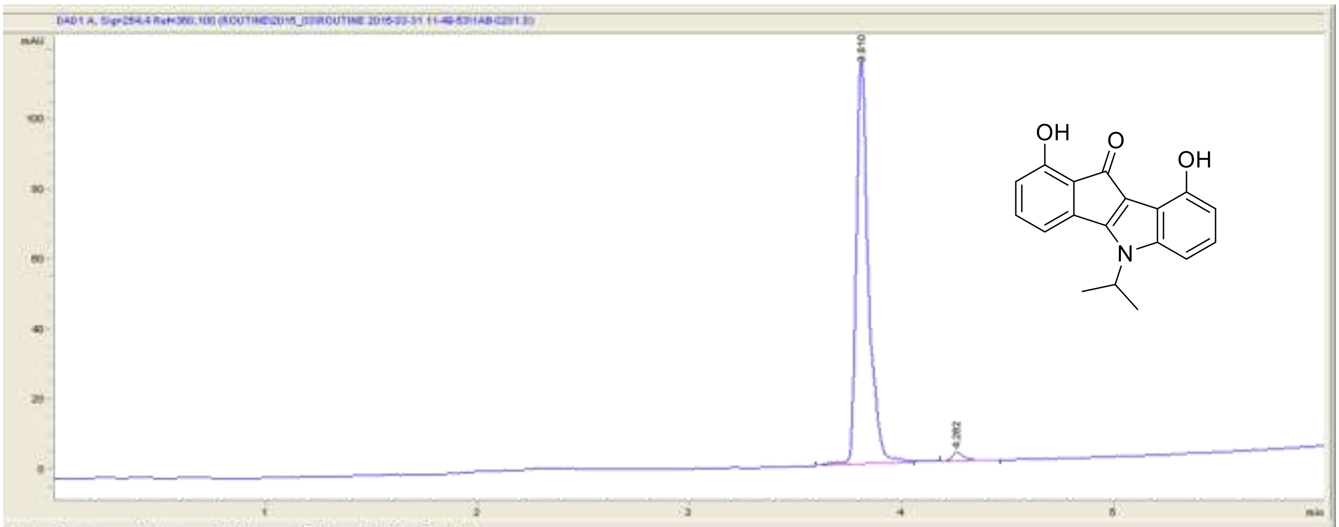

#	Time	Area	Height	Width	Area%	Symmetry
1	3.81	466.1	116.9	0.0605	97.527	0.639
2	4.262	11.8	2.7	0.0628	2.473	0.698

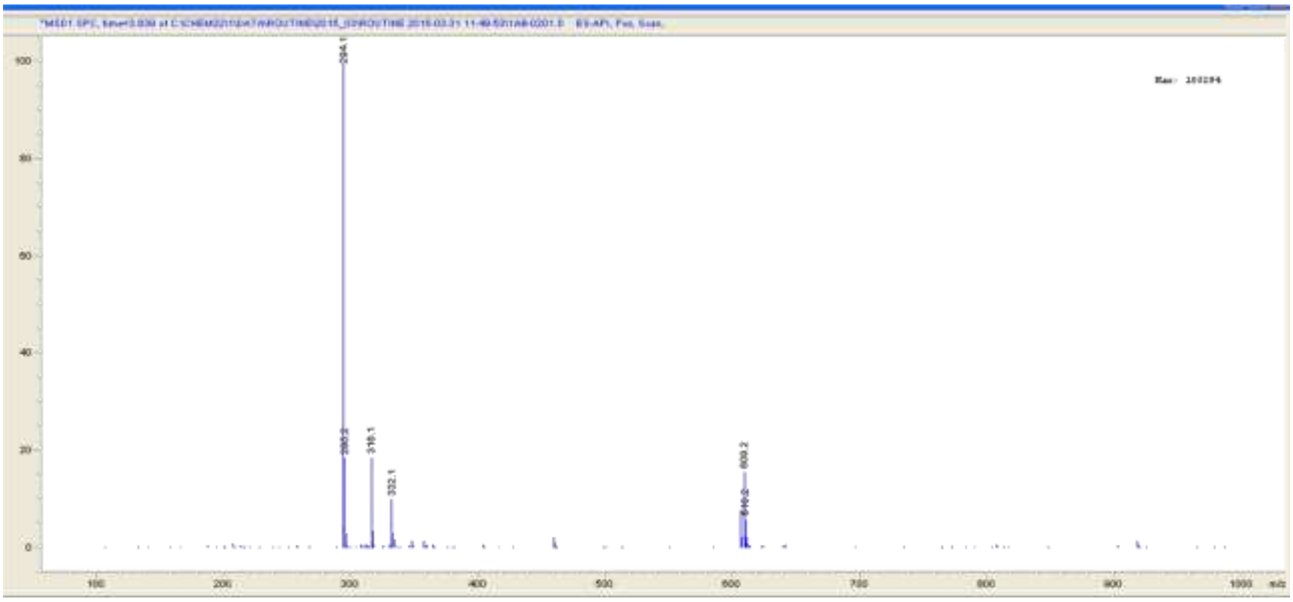

Analysis Info

Analysis Name	QTOF180213_05_CM4017A.d		
Method	2016_03_17_Infusion_50-1000_pos.m	Acquisition Date	2/13/2018 9:18:38 AM
Comment		Instrument / Ser#	microTOF-Q 228888.10231

Acquisition Parameter

Source Type	ESI	Ion Polarity	Positive	Set Nebulizer	0.4 Bar
Focus	Active	Set Capillary	1500 V	Set Dry Heater	200 °C
Scan Begin	50 m/z	Set End Plate Offset	-500 V	Set Dry Gas	4.0 l/min
Scan End	1000 m/z	Set Collision Cell RF	400.0 Vpp	Set Divert Valve	Waste

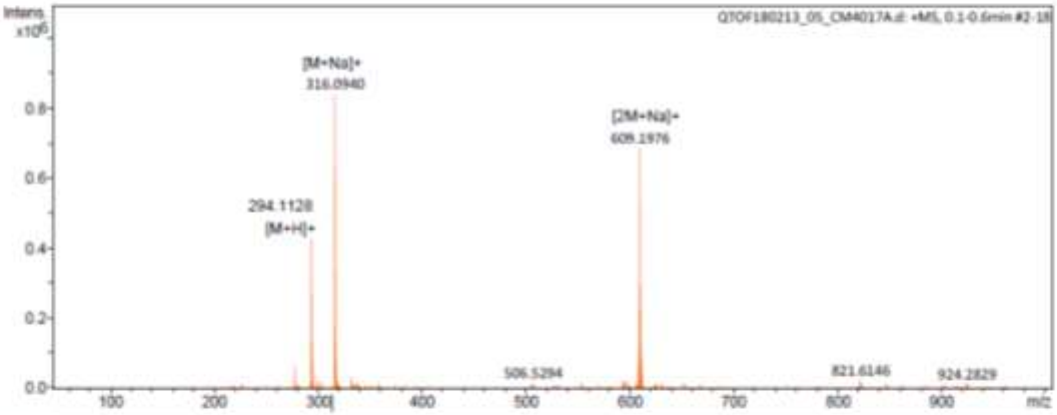

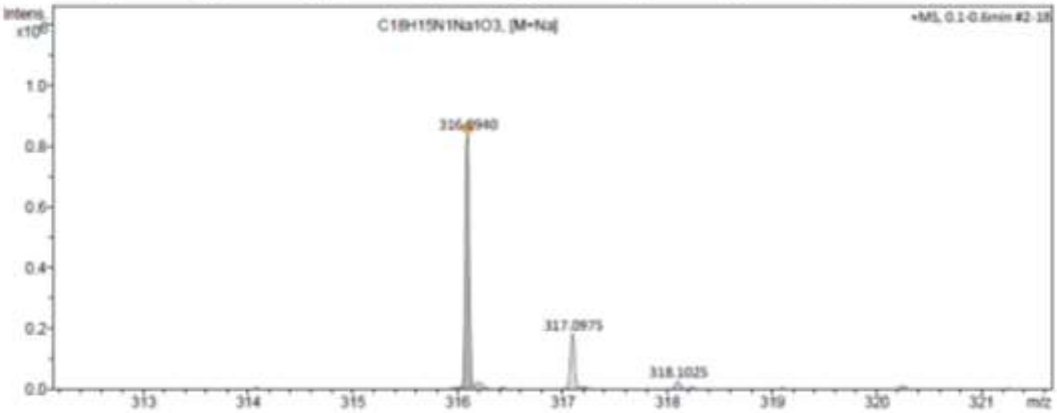

Meas. m/z	Ion Formula	Sum Formula	m/z	err [ppm]	mSigma	z	Adduct
294.1128	C18H16NO3	C18H15NO3	294.1125	-1.1	5.9	1+	M+H
316.0940	C18H15NNaO3		316.0944	1.3	8.2	1+	M+Na
609.1976	C36H30N2NaO6	C36H30N2O6	609.1996	3.3	21.6	1+	M+Na

CM3130B, MW=355.02

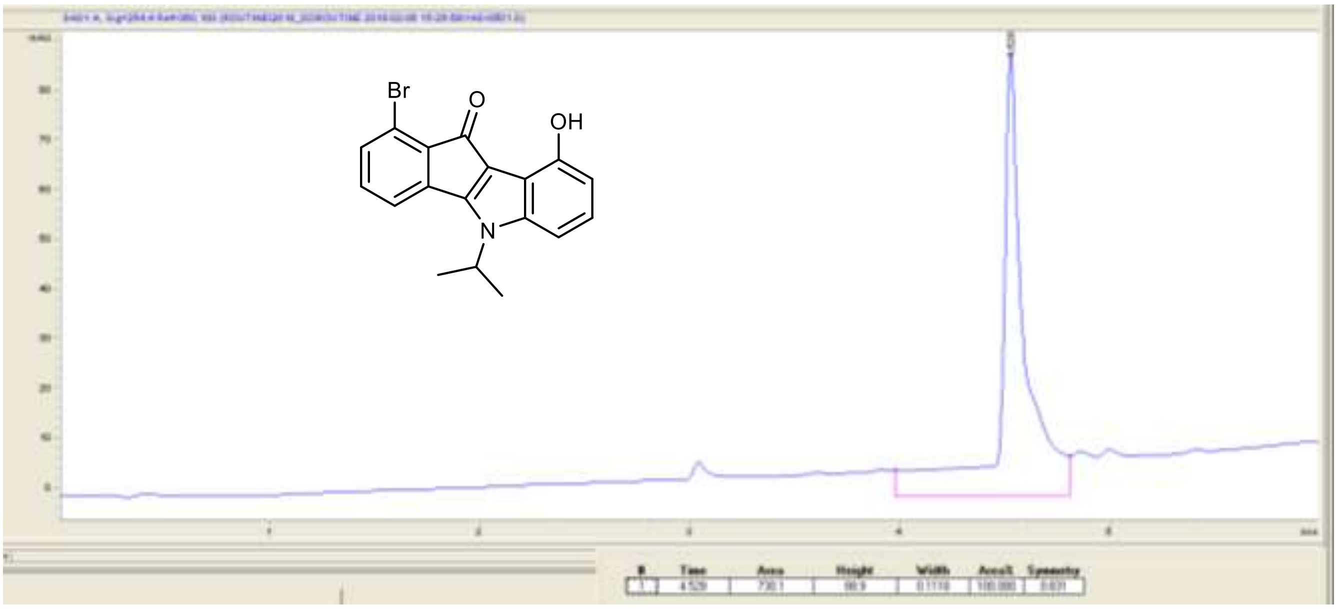

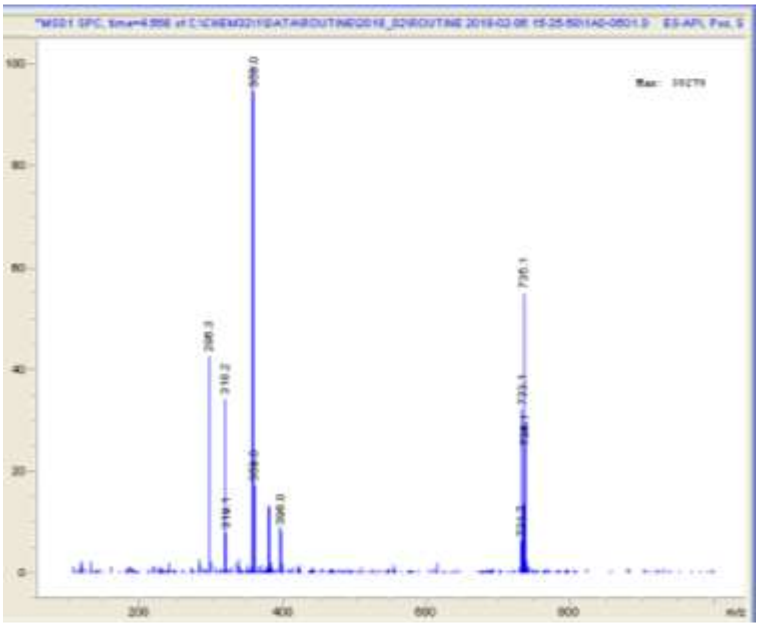

Analysis Info

Analysis Name	QTOF180213_06_CM3130B.d		
Method	2016_03_17_Infusion_50-1000_pos.m	Acquisition Date	2/13/2018 9:49:28 AM
Comment		Instrument / Ser#	micrOTOF-Q 228888.10231

Acquisition Parameter

Source Type	ESI	Ion Polarity	Positive	Set Nebulizer	0.4 Bar
Focus	Active	Set Capillary	3500 V	Set Dry Heater	200 °C
Scan Begin	50 m/z	Set End Plate Offset	-500 V	Set Dry Gas	4.0 l/min
Scan End	1000 m/z	Set Collision Cell RF	400.0 Vpp	Set Divert Valve	Waste

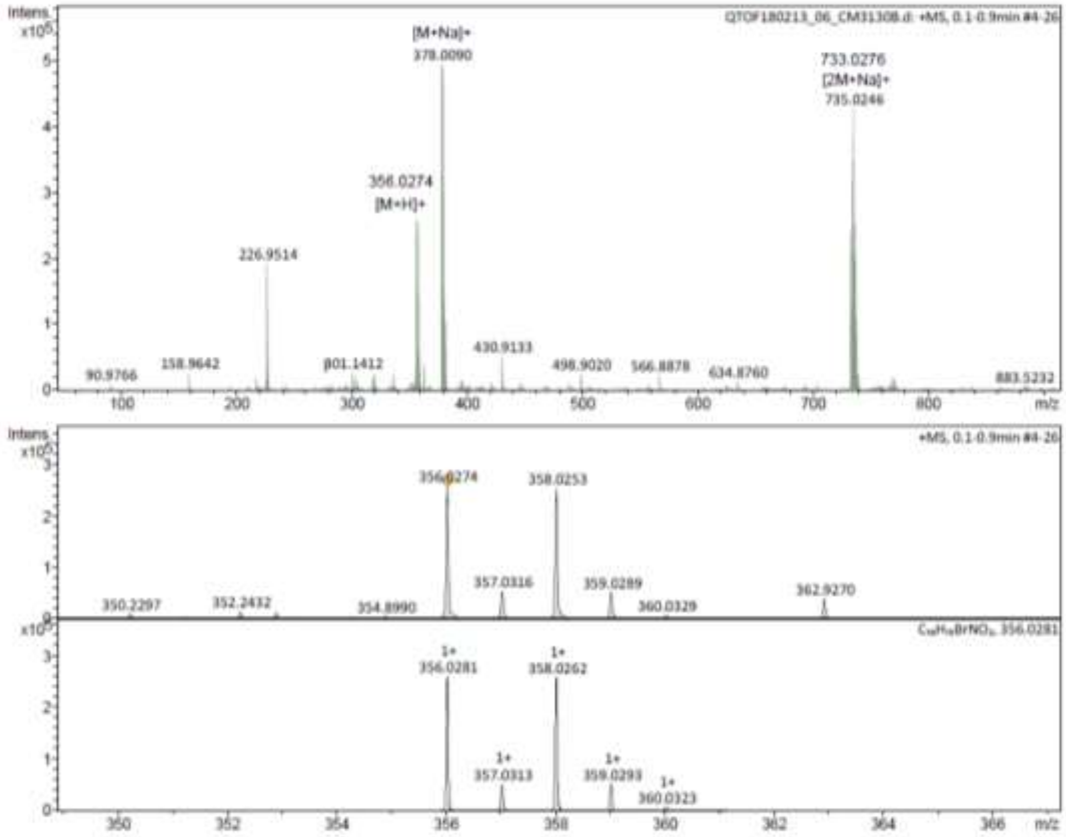

Meas. m/z	Ion Formula	m/z	err [ppm]	mSigma
356.0274	C18H15BrNO2	356.0281	1.8	6.0
378.0090	C18H14BrNNaO2	378.0100	2.8	11.7
733.0276	C36H28Br2N2NaO4	733.0308	4.4	52.0

CM4016A, MW=337.13

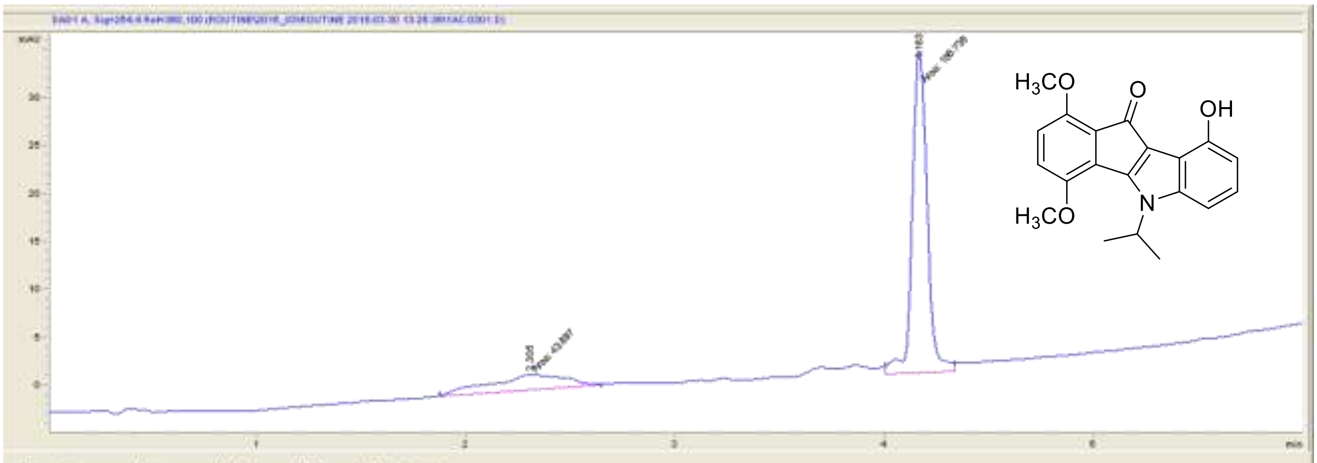

#	Time	Area	Height	Width	Area%	Symmetry
1	2.305	43.9	1.7	0.438	20.840	1.292
2	4.163	166.7	33.7	0.0824	79.160	0.832

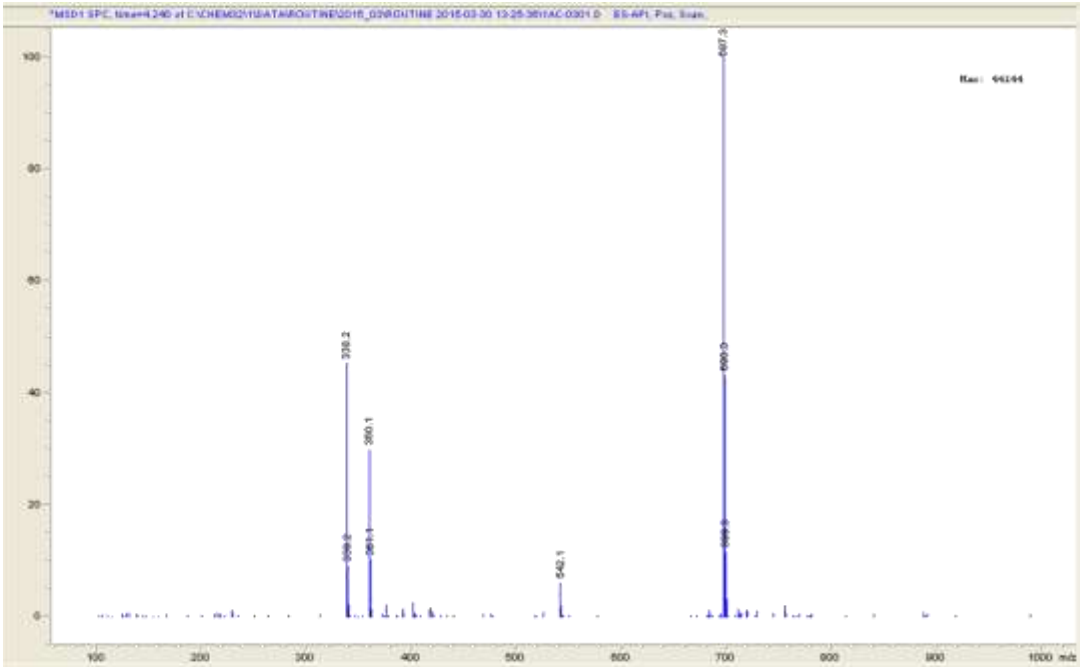

Analysis Info

Analysis Name	QTOF180213_04_CM4016A.d		
Method	2016_03_17_Infusion_50-1000_pos.m	Acquisition Date	2/13/2018 9:11:26 AM
Comment		Instrument / Ser#	micrOTOF-Q 228888.10231

Acquisition Parameter

Source Type	ESI	Ion Polarity	Positive	Set Nebulizer	0.4 Bar
Focus	Active	Set Capillary	1500 V	Set Dry Heater	200 °C
Scan Begin	50 m/z	Set End Plate Offset	-500 V	Set Dry Gas	4.0 l/min
Scan End	1000 m/z	Set Collision Cell RF	400.0 Vpp	Set Divert Valve	Waste

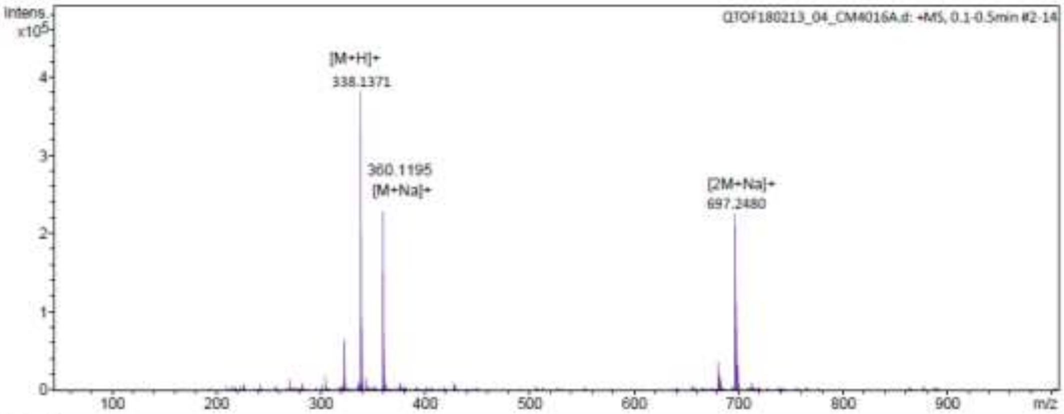

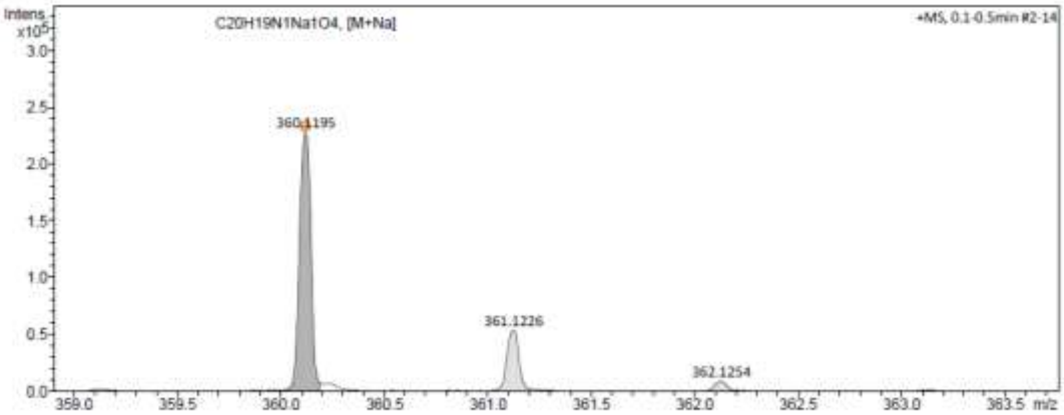

Meas. m/z	Ion Formula	Sum Formula	m/z	err [ppm]	mSigma	z	Adduct
338.1371	C20H20NO4	C20H19NO4	338.1387	4.7	11.9	1+	M+H
360.1195	C20H19NNaO4		360.1206	3.1	8.0	1+	M+Na

MF6, MW=337.13

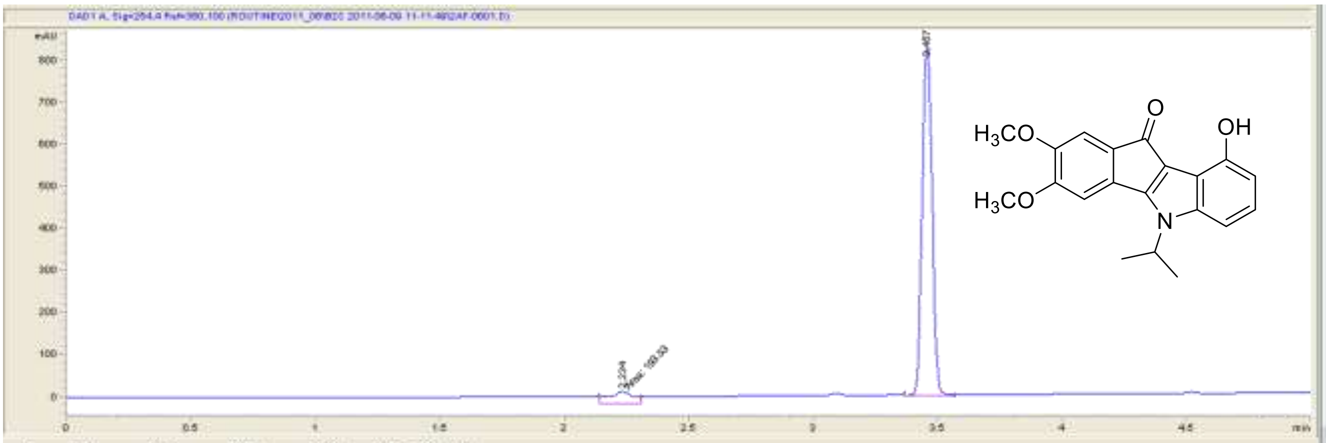

#	Time	Area	Height	Width	Area%	Symmetry
1	2.224	193.5	27.6	0.1167	7.692	1.062
2	3.457	2322.4	837.6	0.0443	92.308	0.949

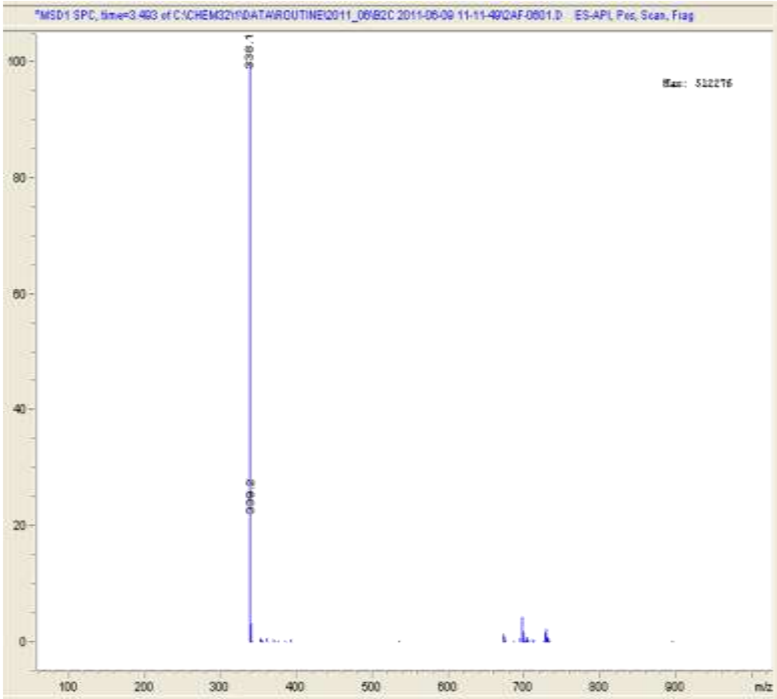

Analysis Info

Analysis Name	QTOF_150327_15_MF6.d		
Method	MS_inf_TL_50_1000_2014_woCollSweep_Pos_CCSM.m	Acquisition Date	3/27/2015 11:21:11 AM
Comment		Instrument / Ser#	micrOTOF-Q II 10231

Acquisition Parameter

Source Type	ESI	Ion Polarity	Positive	Set Nebulizer	0.4 Bar
Focus	Not active	Set Capillary	2000 V	Set Dry Heater	200 °C
Scan Begin	50 m/z	Set End Plate Offset	-500 V	Set Dry Gas	4.0 l/min
Scan End	1000 m/z	Set Collision Cell RF	140.0 Vpp	Set Divert Valve	Waste

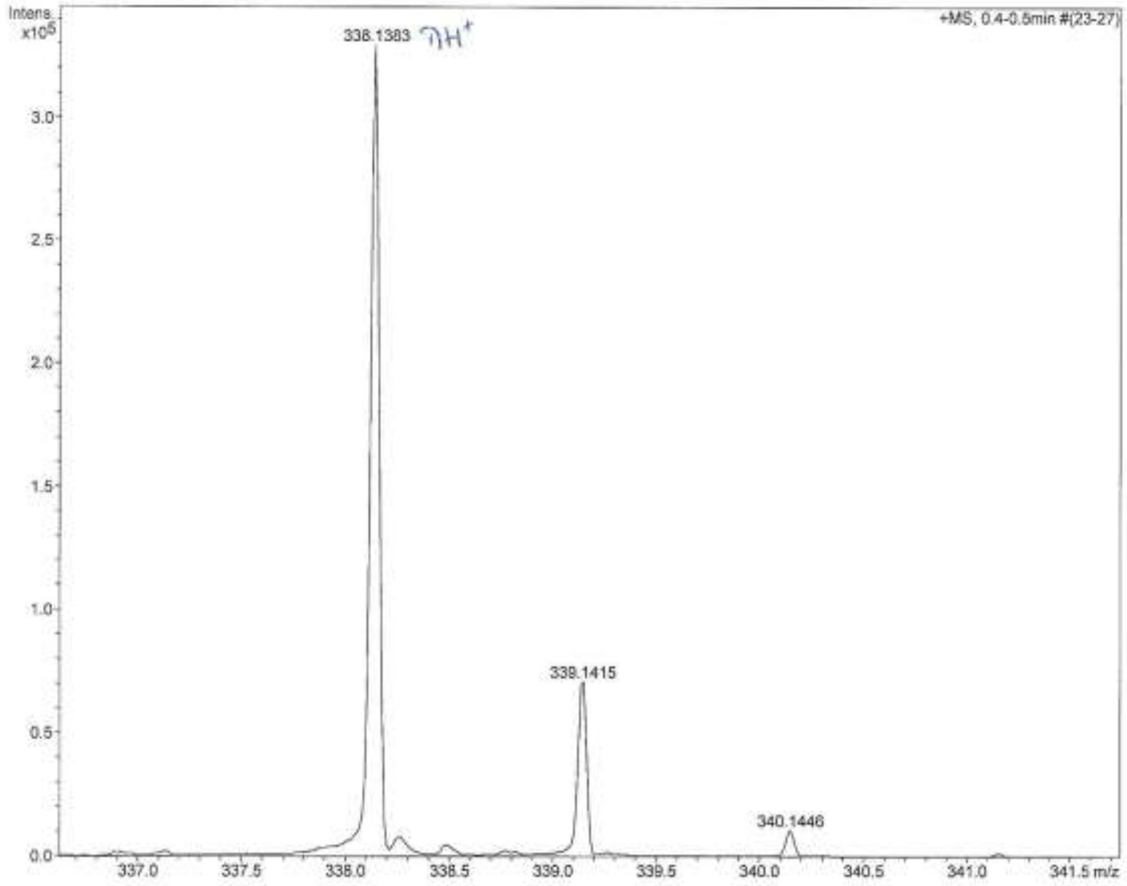

Meas. m/z	Formula	m/z	err [ppm]	mSigma
338.1383	C 20 H 20 N O 4	338.1387	1.0	2.8

CM3112B, MW=336.07

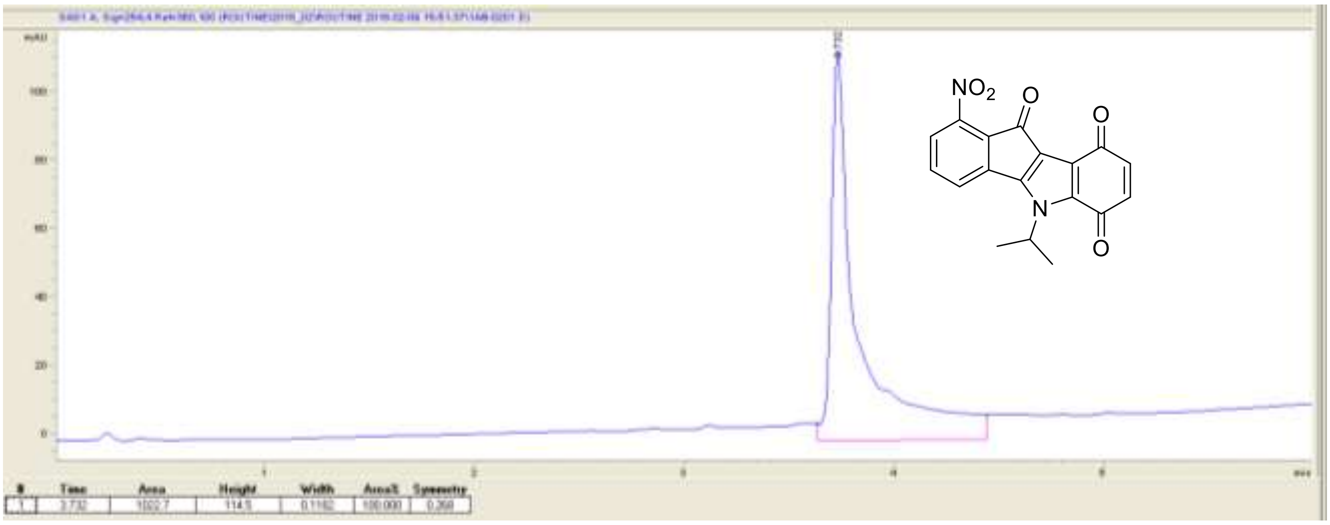

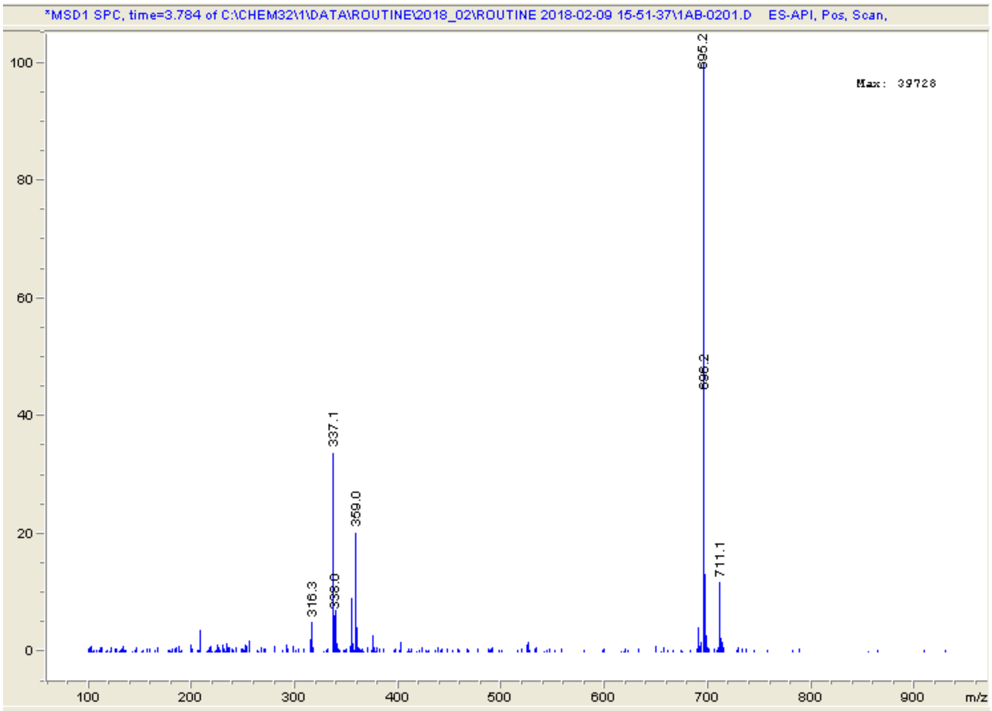

Analysis Info

Analysis Name	QTOF180213_02_CM3112B.d		
Method	2016_03_17_Infusion_50-1000_pos.m	Acquisition Date	2/13/2018 8:56:06 AM
Comment		Instrument / Ser#	micrOTOF-Q 228888.10231

Acquisition Parameter

Source Type	ESI	Ion Polarity	Positive	Set Nebulizer	0.4 Bar
Focus	Active	Set Capillary	1500 V	Set Dry Heater	200 °C
Scan Begin	50 m/z	Set End Plate Offset	-500 V	Set Dry Gas	4.0 l/min
Scan End	1000 m/z	Set Collision Cell RF	400.0 Vpp	Set Divert Valve	Waste

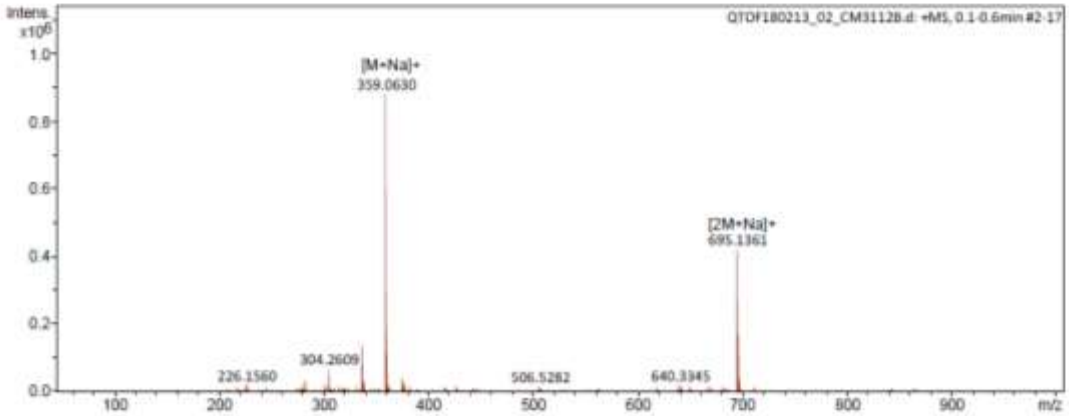

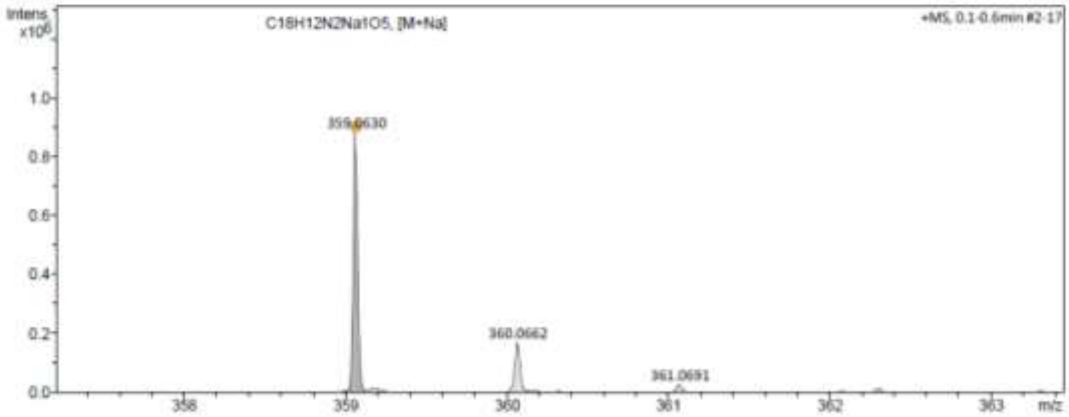

Meas. m/z	Ion Formula	Sum Formula	m/z	err [ppm]	mSigma	z	Adduct
359.0630	C18H12N2NaO5	C18H12N2O5	359.0638	2.5	6.9	1+	M+Na
695.1361	C36H24N4NaO10		695.1385	3.3	8.0	1+	2M+Na

The last seven indenoindole derivatives are described in this paper (**CM3159A**, **MF4**, **THN10**, **CM3072B**, **CM3159B**, **MF5**, **MF1**). All LC/HRMS data are given.

CM3159A, MW= 373.15

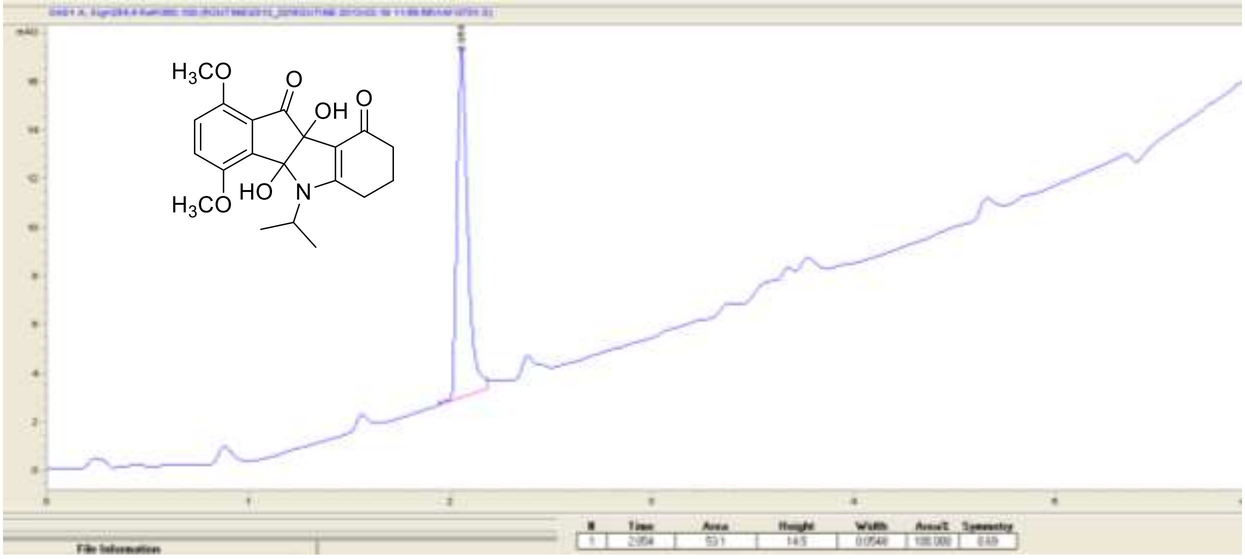

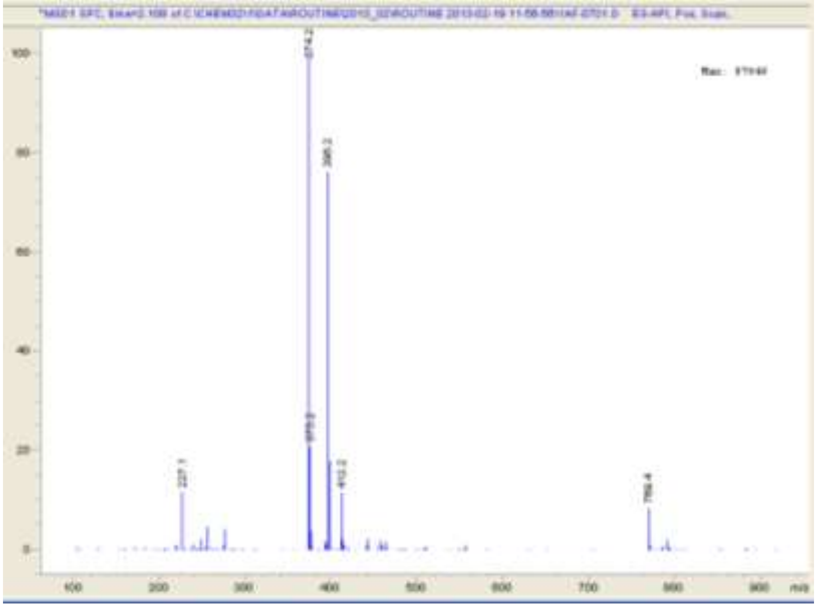

Analysis Info

Analysis Name	QTOF_150327_07_CM3157A.d		
Method	MS_inf_TL_50_1000_2014_woCollSweep_Pos_CCSM.m	Acquisition Date	3/27/2015 10:18:56 AM
Comment		Instrument / Ser#	micrOTOF-Q II 10231

Acquisition Parameter

Source Type	ESI	Ion Polarity	Positive	Set Nebulizer	0.4 Bar
Focus	Not active	Set Capillary	2000 V	Set Dry Heater	200 °C
Scan Begin	50 m/z	Set End Plate Offset	-500 V	Set Dry Gas	4.0 l/min
Scan End	1000 m/z	Set Collision Cell RF	140.0 Vpp	Set Divert Valve	Waste

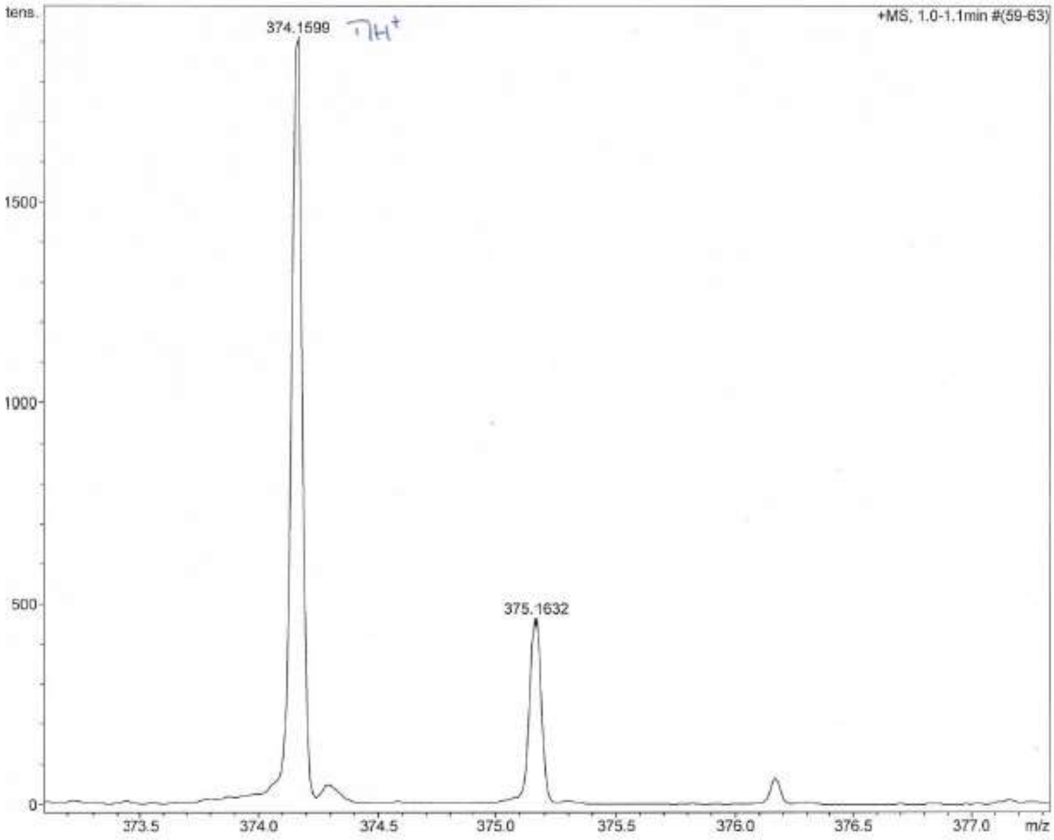

Meas. m/z	Formula	m/z	err [ppm]	mSigma
374.1599	C 20 H 24 N O 6	374.1598	-0.1	24.7

MF4, MW=373.15

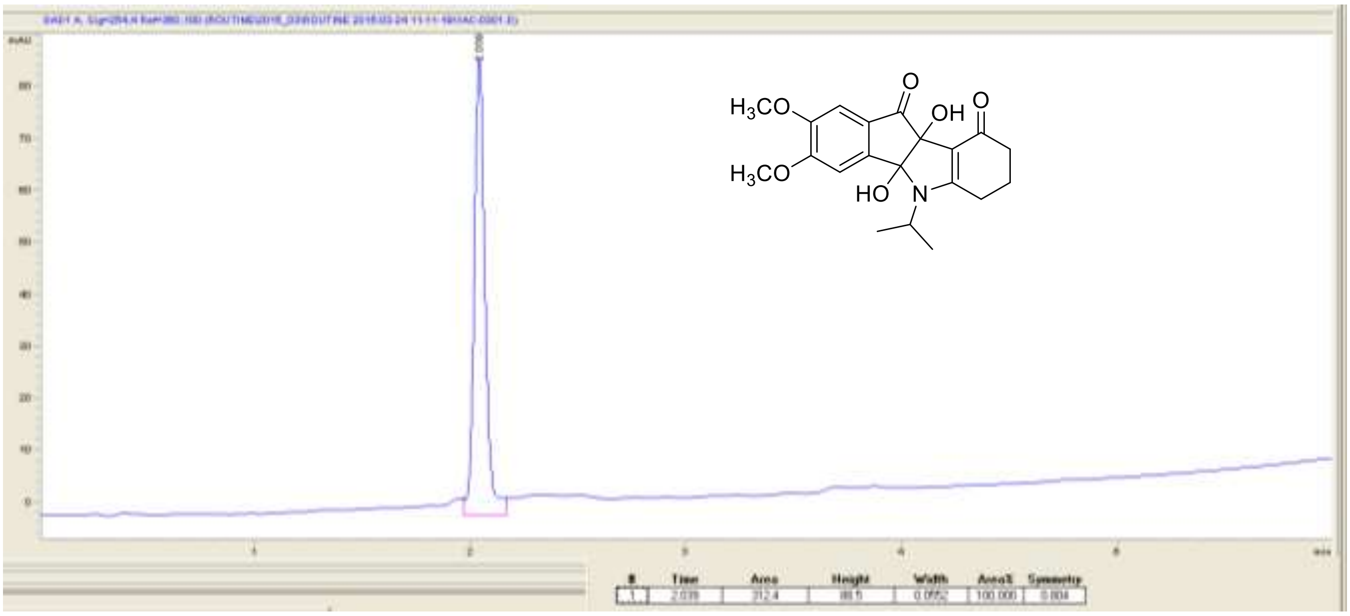
H3CO
H3CO
O
OH
O
HO
N

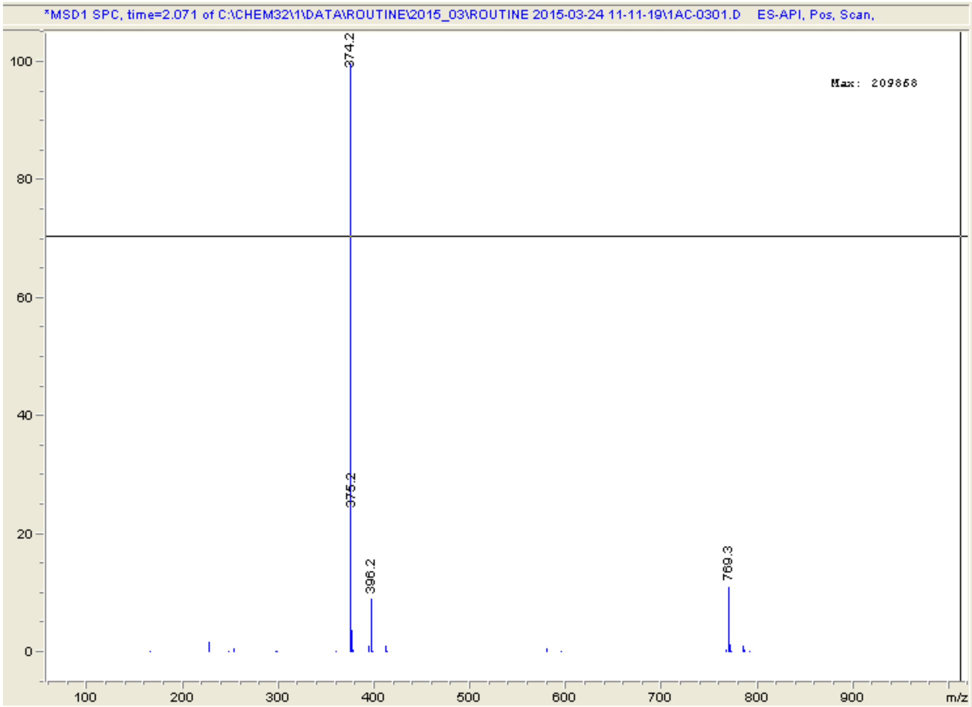
*MSD1 SPC, time=2.071 of C:\CHEM32\1\DATA\ROUTINE\2015_03\ROUTINE 2015-03-24 11-11-19\1AC-0301.D ES-API, Pos, Scan,
Max: 209868
374.2
375.2
396.2
769.3
m/z

Analysis Info

Analysis Name	QTOF_150327_06_MF4.d		
Method	MS_inf_TL_50_1000_2014_woCollSweep_Pos_CCSM.m	Acquisition Date	3/27/2015 10:11:26 AM
Comment		Instrument / Ser#	micrOTOF-Q II 10231

Acquisition Parameter

Source Type	ESI	Ion Polarity	Positive	Set Nebulizer	0.4 Bar
Focus	Not active	Set Capillary	2000 V	Set Dry Heater	200 °C
Scan Begin	50 m/z	Set End Plate Offset	-500 V	Set Dry Gas	4.0 l/min
Scan End	1000 m/z	Set Collision Cell RF	140.0 Vpp	Set Divert Valve	Waste

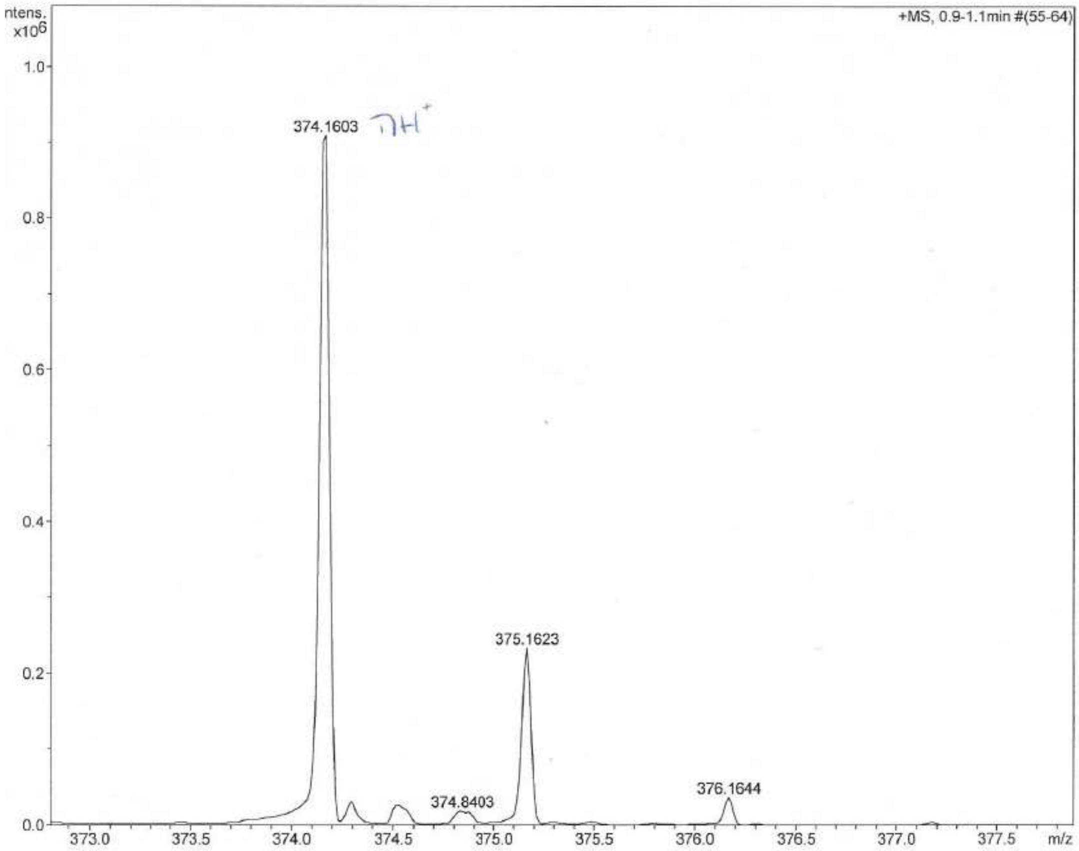

Meas. m/z	Formula	m/z	err [ppm]	mSigma
374.1603	C 20 H 24 N O 6	374.1598	-1.2	21.1

THN10, MW=309.14

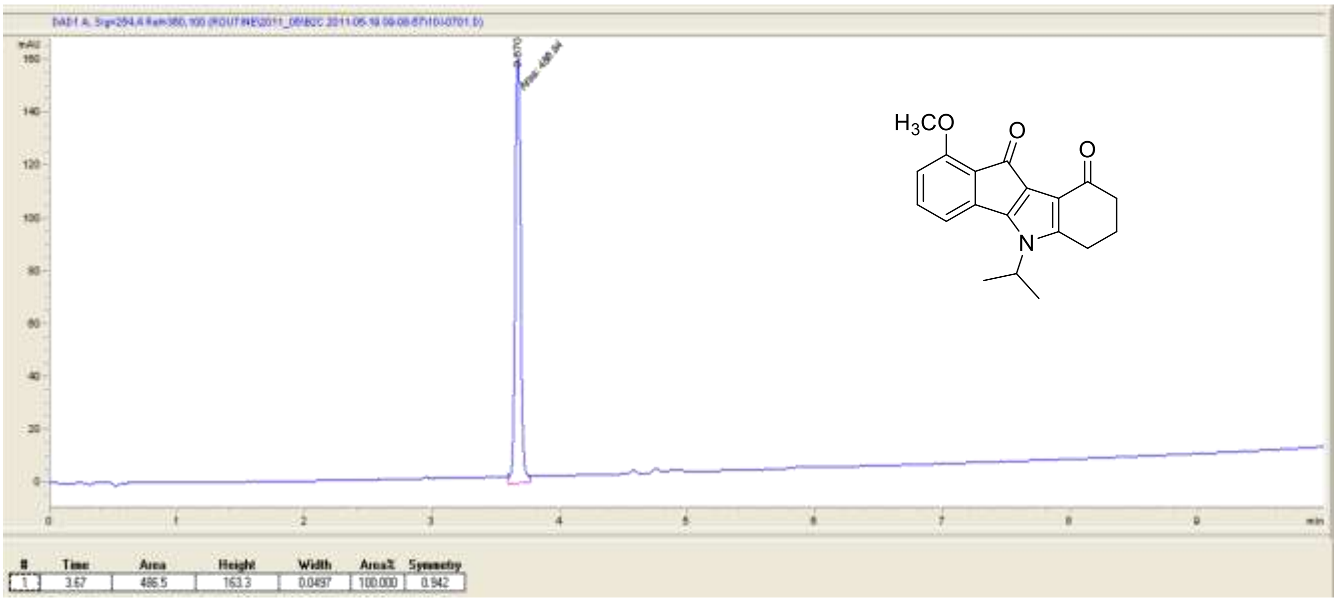

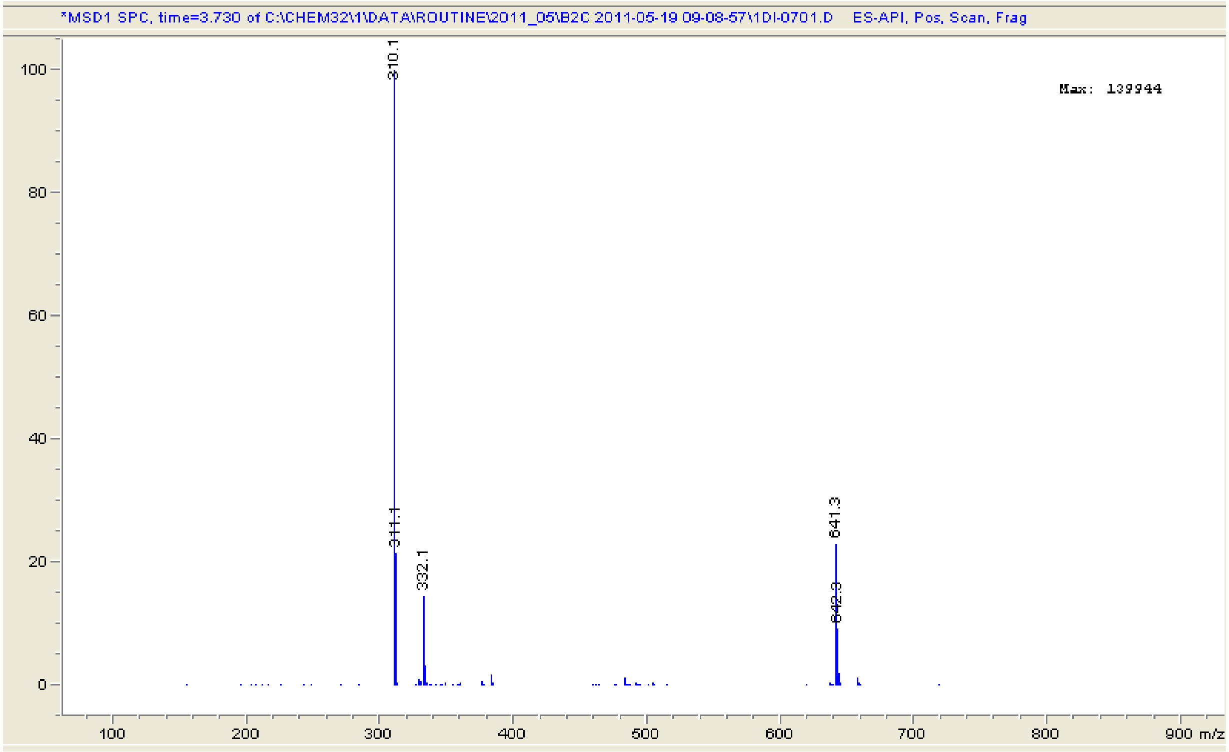

Analysis Info

Analysis Name	QTOF_150327_12_MF26D.d		
Method	MS_Inf_TL_50_1000_2014_woCollSweep_Pos_CCSM.m	Acquisition Date	3/27/2015 11:05:18 AM
Comment		Instrument / Ser#	micrOTOF-Q II 10231

Acquisition Parameter

Source Type	ESI	Ion Polarity	Positive	Set Nebulizer	0.4 Bar
Focus	Not active	Set Capillary	2000 V	Set Dry Heater	200 °C
Scan Begin	50 m/z	Set End Plate Offset	-500 V	Set Dry Gas	4.0 l/min
Scan End	1000 m/z	Set Collision Cell RF	140.0 Vpp	Set Divert Valve	Waste

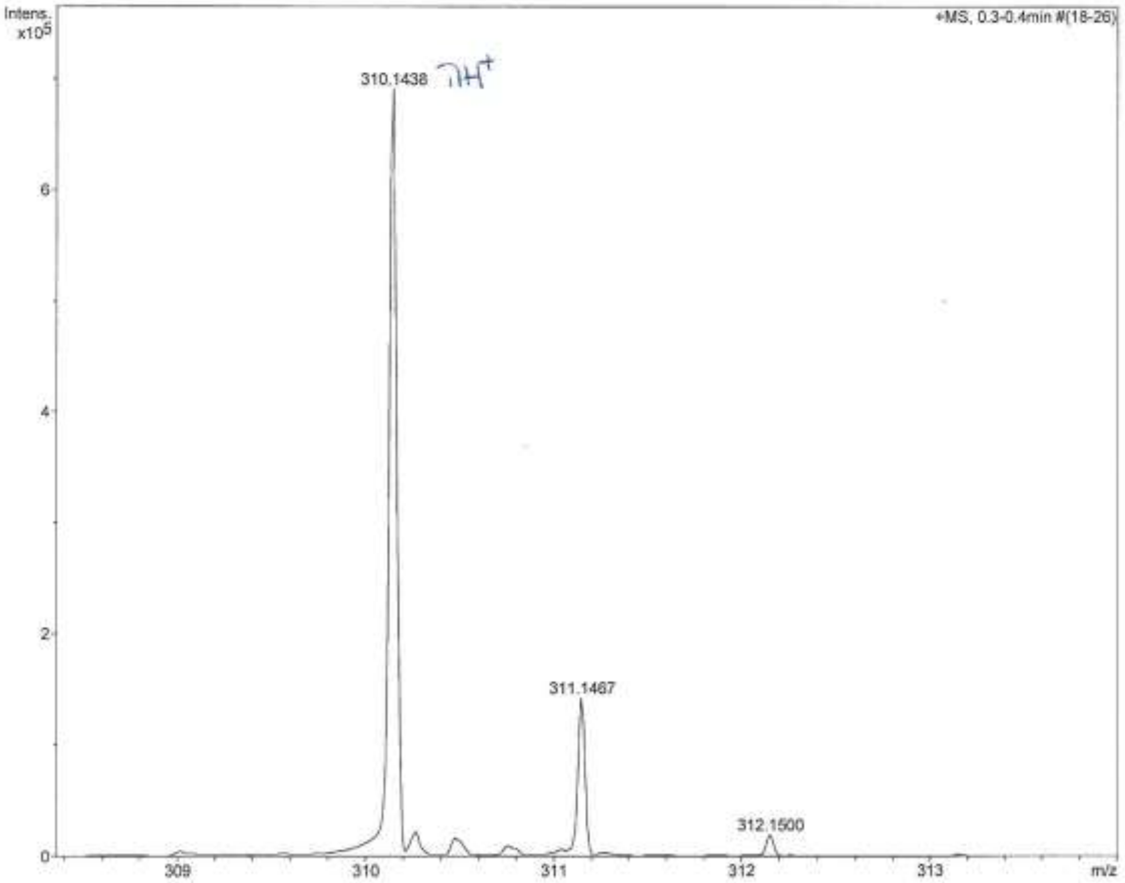

Meas. m/z	Formula	m/z	err [ppm]	mSigma
310.1438	C 19 H 20 N O 3	310.1438	-0.1	1.6

CM3072B, MW=309.14

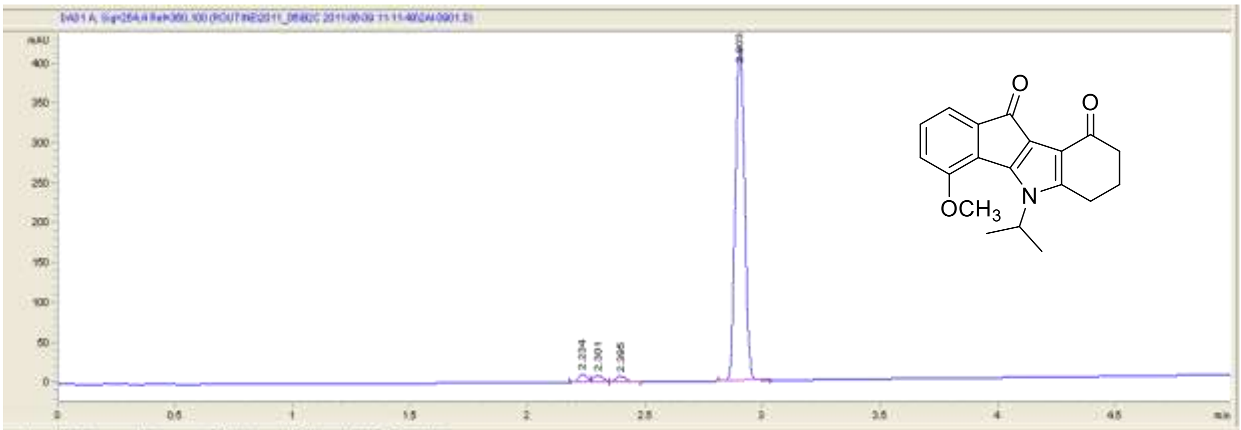

#	Time	Area	Height	Width	Area%	Symmetry
1	2.234	24.4	9.3	0.0427	2.023	1.025
2	2.301	21.2	7.8	0.0415	1.756	1.007
3	2.395	18.3	6.8	0.0433	1.517	0.967
4	2.903	1140.9	420	0.0437	94.704	0.954

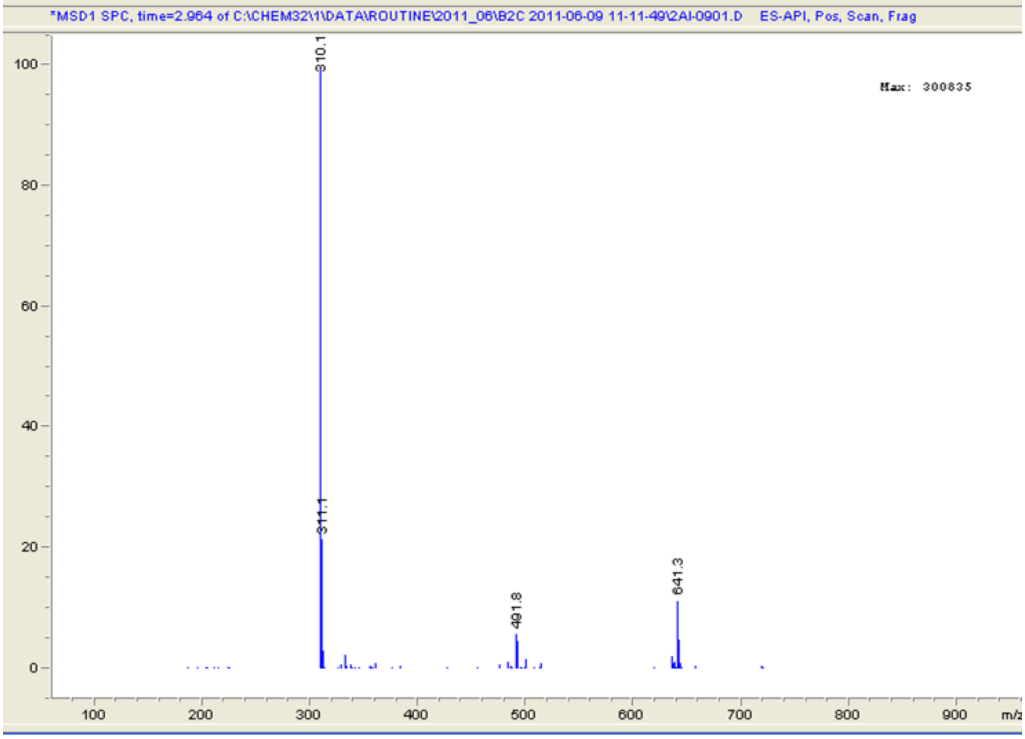

Analysis Info

Analysis Name	QTOF_150327_14_CM3072B.d		
Method	MS_inf_TL_50_1000_2014_woCollSweep_Pos_CCSM.m	Acquisition Date	3/27/2015 11:16:55 AM
Comment		Instrument / Ser#	micrOTOF-Q II 10231

Acquisition Parameter

Source Type	ESI	Ion Polarity	Positive	Set Nebulizer	0.4 Bar
Focus	Not active	Set Capillary	2000 V	Set Dry Heater	200 °C
Scan Begin	50 m/z	Set End Plate Offset	-500 V	Set Dry Gas	4.0 l/min
Scan End	1000 m/z	Set Collision Cell RF	140.0 Vpp	Set Divert Valve	Waste

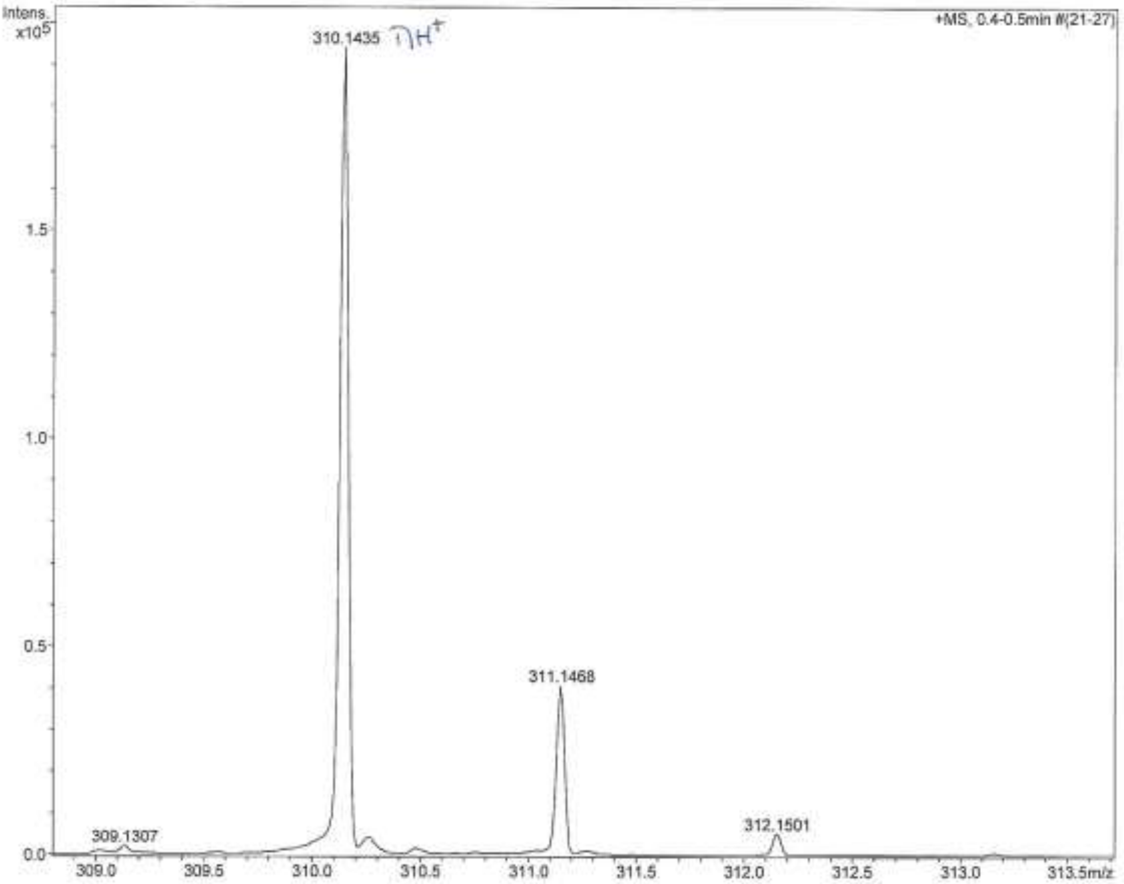

Meas. m/z	Formula	m/z	err [ppm]	mSigma
310.1435	C 19 H 20 N O 3	310.1438	0.7	0.6

CM3159B, MW=339.15

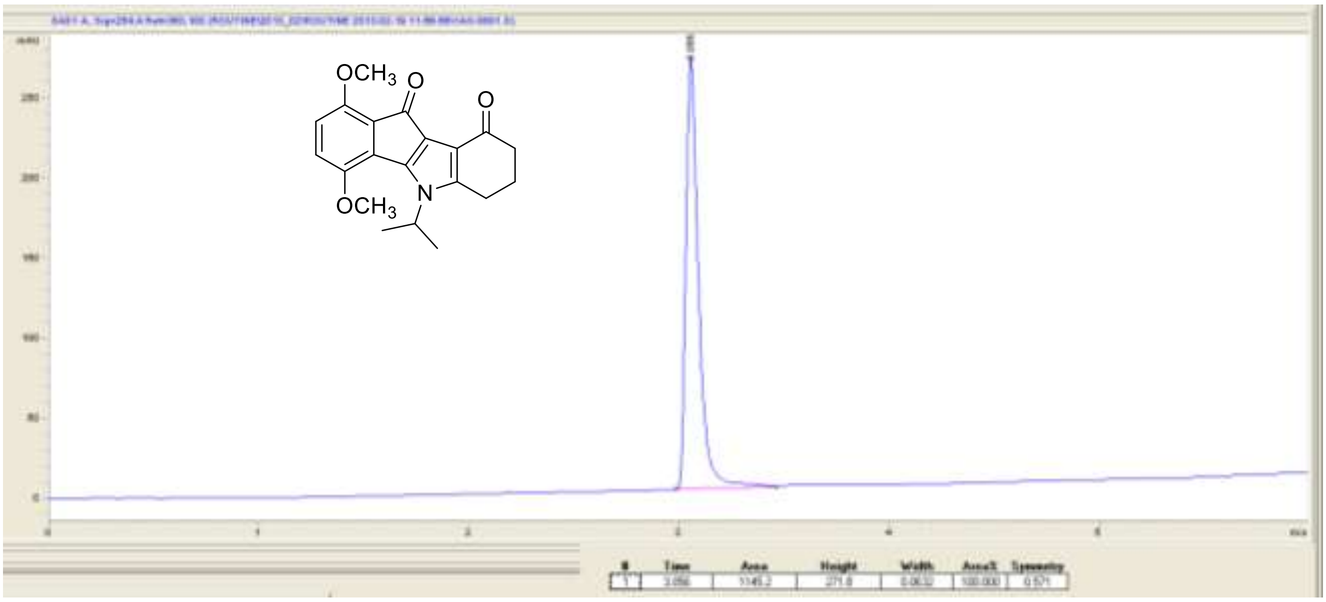

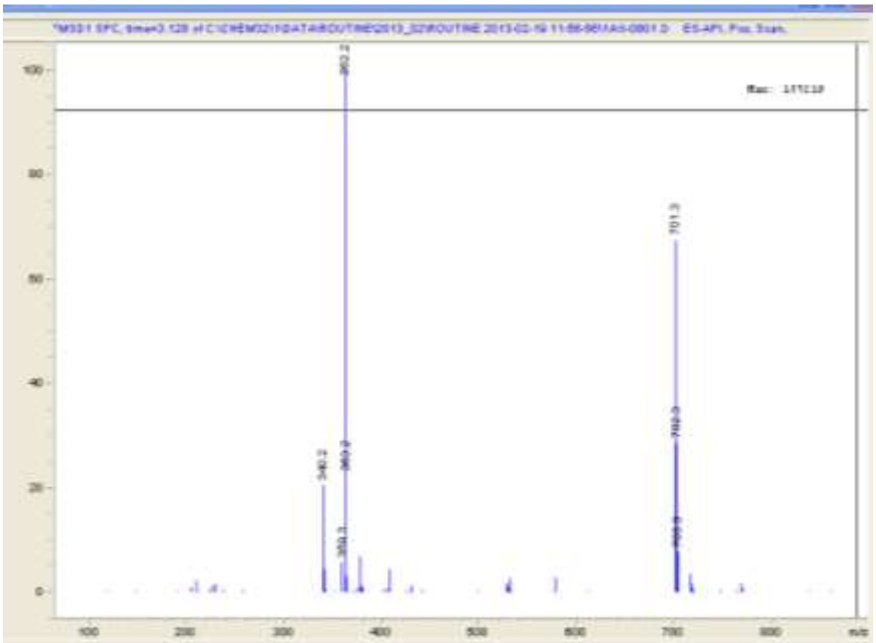

Analysis Info

Analysis Name	QTOF_150327_05_CM3157B.d		
Method	MS_inf_TL_50_1000_2014_woCollSweep_Pos_CCSM.m	Acquisition Date	3/27/2015 10:06:16 AM
Comment		Instrument / Ser#	micrOTOF-Q II 10231

Acquisition Parameter

Source Type	ESI	Ion Polarity	Positive	Set Nebulizer	0.4 Bar
Focus	Not active	Set Capillary	2000 V	Set Dry Heater	200 °C
Scan Begin	50 m/z	Set End Plate Offset	-500 V	Set Dry Gas	4.0 l/min
Scan End	1000 m/z	Set Collision Cell RF	140.0 Vpp	Set Divert Valve	Waste

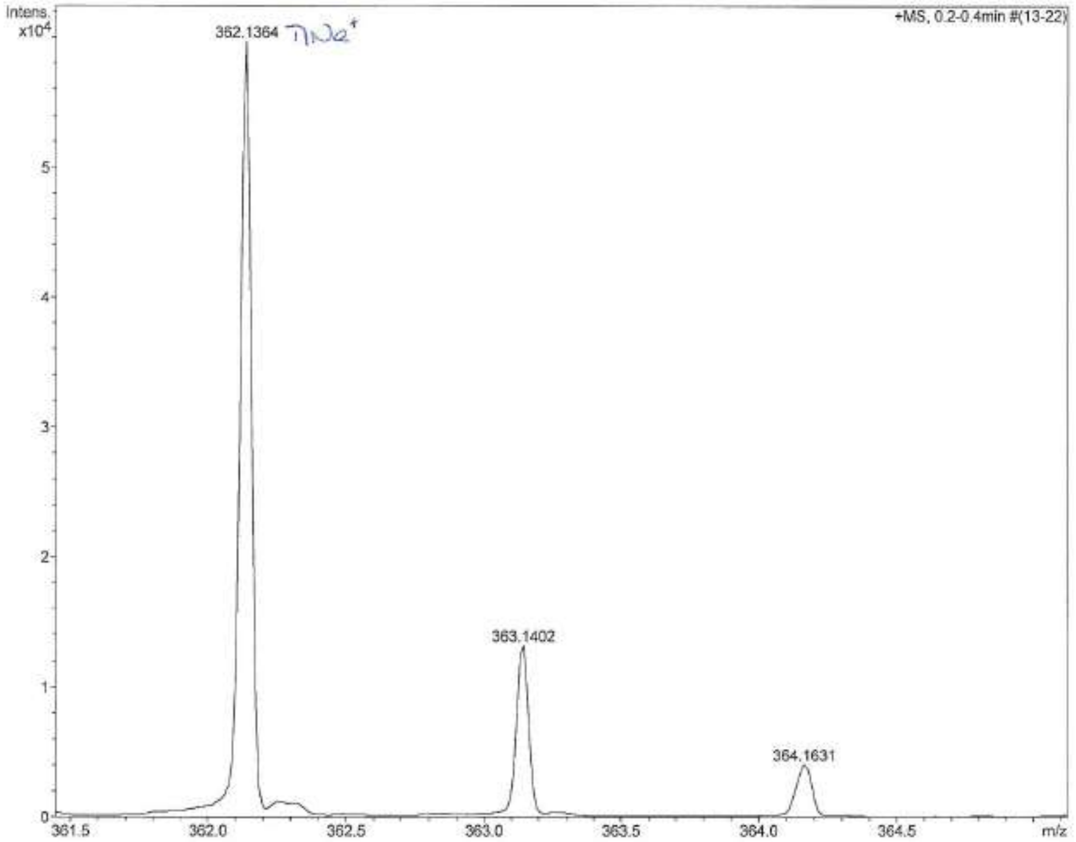

Meas. m/z	Formula	m/z	err [ppm]	mSigma
362.1364	C 20 H 21 N Na O 4	362.1363	-0.4	21.8

MF5, MW=339.15

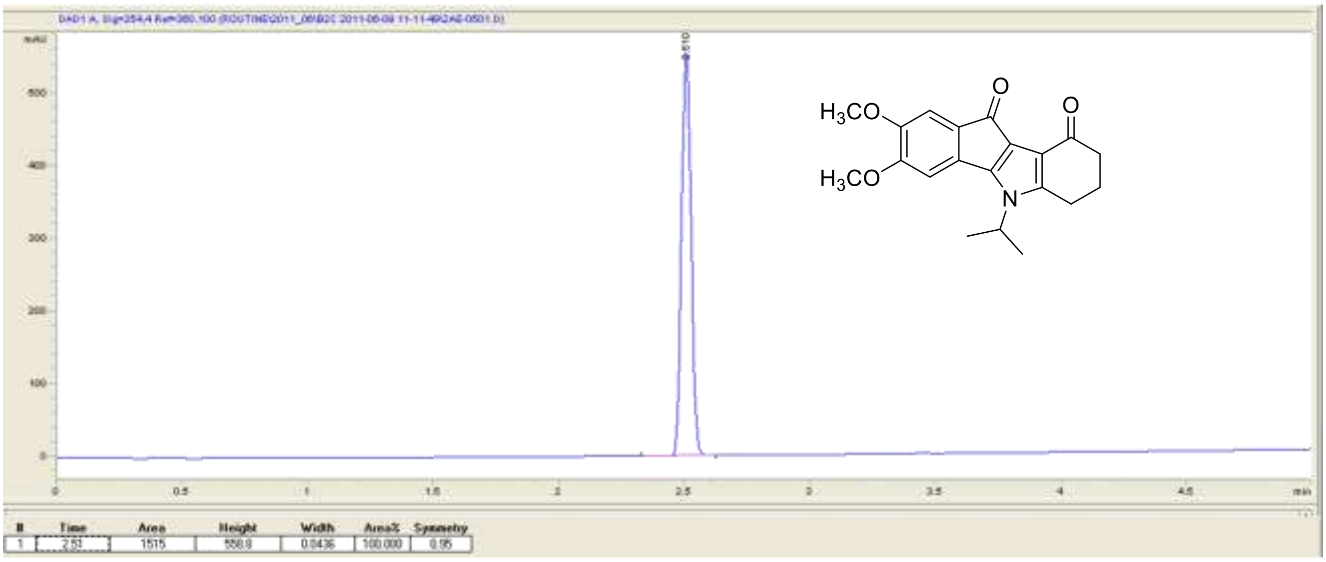

H3CO
H3CO
O
O
N
2.510
Time Area Height Width Area% Symmetry
1 2.51 1515 558.8 0.0436 100.000 0.95

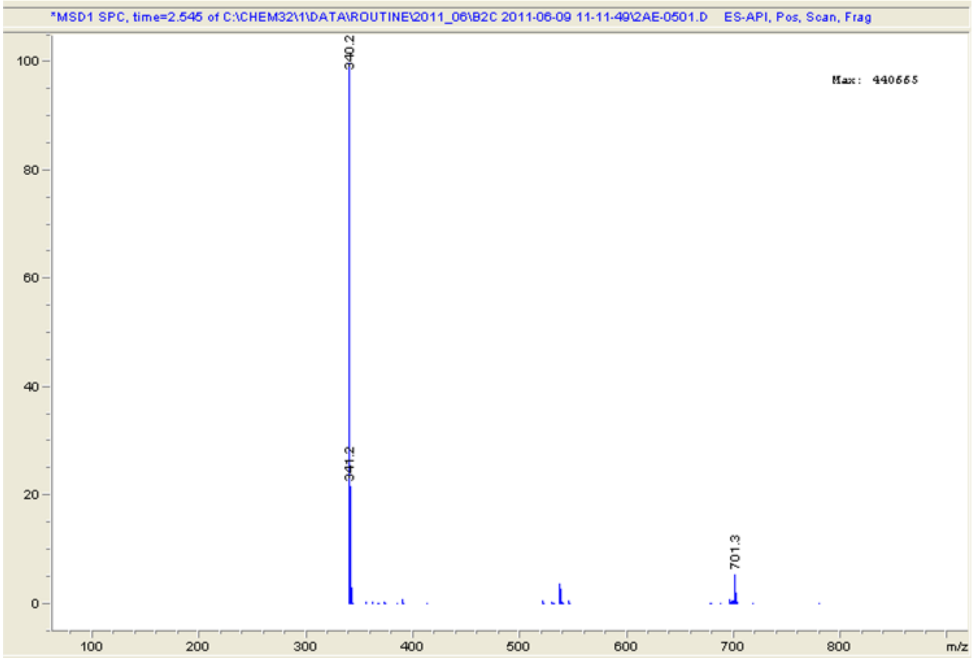

*MSD1 SPC, time=2.545 of C:\CHEM32\1\DATA\ROUTINE\2011_06\B2C 2011-06-09 11-11-49\2AE-0501.D ES-API, Pos, Scan, Frag
Max: 440665
340.2
341.2
701.3
m/z

Analysis Info

Analysis Name	QTOF_150327_08_MF5.d		
Method	MS_inf_TL_50_1000_2014_woCollSweep_Pos_CCSM.m	Acquisition Date	3/27/2015 10:24:14 AM
Comment		Instrument / Ser#	micrOTOF-Q II 10231

Acquisition Parameter

Source Type	ESI	Ion Polarity	Positive	Set Nebulizer	0.4 Bar
Focus	Not active	Set Capillary	2000 V	Set Dry Heater	200 °C
Scan Begin	50 m/z	Set End Plate Offset	-500 V	Set Dry Gas	4.0 l/min
Scan End	1000 m/z	Set Collision Cell RF	140.0 Vpp	Set Divert Valve	Waste

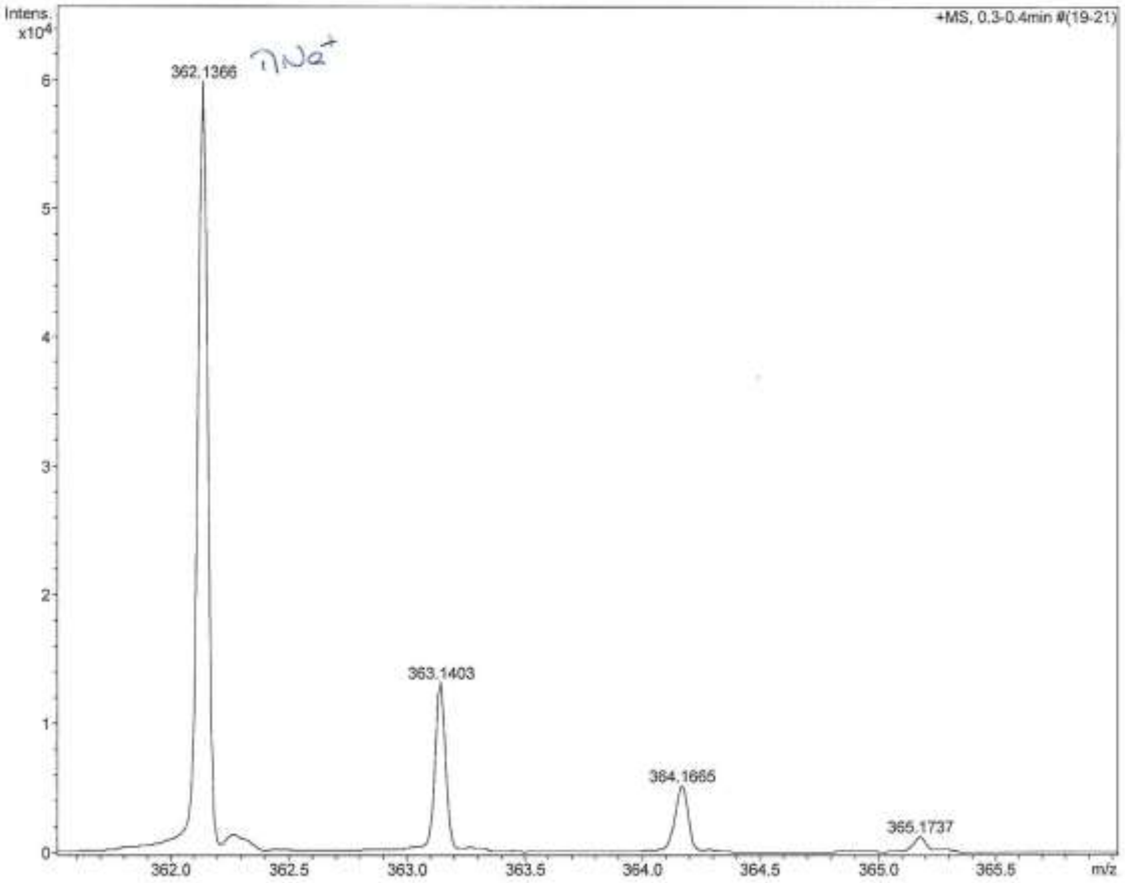

Meas. m/z	Formula	m/z	err [ppm]	mSigma
362.1366	C 20 H 21 N Na O 4	362.1363	-1.0	31.9

MF1, MW=294.14

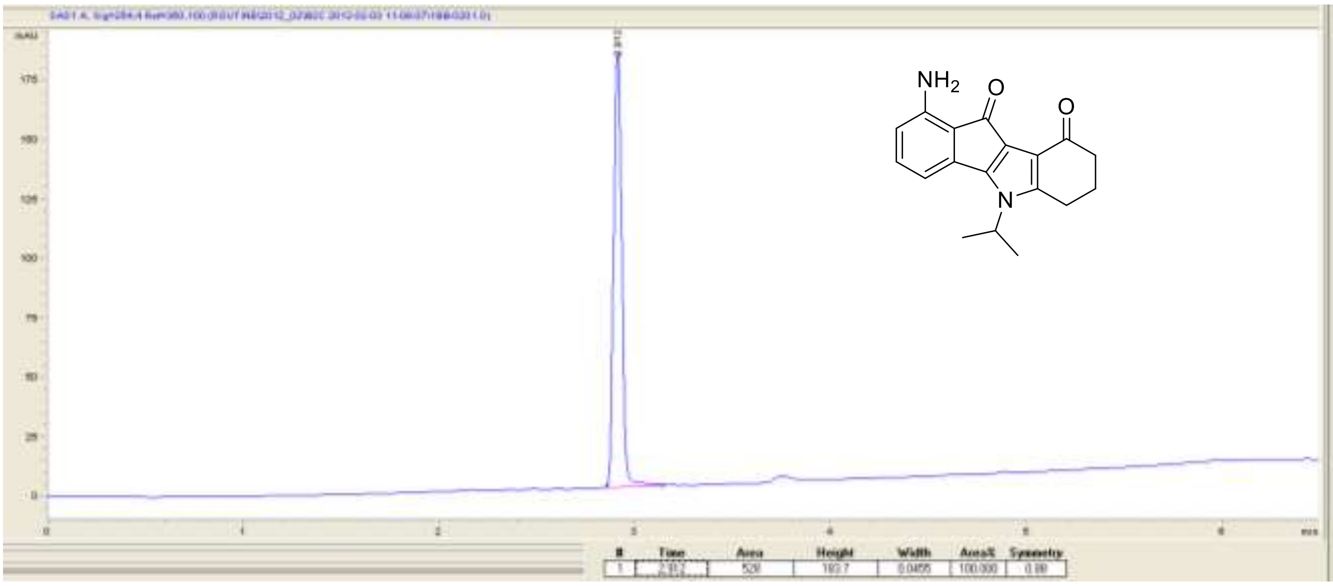
NH2
O
O
N
Time
Area
Height
Width
Symmetry

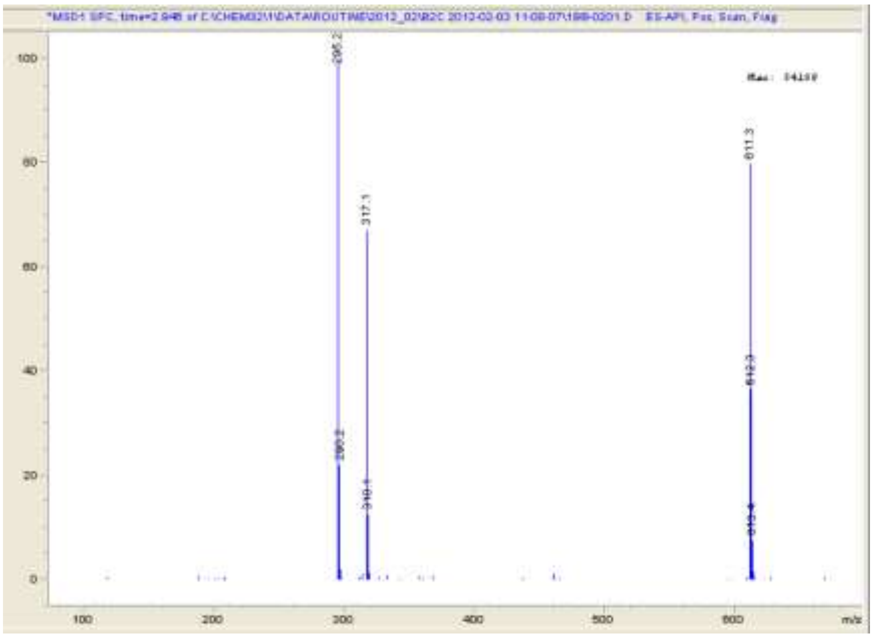
ES-API, Pos, Scan, Frag
295.2
317.1
611.3
612.3
m/z

Analysis Info

Analysis Name	QTOF_150327_09_MF1.d		
Method	MS_inf_TL_50_1000_2014_woCollSweep_Pos_CCSM.m	Acquisition Date	3/27/2015 10:30:34 AM
Comment		Instrument / Ser#	micrOTOF-Q II 10231

Acquisition Parameter

Source Type	ESI	Ion Polarity	Positive	Set Nebulizer	0.4 Bar
Focus	Not active	Set Capillary	2000 V	Set Dry Heater	200 °C
Scan Begin	50 m/z	Set End Plate Offset	-500 V	Set Dry Gas	4.0 l/min
Scan End	1000 m/z	Set Collision Cell RF	140.0 Vpp	Set Divert Valve	Waste

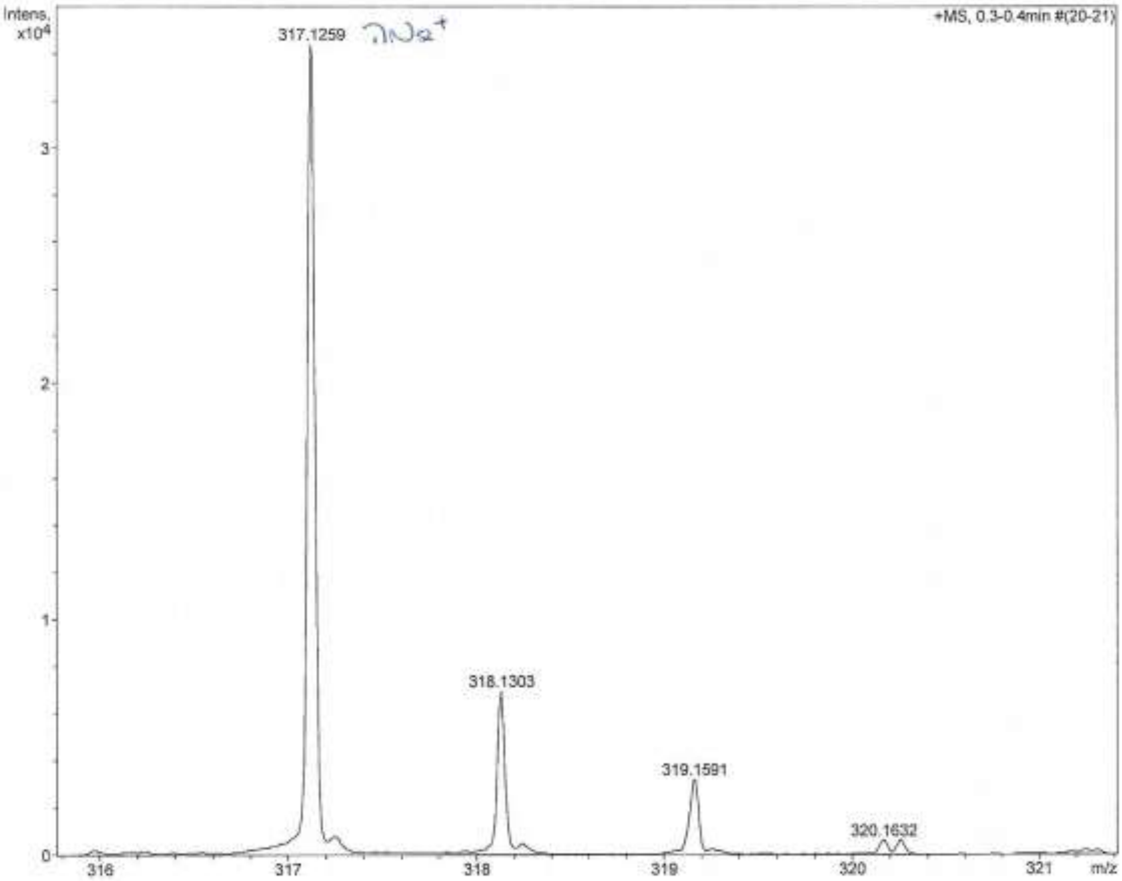

Meas. m/z	Formula	m/z	err [ppm]	mSigma
317.1259	C 18 H 18 N 2 Na O 2	317.1260	0.4	13.5
